# Kat5 cKO mouse replicates biological domain signatures associated with Alzheimer's disease

**DOI:** 10.1002/alz.71562

**Published:** 2026-06-30

**Authors:** Greg A Cary, Jessica E. Young, Shannon E. Rose, Harald Frankowski, Heather Wilkins, Sai Sruthi Amirtha Ganesh, Julia Draper, Martin Darvas, Mark Bothwell, Suman Jayadev, Aquene N. Reid, Anna Greenwood, Allan I. Levey, Karina Leal, Gregory W. Carter, Jesse C. Wiley

**Affiliations:** ^1^ The Jackson Laboratory Bar Harbor Maine USA; ^2^ University of Washington School of Medicine Seattle Washington USA; ^3^ University of Kansas Medical Center Kansas City Kansas USA; ^4^ University of Kansas Lawrence Kansas USA; ^5^ Sage Bionetworks Seattle Washington USA; ^6^ Emory University School of Medicine Atlanta Georgia USA

**Keywords:** Alzheimer's disease, biological domain, chromatin, endophenotype, epigenetics, genetics, proteomics, transcriptomics

## Abstract

**INTRODUCTION:**

Alzheimer's disease (AD) can be caused by autosomal‐dominant familial Alzheimer's disease (FAD) mutations in amyloid precursor protein (APP) or presenilin‐1 and 2, which form an enzyme substrate complex. KAT5 binds to the APP intracellular domain. Recent reports of decreased  γ‐secretase activity in FAD mutants support KAT5 membrane sequestration.

**METHODS:**

We compare the hippocampal transcriptome profiles of the Kat5 brain‐specific knockout (KO) mouse to multiple AD datasets through alignment with the TREAT‐AD AD biological domains. We examine KAT5 subcellular localization in human wild‐type and AD neurons.

**RESULTS:**

The Kat5 KO mouse demonstrates downregulation of synaptic genes, metabolic pathways, and upregulation of DNA replication and repair, cell cycle, and immune response genes. We see similar profiles in Kat5 and comparative AD datasets. KAT5 is restricted to the cytosol in human AD neurons.

**DISCUSSION:**

This analysis supports the hypothesis that KAT5 nuclear signaling downstream of APP cleavage plays a pivotal role in neuronal homeostasis.

## Highlights

- This study identified biological domain alignment and correlation between AD and Kat5 cKO neurons.
- The main overlapping biological domains are synapse, immune response, and endolysosome.
- Kat5‐mediated signaling may alter epigenetic signaling to maintain neuronal homeostasis.
- APP proteolytic deficits in FAD prevent Kat5 migration to the nucleus, preventing homeostatic epigenetic regulation.
- Neurodegenerative diseases may share disruption of common biological processes through distinct changes in specific genes that may be differentially epigenetically regulated.

## 1 BACKGROUND

The cloning of the β‐amyloid precursor protein (APP) sparked speculation about its potential role as a receptor, given its large extracellular domain and small intracellular domain that interacts with multiple signaling factors.^1^ However, the lack of an identified ligand or defined signaling mechanism cast doubt on this hypothesis, leaving APP's biological function unclear. Numerous studies suggest that APP plays a role in neurodevelopment, including synapse formation, axon pathfinding, and synaptic plasticity within developing cortical layers.^2^, ^3^, ^4^, ^5^, ^6^ Cultured neurons derived from APP‐null mice, while retaining amyloid beta precursor‐like protein 1 and 2 (APLP1 and APLP2), show a reduced number of synapses.^7^ Furthermore, the brain‐specific triple knockout of all three isoforms of the APP family cause profound alterations in synaptic signaling and plasticity along the performant path fibers, strongly indicating APP's role in synaptic function.^8^

APP is a type‐I single‐pass transmembrane protein sequentially cleaved in its extracellular domain by either α‐ or β‐secretase, followed by cleavage within the transmembrane region by the heterotetrameric γ‐secretase complex.^9^, ^10^ Mutations in APP near the β‐ and γ‐secretase cleavage sites, or within the presenilins (PSEN1 or PSEN2), lead to autosomal‐dominant Alzheimer's disease (AD).^1^ The γ‐secretase complex, with PSEN at its catalytic core, directly associates with APP and mediates its intramembranous aspartyl‐dependent proteolysis.^9^ Numerous reports show that familial AD (FAD) mutations often reduce overall proteolytic activity and sometimes decrease levels of major amyloid species.^11^, ^12^, ^13^, ^14^, ^15^ Additionally, some FAD mutations do not increase amyloid beta (Aβ) 42 production but instead lower Aβ40 levels or produce longer Aβ peptides predicted to remain embedded in the membrane.^16^, ^17^ Recent work demonstrates that FAD mutations in APP and PSEN1 stall the PSEN‐APP enzyme–substrate complex, decrease APP intracellular domain (AICD) production, and support the plausibility that FAD mutations could disrupt APP cleavage‐dependent signaling via bound intracellular factors.^18^

KAT5 binds to the intracellular region of APP through its association with FE65 and is known to play a critical role in regulating synaptic gene expression.^19^, ^20^, ^21^, ^22^, ^23^ KAT5 is a major histone acetylase in brain that migrates to the nucleus following γ‐secretase‐mediated APP cleavage,^24^, ^25^, ^26^ suggesting that neuronal epigenetic regulation may be controlled by this critical proteolytic event. Further support for this hypothesis comes from the observations that brain‐specific knockouts (Kos) of KAT5 or PS1/PS2 result in dysregulation of synaptic gene expression and neurodegeneration greatly resembling AD.^23^, ^27^ Elevated histone deacetylation is implicated in AD,^20^, ^28^ so a decrement in KAT5‐mediated histone acetylase activity could promote hypoacetylation, decreasing epigenetic regulation involved in synaptic plasticity.^22^, ^29^

In this study, we propose that decreased γ‐secretase‐mediated APP cleavage results in greater retention of intracellular bound factors at the membrane, causing KAT5 to become sequestered at the membrane, repressing nuclear signaling. If APP/KAT5 nuclear signaling plays a role in AD pathogenesis, we would expect to see similar transcriptomic signatures in the KAT5 brain‐specific inducible KO mouse and AD‐associated transcriptomic profiles. The brain‐specific inducible Kat5 knockout mouse (Kat5 cKO) was generated as previously reported.^23^ We employ the transcriptomic data generated in this work in a cross‐species comparison employing the informatics data‐harmonization approaches developed within the TREAT‐AD consortium, most notably the AD biological domain and subdomain enrichment methodologies, to capture the similarity of key disease‐related processes.^30^ In this work we leverage extensive efforts by MODEL‐AD, AMP‐AD, and TREAT‐AD in the development of human and murine transcriptomic datasets, as well as focal studies interrogating specific disease subtypes and murine models for the greatest possible breadth of analysis. We hypothesize that if KAT5 were involved in APP signaling to maintain synaptic homeostasis, then we would see strong correlation across datasets within core biological areas impacted by AD. The comparison of Kat5 cKO with human and mouse neurodegenerative disease models shows a similarity with Kat5 cKO and AD, with some overlap with other neurodegenerative diseases. We further demonstrate that KAT5 is restricted from the nucleus specifically in human AD neurons. These results support a plausible role for disrupted KAT5 nuclear signaling in AD pathogenesis.

**RESEARCH IN CONTEXT**

1. **Systematic review**: The TREAT‐AD Consortium has compiled the major AD multiomic datasets and extracted the primary endophenotypes into the AD biological domains in order to map large‐scale data to interpretable biology. We use these resources to explore a hypothesis emerging from multiple lines of gamma‐secretase biochemistry suggesting that genetic FAD mutations result in a decrease in APP cleavage, which could prevent bound KAT5 from migrating to the nucleus. KAT5 is essential for neuronal survival, suggesting a plausible role in regulating the progression of neurodegeneration.
2. **Interpretation**: Our data support the hypothesis that KAT5 migration to the nucleus pursuant to gamma‐secretase‐mediated APP proteolysis may play a neuronal homeostatic role that is deleteriously impacted in AD pathoprogression.
3. **Future directions**: The exploration of this hypothesis will involve a careful delineation of whether increasing or decreasing KAT5 nuclear activity will promote resistance or resilience to dementia progression and neuronal degeneration.

## 2 METHODS

### 2.1 Datasets and data availability

The datasets selected are the best available harmonized AD transcriptomic collections across human neurodegenerative diseases (FAD, frontotemporal dementia [FTD], Parkinson's disease [PD], amyotrophic lateral sclerosis [ALS], and late‐onset AD [LOAD]). We borrowed from the extensive work done within MODEL‐AD in the characterization of numerous AD‐related mouse models, as species‐specific comparators to the KAT5 cKO, as we anticipated that there may be greater similarity within disease models in the same species. We compared the different human AD datasets to represent broad classes of archetypal AD, both genetic and typical LOAD, in order to assess potential relevance to common human disease pathology. We employed other neurodegenerative disease models to assess how much of the disease signatures may be common across neurodegeneration.

### 2.2 Kat5 cKO mouse model data

The Kat5 mouse RNA‐sequencing (RNA‐seq) model data were extracted from the published report from the Eichle laboratory.^23^ The dataset contained the complete gene expression profile observed within the Kat5 forebrain‐specific tamoxifen‐inducible CamKIIα promoter‐driven CRE mouse crossed onto the floxed Kat5 mouse. The CA1 region of the hippocampus was laser dissected after 10 days of tamoxifen induction of the CRE‐recombinase in 2‐month‐old mice. The dataset included six experimental (CamKII‐CRE X Kat5_flox_) and six control (Kat5_flox_) mice, both treated with tamoxifen to control for non‐specific drug treatment effects. The fold‐change calculations were made across all genes, and statistical significance was determined using an adjusted *p* value threshold of 0.05 independent of fold‐change level. The dataset is parsed into low (1.2‐fold), medium (1.5‐fold), and high (2‐fold) levels of up‐ or downregulation. The analysis performed assessing global shifts in transcriptomic signatures used the 1.2‐fold change group, to attain the highest level of biological process sensitivity.

### 2.3 Human early‐onset AD and familial AD datasets

The early‐onset AD (EOAD) and FAD human datasets were extracted from Antonell et al.,^31^ in which sporadic EOAD patients and FAD patients were interrogated *post mortem* to compare the transcriptomic profiles of these two types of AD. Seven subjects were employed in the sporadic EOAD (under 65 years of age), PSEN1 FAD (M139T), and the control group, with a preponderance of males in all groups: 5/7, 6/7, and 4/7, respectively. The average age of the participants varies from 50 for the controls, 54 for the FAD participants, and 63 for the sporadic AD patients. The *APOE* allele was predominantly *APOE* ε3 across subject groups with a single ε3/ε4 heterozygous participant in each study group. The neuropathological rating was performed using the ABC scale (A: Thal Amyloid rating, B: Braak tau stage, C: Consortium to Establish a Registry for Alzheimer's Disease amyloid presence), and the sporadic EOAD and FAD subjects rated severe (A3, B3, and C3) across pathology measures – the controls had no noted pathology. RNA was extracted from the posterior cingulate cortex, purified, and quality control assessed with a high value of the RNA integrity number (RIN) across samples, average 7.3 for experimental groups and 7.2 for control participants. The labeled RNA was hybridized to an Affymetrix ST 1.1 Human Gene chip, and the data were cleaned and compared between control and experimental groups using Bioconductor R packages developed for Affymetrix analysis and LIMMA packages.^31^ The differential gene expression was identified using pairwise comparisons between experimental and control groups and corrected for false discovery using Benjamini and Hochberg methods. The data can be accessed at Gene Expression Omnibus (GEO) accession GSE39420. For additional details, see the original characterization.^31^

### 2.4 Human LOAD dataset

The LOAD dataset was developed from multiple brain banks representing large independent research efforts within AMP‐AD and TREAT‐AD. We pulled the transcriptomic datasets together within our bioinformatics pipeline analysis to facilitate TREAT‐AD target evaluation and grouping into the biological domains developed to represent the AD endophenotypes in an objective fashion.^30^ The compiled datasets are currently available on the AD Knowledge Portal.^30^ The transcriptomic dataset was drawn from the Religious Orders Study/Memory and Aging Project (ROSMAP), Mayo, and Mount Sinai brain banks, comprising over 1700 individual brains across genders, ethnicities, and age brackets. No subordinate evaluation of the subpopulations within the collective dataset was initially performed, so only the collective dataset was analyzed and compared to the Kat5 cKO model in the present work.

### 2.5 The 5xFAD mouse

The 5xFAD transgenic mouse strain carries five distinct APP and PSEN1 FAD mutations that drive elevated amyloid production and memory deficits early in mouse development.^32^ The 5xFAD mouse transcriptome is employed and rigorously evaluated in the MODEL‐AD consortium efforts.^33^, ^34^, ^35^ The transcriptomic dataset for the 12‐month 5xFAD mouse model was employed in the comparison to the Kat5 cKO dataset. The transcriptomic datasets for the 5xFAD mouse are available on the AD Knowledge Portal (https://adknowledgeportal.synapse.org; synapse ID: syn66318332).

### 2.6 Human FTD dataset

We examined the transcriptomic data emerging from the Risk and modifying factors in Frontotemporal Dementia (RiMod‐FTD) consortium,^36^ specifically comparing the differential expression in the case‐control study of FTD patients with MAPT mutations to the Kat5 KO mouse model differential expression profile to assess AD specificity of the Kat5 molecular signature alignment.

### 2.7 Datasets for neurodegenerative disease comparisons

The comparison of the different neurodegenerative mouse models borrows from previous work by Wan et al.^37^ characterizing co‐expression modules and clusters across human and murine datasets. The PD dataset was drawn from GEO (GSE205450), which contained 81 cases and 69 controls^38^ in which RNA‐seq molecular profiling was performed from the caudate and putamen. The Huntington's disease (HD) dataset was downloaded from GEO (GSE64810) in which transcriptomic analysis was performed with samples from the prefrontal cortex of 49 cases and 20 controls.^39^ The exploration of ALS employed the NYGC ALS dataset in GEO (GSE137810), containing 114 cases and 16 controls.^40^

### 2.8 Differential expression analysis of the explored neurodegenerative models

Differential gene expression (DGE) analysis was performed separately for each disease cohort using raw count data and a standardized DESeq2 pipeline, with disease status modeled while adjusting for dataset‐specific biological covariates (e.g., age, sex, and *post mortem* interval) and technical covariates (e.g., RNA integrity metrics, sequencing batch effects, tissue, or brain region) based on available metadata. Log_2_ fold‐change shrinkage was applied using the apelgm method to reduce the variability of effect sizes for genes with low counts. Batch‐adjusted expression values were generated using the limma removeBatchEffect function and visualized by UMAP to assess sample clustering patterns by disease status.

### 2.9 Gene set enrichment analysis (GSEA) analysis of the biological domains

#### 2.9.1 Biological domain enrichment

All datasets were aligned to the TREAT‐AD biological domains employing GSEA to the annotated Gene Ontology (GO) terms across the three tiers of GO. The enriched GO terms were organized into the biological domains based on the definition profiles of the AD biological domains,^30^ and the biological domains were ranked based on the frequency of enriched term participation. To evaluate the comparative enrichment of various biological domains and their respective GO terms, GSEA was conducted utilizing the gseGO function within the clusterProfiler R package.^41^ Subsequently, the outcomes were grouped into biological domains according to the enriched terms' GO ID. Each enrichment analysis was conducted using non‐zero log fold‐change values, sorted in descending order. The presented outcomes comprise the normalized enrichment score (NES) and the Benjamini‐Hochberg corrected *p* value (*p*_adj_) for GO terms associated with each biological domain.

### 2.10 Shared biological domain GO term enrichment between models

All pairwise comparisons made between the Kat5 cKO and the AD datasets were enriched for the GO terms mapping onto the 19 biological domains. The GO terms shared between the groups were mapped per biological domain, and the domains with shared patterns of enrichment were identified and presented. The enrichment draws from the transcriptomic datasets for each model and accordingly contains directional information about change in regulation. Only domains with at least five terms of coordinate regulation are considered significantly overlapping and surfaced in the present work.

### 2.11 Correlation analysis between datasets

The gene correlation analysis between the Kat5 cKO differentially expressed genes (DEG) and those differentially expressed in AD models employ Pearson correlation performed by R stat package in BioConductor. The Pearson *r* correlation term shows the overlap between the whole DEG set models, without stratification into biological domains or GO terms. The *p* value calculation is performed to assess the statistical relevance of the associated correlation (*r*) value, and the beta coefficient index provides the interaction slope of the compared models.

### 2.12 GSEA and correlation analysis using the AD subdomains

We previously described a structured framework of biological domains and corresponding subdomains (Synapse: syn25428992) capturing AD‐relevant endophenotypes by integrating information across genetic association studies, predicted variant impact, and linkage with dementia. These data were used to annotate the GO terms obtained from clusterProfiler.

### 2.13 Correlation analysis with Kat5 cKO mouse model

DGE results were aggregated across all disease cohorts, and GSEA was performed for each disease. KAT5‐associated differential expression results were obtained from Urban et al.^23^ and mouse–human orthologs were mapped using the g:Orth feature from gProfiler2 package. GSEA was subsequently performed on the corresponding human ortholog gene sets. Correlation analyses were conducted across diseases by comparing NESs and statistical significance (*p*_adj_ < 0.05) across disease‐specific and KAT5‐associated enrichment results.

### 2.14 Transcriptome‐wide enrichment correlation analysis

This analysis was conducted using genome‐wide ranked gene lists generated from limma‐voom linear modeling of normalized RNA‐seq count data. Genes were ranked based on eBayes t‐statistics for case/control status while adjusting for relevant biological and technical covariates. GSEA was performed on the full transcriptome without thresholding for differential expression metrics. NESs were subsequently used to compute correlations across disease cohorts and KAT5‐associated enrichment profiles.

### 2.15 Kat5 cKO DEG Kappa‐network modeling

**FIGURE 1:**
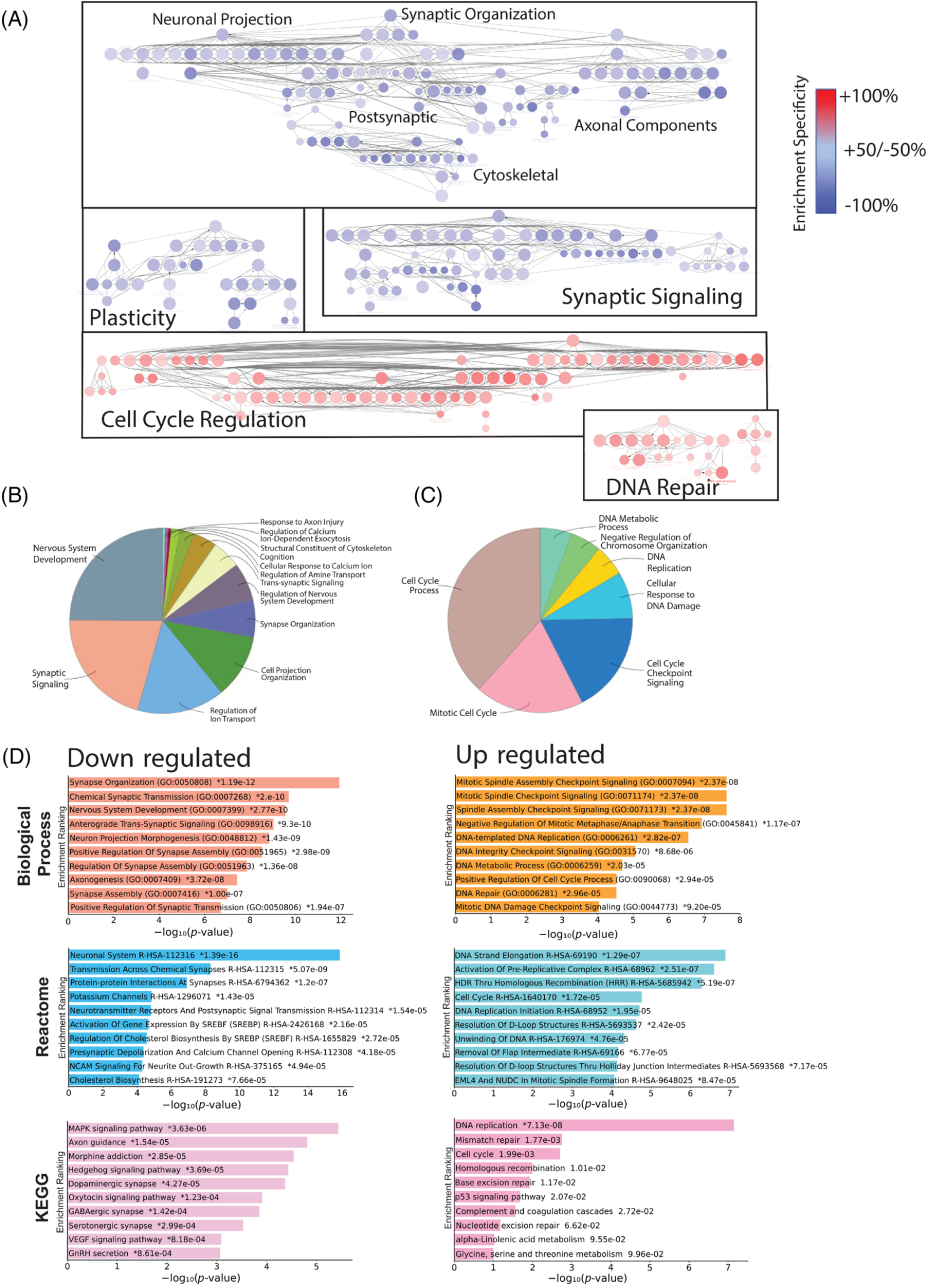
Up‐ and downregulated processes in KAT5 cKO. (A) Kappa‐networks were constructed from the up‐ and downregulated gene sets derived from the KAT5 cKO mouse CA1 hippocampus. The nodes represent Gene Ontology (GO) biological process terms, and the linking edges represent the kappa‐score derived from shared genes annotated between GO terms. The annotation of the subclusters of the network represent the broader biological families of impacted biological processes. The blue nodes are downregulated and are predominantly related to synapse‐linked processes, with development, plasticity, and synaptic signaling being the three largest families. Upregulated processes are shown in red and are predominantly centered upon DNA metabolic and cell cycle functions. (B) All the downregulated processes are represented by a pie chart showing the major themes and fractional composition of the GO term annotation, in which synaptic development and function are the predominant fractions. (C) The upregulated terms are represented by fractional contribution of enrichment, demonstrating specific classes of DNA metabolic and cell cycle events playing the largest role. The top 10 terms from each branch of GO biological process (D, top), KEGG pathway analysis (D, middle), and Reactome pathway mapping (D, bottom) are shown, with the downregulated processes on the left and the upregulated processes on the right.

The Kappa‐network approach identifies GO biological process (BP) enrichments based on the DEGs. These networks form an exhaustive set of related processes, linked by genes annotated to multiple GO terms, and the degree of term conservation is the linking edge between nodes. These are mapped based on their derivation from either up‐ or downregulated DEGs, so process families can be explored simultaneously based upon^1^ significance level,^2^ degree of conservation of genes between terms (kappa‐value), and^3^ the directionality of DEG shift. The network modeling was performed using the cytoscape application Clue‐GO with a kappa value set at 0.5 and a selectivity for specific directionality of 0.6 for inclusion into either the downward or upward directed network. The grouping of terms observed in Figure 1 was based on the best term characterization of the boxed subcomponent of the network observed in either regulation direction. The calculation of the total fraction of top‐level terms was tallied and grouped based on the most enriched term being led with ranking elevation given to the highest ontological position within GO in the cluster.

### 2.16 Characterization of enrichment of Kat5 DEGs

The enrichment of terms within each branch of the GO employed the R based web‐accessible tool Enrichr (https://maayanlab.cloud/Enrichr/).^42^ Here the top 10 terms within the biological process branch of the GO were mapped for both the upward and downward regulated gene sets to provide a direct comparison to the Kappa‐network enrichment. Similarly, the top 10 associated Kyoto Encyclopedia of Genes and Genomes (KEGG) and Reactome pathways were also demonstrated for the up‐ and downregulated gene sets. The upregulated and downregulated DEGs within the Kat5 cKO model at each tier (1.2, 1.5, and 2‐fold) were compared to the 2023 GWAS Catalogue (which incorporates both TopMed and UK Biobanks data)^43^ to assess enrichment of genetic trait linkage using the Enrichr tool.^44^

### 2.17 iPSC neuronal models employed

#### 2.17.1 Cell culture

Human induced pluripotent stem cells (hiPSCs) that were either control/wild type (WT) or harboring the heterozygous APP Swedish (SWE APP) (KM670/671NL), heterozygous PSEN2 (N141I) mutation, or an APP duplication were differentiated into glutamatergic excitatory cortical neurons using dual‐SMAD inhibition, and neuronal populations were further enriched using magnetic bead selection by depleting CD184‐, CD44‐, and CD271‐positive cells as previously described.^45^, ^46^, ^47^ Control hiPSCs were derived from a non‐demented subject and are isogenic to cells carrying SWE APP and PSEN2 mutations. Following neuronal purification, cells were replated on Geltrex‐coated coverslips for 5 days prior to immunocytochemistry. SWE APP hiPSCs and APP duplication hiPSCs have been characterized and published.^48^, ^49^ The PSEN2 N141 variant was introduced into control cells using CRISPRRP‐Cas9 genome engineering following established protocols and screened for the absence of major chromosomal abnormalities (07550, Stemcell).^46^ hiPSCs were tested for mycoplasma using MycoScope Mycoplasma PCR Detection Kit (Amsbio, Catalog No.: AMS.MY01100) according to the manufacturer's guidelines. Karyotyping was performed using hPSC Genetic Analysis Kit (StemCell, Catalog No.: 07550) per manufacturer guidelines.

#### 2.17.2 hiPSC immunohistochemistry

hiPSC colonies were fixed with 4% paraformaldehyde (Thermo Fisher Scientific, Catalog No.: 28908), permeabilized and blocked, and probed with primary antibodies Nanog (Cell Signaling Technology, Catalog No.: 4903) at 1:400 and OCT3/4 (Santa Cruz Biotechnology, Catalog No.: sc‐5279) at 1:100. Alexa Fluor secondary antibodies used were Alexa Fluor 594 goat anti‐rabbit IgG (Thermo Fisher Scientific, Catalog No.: A11012) at 1:2000 and Alexa Fluor 488 goat anti‐mouse IgG (Thermo Fisher Scientific, Catalog No.: A11029) at 1:2000, respectively. Mounting medium was Prolong Gold antifade reagent with DAPI (Invitrogen, Catalog No.: P36931). hiPSC colony imaging was performed on a Leica TCS SP8 confocal laser microscope with a 20× lens.

#### 2.17.3 Neuron immunocytochemistry

Cells were fixed with 4% paraformaldehyde, and following fixation, permeabilization, and blocking, neurons were treated overnight with primary antibodies targeting TIP60/KAT5 (Invitrogen, Catalog No.: PA5‐34548) and MAP2 (Abcam, Catalog No.: ab92434) at 1:500 and 1:3000 dilutions, respectively. After washing, cells were treated with secondary antibodies: goat anti‐rabbit Alexa Fluor 488 for TIP60 and goat anti‐chicken Alexa Fluor 594 for MAP2, both at 1:1000 dilution (Thermo Fisher Scientific).

#### 2.17.4 KAT5 immunofluorescent imaging and analysis in hiPSC‐derived neurons

Imaging was performed on a Leica TCS SP8 confocal laser microscope with a 20× lens. Images were acquired in the Leica Application Suite X (LAS X 3.5.519976) with the following settings: 405 nm laser at 1% and HyD detector 1 gain at 100%; 488 nm laser at 1% and photomultiplier tube detector gain 1247 V; 552 laser at 1% and HyD detector 2 gain at 100%. Image format was 1024 × 1024, speed 400 Hz, and 7× zoom. Image analysis was performed on 10 to 125 neurons per genotype using Bitplane Imaris software (Oxford Instruments). Neuronal nuclei were identified by the presence of MAP2 (red) cytoplasmic staining. A surface object workflow was used in Imaris to define the DAPI+ nuclear region of interest (ROI). Neuronal nuclei were identified by the absence of MAP2 cytoplasmic staining. The nuclear ROI surface was used to mask the KAT5 (green) channel and isolate nuclear KAT5 staining. A surface object workflow was run on the masked KAT5 channel to quantify nuclear KAT5 fluorescent intensity in the neurons.

A one‐way ANOVA and Holm‐Šídák's multiple‐comparisons test were used to compare nuclear KAT5 fluorescent intensities between the FAD hiPSC‐derived neurons and WT neurons.

#### 2.17.5 Second site validation experiments of Kat5 localization

To independently validate the Kat5 localization results in FAD hiPSC‐derived neuronal lines, a second site performed similar experiments with different hiPSC cell lines, and an additional Kat5 antibody was employed that underwent knockdown validation studies by the manufacturer.

#### 2.17.6 Second site hiPSC culture and differentiation protocol

iPSCs were differentiated into neural progenitor cells (NPCs) using STEMDiff Neural Induction Medium (NIM). iPSCs were placed into a single cell suspension in NIM with SMADi/ROCKi (SMAD inhibitor and ROCK Inhibitor) in an AggreWell 800 plate. Embryoid bodies were cultured in the AggreWell plate for 5 days with NIM/SMADi partial medium changes daily. Embryoid bodies were then plated onto Matrigel‐coated plates and fed daily with NIM/SMADi medium until day 12 to allow neural rosette formation. Neural rosettes were selected using Neural Rosette Selection Reagent (StemCell Tech) and plated onto Matrigel‐coated dishes with NIM/SMADi. Medium was changed daily for 7 days, after which NPCs were cryopreserved and split into defined neural progenitor medium (StemCell Tech). NPCs were plated onto PLO/Laminin‐coated dishes in neural progenitor medium. The following day a half medium change was carried out with defined Brain Phys Medium (with N2A, SM1, BDNF, GDNF, cAMP, and ascorbic acid). Half medium changes occurred every other day for 7 days.

#### 2.17.7 hiPSC lines employed at second site

Five cell lines were employed in the validation studies: one male (UCSD109i‐2‐8) and one female (UCSD079i‐1‐12) non‐disease control line, a male (HVRDi001‐A‐1) and a female (HVRDi002‐A) line from the APPV717I FAD mutant, and one female FAD APP‐duplication (UCSD241i‐APP2‐3) were employed. All cell lines were purchased from WiCell.

#### 2.17.8 Immunohistochemistry

iPSC‐derived neurons were fixed with 4% paraformaldehyde in phosphate‐buffered saline (PBS) for 1 h at room temperature. Cells were then permeabilized and blocked for 1 h at room temperature using 0.1% Triton‐X‐100 and 1% bovine serum albumin in PBS. Primary antibodies were incubated overnight at 4°C at 1:200 in PBS; TUBB3‐specific/TUJ1 Monoclonal antibody (ProteinTech Catalog No.: 66375‐1‐Ig), TIP60/KAT5 polyclonal antibody (ProteinTech, Catalog No.: 10827‐1‐AP), or TIP60 polyclonal antibody (Thermo Fisher Scientific, Catalog No.: PA5‐34548). Cells were washed three times with PBS and incubated with secondary antibodies at 1:500 for 1 h at room temperature (Thermo Fisher Scientific, Catalog No. A‐31572 and A‐11001) and Hoechst dye. Cells were washed three times with PBS.

#### 2.17.9 Cell imaging and analysis

Cells were imaged using a Cytation 1 from BioTek at 20×, using automated imaging settings applied evenly across all samples. The Cytation 1 instrument calculated nuclear Tip60 intensity and nuclear Tip60 puncta through automated image analysis. All data were normalized to cell number.

## 3 RESULTS

We assessed whether the disruption to KAT5 nuclear chromatin regulation was a driving component of AD pathogenesis by comparing the Kat5 cKO transcriptomic signature(23) to murine and human AD datasets. First, we explored the enrichment signatures of the Kat5 cKO mouse using complementary techniques of Kappa‐network models and GSEA. The Kappa‐network methodology is employed to capture the greatest breadth of altered processes within the KAT5 model, while the GSEA approach focuses on GO term‐ and pathway‐specific enrichment patterns. The enrichment of the downregulated gene set highlighted predominantly synaptic genes (Figure 1A, blue, and B) in keeping with the original characterization.^23^ The synaptic gene downregulation network clusters into three subnetworks that align with synaptic developmental, plasticity, and synaptic signaling. The upregulated kappa‐network cluster into cell cycle and DNA repair subnetworks (Figure 1A, red, and C).

The enrichment of Kat5 DEGs shows downregulated processes and pathways in the left column and upregulated on the right (Figure 1D). The top 10 downregulated GO BPs are all synaptic, split between structural, developmental, and signaling‐related terms, while upregulated BPs are cell cycle and DNA replication and repair focused, consistent with the appa‐network analysis. These results are mirrored in both the Reactome and KEGG pathway enrichments (second and third rows, Figure 1D), with Reactome highlighting neuronal signaling and cholesterol biogenesis and KEGG representing specific neurotransmitter and endocrine signaling processes within the downward DEGs. Interestingly, KEGG pathway enrichment identifies GABAergic synapse downregulation, consistent with recent findings suggesting that loss of GABAergic neurons occurs earlier in the disease sequelae.^50^ Conversely, the upregulated DEGs enrich cell cycle and DNA replication and repair GO biological processes, while KEGG pathway enrichment highlights complement and coagulation cascades, pointing to innate immune processes involved in synaptophagy (Figure 1D, right side, second and third rows).

**FIGURE 2:**
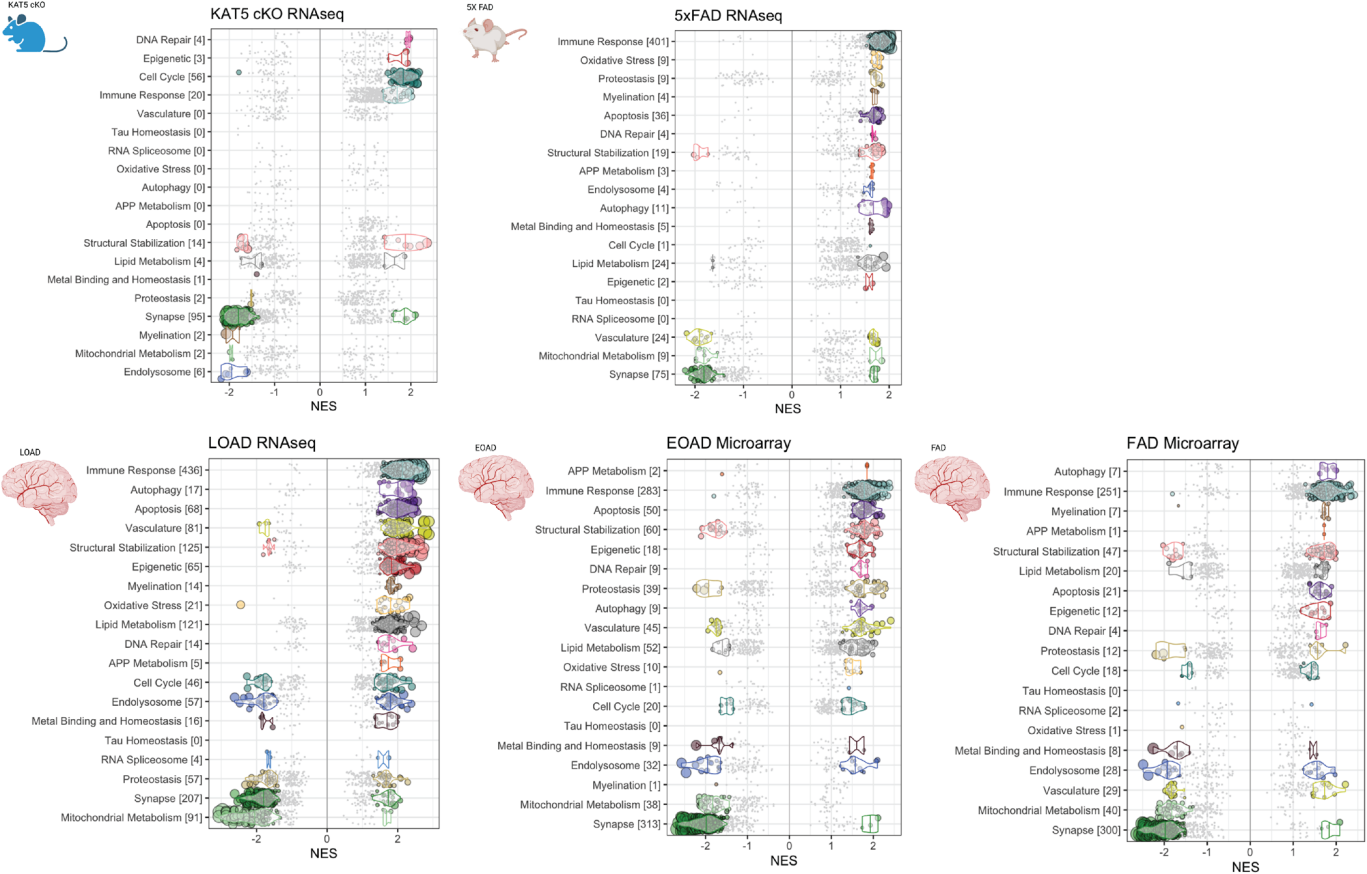
Biological domain comparisons of all AD datasets. All datasets, including KAT5, the 5xFAD murine model, human LOAD transcriptome, EOAD, and FAD human datasets are broadcast onto the TREAT‐AD biological domains based upon GSEA enrichment of GO terms within the DEGs of each transcriptome. The murine models are on the top, denoted by mouse symbols, while the three human datasets are on the bottom: LOAD (left), EOAD (middle), and FAD (right). The negative normalized enrichment score (NES) denotes downregulated GO terms, while positive NES means the leading‐edge genes associated with that term are upregulated.

The TREAT‐AD biological domains map each dataset independently, both murine and human, into the 19 discrete endophenotypic areas (Figure 2). The Kat5 model (upper left) upregulates cell cycle, immune response, and structural stabilization (a domain reflecting cell–cell junctions, connections to cytoskeletal elements, and extracellular matrix [ECM] factors^30^), while downregulating synapse, endolysosome, myelination, and mitochondrial metabolism (Figure 2). The murine 5xFAD model also upregulates immune response and downregulates synapse (upper right, Figure 2). The human datasets represent three distinct AD subtypes: LOAD, EOAD (non‐genetic), and FAD (PSEN1 mutation). Each subtype demonstrates upregulated immune response, cell cycle, DNA repair, and structural stabilization, concomitantly with downregulated synapse, mitochondrial metabolism, and endolysosome, all shared with the Kat5 AD biological domain profile. A set of biological domain‐specific GO terms are enriched across all datasets, which includes the positive enrichment of complement activation and negative enrichment of synaptic vesicle recycling and post‐synaptic density.

**FIGURE 3:**
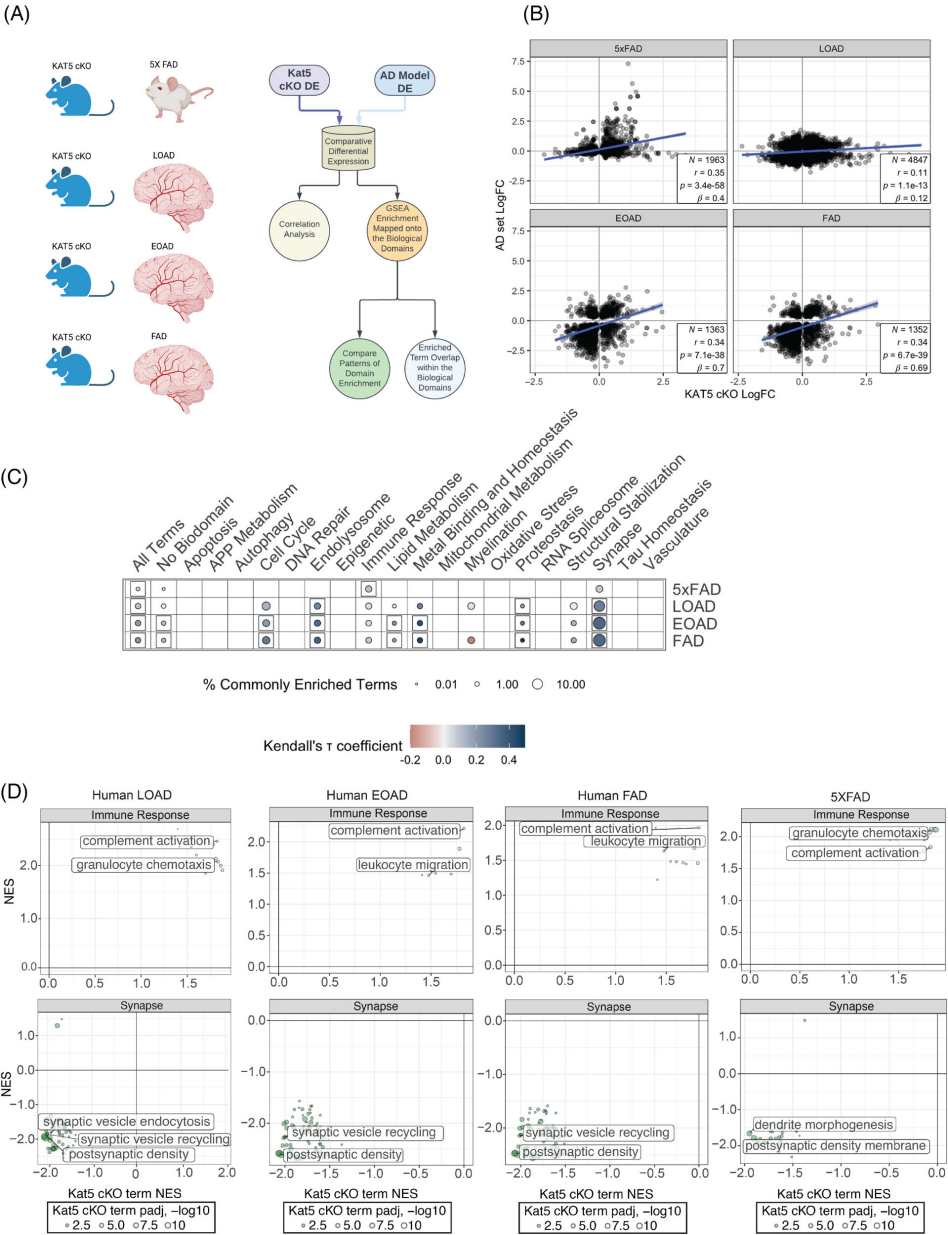
KAT5 correlations with AD datasets. To test the similarity of the differential expression pattern observed within the KAT5 cKO to extant AD datasets, we performed a set of comparisons to murine and human AD subtypes, with the diagrammatic process depicted in (A), the Pearson correlations shown in (B), and the correlation between each dataset with KAT5 DEGs mapped onto the TREAT‐AD biological domains (C). The binary correlations between KAT5 and each AD subtype or model are provided for the most consistently up‐ and downregulated biological domains, immune response, and synapse, respectively (D). Note: (A) made with BioRender.com.

The methodology employed for Kat5 and AD dataset comparisons is shown diagrammatically in Figure 3A, with the pairwise analysis shown on the left and the methodology employed on the right. The Pearson correlation (*r*) associated with each binary model comparison demonstrates a relatively weak correlation overall (ranging between 0.11 for LOAD and 0.35 for 5xFAD) but highly statistically significant (Figure 3B), suggesting overlap within a portion of the DEGs. We examine the biological areas of elevated correlation within the Kat5 and AD comparisons apparent in AD biological domain correlation analysis. The AD biological domains are a TREAT‐AD resource developed to subdivide gene sets into specific biological endophenotype‐linked processes.^30^ Consequently, comparing the correlation within specific AD biological domains should show the AD‐linked biology with the highest correlation between datasets. Across all examined murine and human datasets, synapse and immune response are the most consistently and positively correlated biological domains (Figure 3C). There is significant correlation between Kat5 and all human datasets within the endolysosome and proteostasis domains.

The Kat5 cKO comparison to the LOAD DEG dataset is critical to understanding whether Kat5 plays a role in AD pathogenesis and, if so, within which biological processes or pathways. To examine this issue, we intersected the downregulated genes from LOAD and Kat5 cKO and used the resulting 845 shared downregulated gene set to seed GO and pathway enrichment analyses (Figure S1, top, Venn diagram). The GO enrichment of the common downward DEGs highlights synaptic processes, including pre‐synaptic and post‐synaptic processes and pathways (Figure S1, bottom panels). There is notable enrichment of GABAergic signaling (Figure S1: KEGG, WikiPathways, GO cellular component), suggesting a shared impairment in inhibitory synapses within LOAD and the Kat5 cKO model. Neurodevelopmental processes are represented within the enrichment analyses, such as axonal projection or neurodevelopmental process (Figure S1: GO BP CC, Reactome), which is consistent with the Kappa‐network enrichment of the KAT5 model (Figure 1). Metabolic pathway downregulation of glycolysis and lipid metabolism (Figure S1: WikiPathways, MSIGDB) is common to LOAD and Kat5 cKO, further supporting metabolic impairment as a common biological process disrupted across datasets. Finally, LOAD and Kat5 cKO data share signatures with the GTEX genes downregulated in brain between young subjects and older individuals, arguing for LOAD and Kat5 cKO convergence on aging brain genes (Figure S1, bottom right).

The 599 upregulated DEGs intersecting LOAD and Kat5 cKO were used to conduct seed enrichment analysis of these two datasets (Figure S2, Venn diagram). GSEA of the common DEGs demonstrates GO term and pathway enrichment of immune function and DNA replication and repair. The common immune genes are involved in proinflammatory cytokines, such as tumor necrosis factor (TNF) and members of the interleukin family, as well as phagocytic and complement pathways (Figure S2: KEGG, WikiPathways). GTEX signatures show brain aging genes are upregulated (Figure S2), which is the converse of the downregulation of GTEX brain aging signatures in downregulated DEGs. We examined the gene overlap within the discordantly regulated gene sets to determine whether there are processes that were markedly opposed between LOAD and the Kat5 cKO, which pointed to transcriptional or chromatin regulation within Kat5 Down‐LOAD Up gene set and DNA repair or cell cycle processes within the Kat5 Up‐LOAD Down gene sets (Figure S3).

We performed supplementary analysis of the downregulated gene intersection between LOAD and Kat5 DEGs in SynGO, a portion of the GO focused on synaptic structure and process. This identified pre‐ and post‐synaptic processes (Figure S4), with similar degrees of representation, showing dendritic and axonal processes in the overlapping Kat5 and LOAD biology. Consistent with the biological domain analysis, the metabolic profile of the overlapping downregulated genes implicates GABA, as well as both forms of nicotine adenosine dinucleotide (NAD and NADH) (Figure S4, Human Metabolome Database). These metabolic data support Kat5 and LOAD dysregulation of GABA‐related signaling and the tricarboxylic acid (TCA) cycle, which fuels the electron transport chain (ETC), further supporting the decrement in synaptic and mitochondrial function as common biological impairments.

Examining specific gene expression changes in Kat5 cKO, we observed downregulation of neuronal glucose transport (Glut3/Slc2a3) and glycolytic metabolism of glucose into pyruvate (Figure S5A). Mitochondrial genes involved in pyruvate import into mitochondria and TCA cycle function are also downregulated (Figure S5B), along with key electron transport chain genes (Figure S5C). These observations further demonstrate mitochondrial impairments in the Kat5 cKO mouse model. We explored whether upregulation of immune response in Kat5 cKO might impinge upon microglial state regulatory genes. These analyses show small changes within homeostatic marker genes in microglia, particularly P2ry12 (Figure S6A), and upregulation of many activation markers, including Syk, TyroBP, Axl, and Trem2 (Figure S6B). The Kat5 gene deletion occurs only in neurons, which argues for a non‐cell‐autonomous effect of neuronal deficits on microglial activation.

**FIGURE 4:**
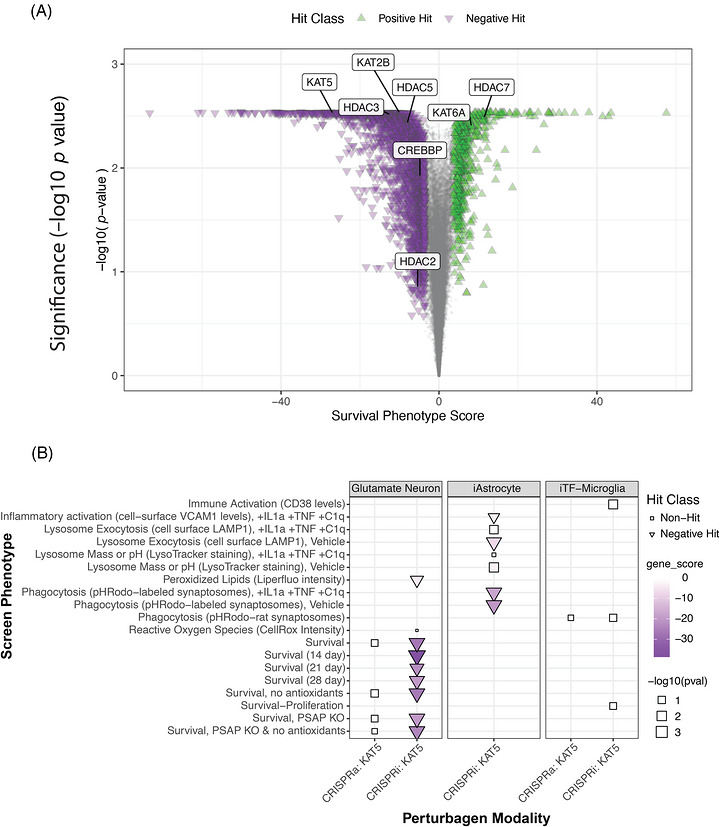
KAT5 and other epigenetic regulatory factors involved in neuronal survival in CRISPRbrain studies. CRISPRbrain performs functional genomics studies in iPSC‐derived brain lineages in association with focused assays examining traits of relevance for human health such as neuronal survival. The CRISPRbrain genetic models employ both CRISPRi and CRISPRa approaches to assess the impact of up‐ or downregulation of a specific gene upon cellular physiology. (A) The neuronal survival assay is demonstrated by volcano plot using survival assay phenotype score along the abscissa and the probability along the ordinate axes. KAT5 is the most significantly ranked epigenetic regulator, the deletion of which has negative impacts upon the neuronal survival score. Note that other epigenetic regulators with shared and opposing functions are also linked with neuronal survival. (B) Plots of collective set of simple assays performed with KAT5 CRISPRi model in different cellular lineages, with intensity of color denoting the gene score associated with the assay, the size of the icon denoting statistical significance, the triangle shape associated with a negative hit, and a square shape denoting a non‐hit. All neuronal survival assays performed across different timeframes show KAT5 deletion negatively and significantly associated with survival. Phagocytotic astrocytes are also negatively associated with KAT5 function. There are no significant microglial assay results for KAT5.

To assess the role of KAT5 in the central nervous system (CNS), outside of a disease context, we utilized the CRISPRbrain resource.^51^ CRISPRbrain is a functional genomics platform out of the University of California, San Francisco that employs hiPSCs differentiated into neurons, astrocytes, and microglia to interrogate specific gene interactions with core CNS functions across core areas of physiological function, including survival, metabolic tone, reactive oxygen species production, phagocytosis, and immune activation, among others. We specifically looked at the CRISPRi knockdown of KAT5 to determine the impacts observed in human neurons in the absence of any disease state. If KAT5 is a driving contributor to AD pathophysiology, we would expect that human neurons with decreased KAT5 would replicate some facets of AD pathology. KAT5 inhibition consistently decreased neuronal survival and was the most significant epigenetic regulator for neuronal survival (Figure 4A, purple = impaired survival; green = promoted survival). Interestingly, within histone acetylases and deacetylases, gene deletion of either class has a predominantly negative impact on neuronal survival, suggesting a complex role for epigenetic regulation in neuronal survival outcomes. Loss of KAT5 or CREBBP, both major brain histone acetylases, diminishes neuronal survival (Figure 4A), consistent with a neuronal homeostatic role promoting survival.

Panning across CRISPRbrain assays, we observe a uniformly consistent decrement of neuronal survival in KAT5 knockdown neurons (Figure 4B, left box, CRISPRi), with little effect of KAT5 activation (Figure 4B, CRISPRa). In astrocytes, however, inhibition of KAT5 produced notable deficits in lysosome exocytosis, inflammatory activation in response to cytokine (TNF/IL‐1a), and complement (C1q) treatment, as well as significant impairment in synaptic phagocytosis (Figure 4, middle box). These observations support a role for KAT5 in multiple cell‐type‐specific processes involved in the AD pathogenic sequelae.

To explore genetic support for the transcriptomic profiles of Kat5 cKO, genetic variant trait linkage was performed with the up‐ and down‐regulated genes using data from the 2023 GWAS catalog, which contains over 400,000 single‐nucleotide polymorphism‐trait associations across more than 5000 associated traits.^43^ The aim of this exploration was to assess whether the observed DEGs in the Kat5 cKO model were already genetically linked to AD‐associated traits, suggesting human AD relevance to the specific genes demonstrating altered regulation. We do not show that the trait‐variant relationship mapped to these genes aligns with the expression pattern observed, but the analysis provides some genetic linkage to the potential significance associated with the gene and the human disease state. The analysis was performed in a stepped manner, in which we panned across the downregulated gene set based on the degree of differential expression. The goal was to balance potential issues of sensitivity of enrichment, which may be diluted within the lower‐level DEGs, versus truncating implicated processes by setting too stringent a standard based on high fold enrichment. Within the downregulated gene set, the primary enrichment employs the 2792 DEGs that are 1.2‐fold downregulated (most permissive) in the Kat5 cKO model. When we performed the trait enrichment, the top terms implied linkage with anatomical development, cognitive and psychiatric health (biopolar disorder, highest math score), and direct trait linkage with AD (“Alzheimer's Disease [Cognitive Decline],” “Psychosis and Alzheimer's Disease”) (Figure S7A). Increasing the stringency to 1.5‐fold DEGs (which number 949), we observe the top two enriched terms are “Psychosis and Alzheimer's Disease” and “Lateral Ventricle Volume,” a known physiological biomarker of limbic and temporal lobe shrinkage associated with neurodegeneration (Figure S7B). At 2‐fold DEG, the highest stringency tested, we had 168 genes trait‐linked with “diabetes medication use,” “central cerebrospinal fluid amyloid Aβ42 to Aβ40 ratio,” and “lateral ventricle horn volume,” all possessing direct or indirect associations with AD pathological progression.^1^, ^52^ A similar analysis was performed with the upregulated genes with no compelling findings (Figure S8). Consequently, the downregulated Kat5 cKO neuronal genes were genetically associated with human traits directly connected to AD, making a strong case for a direct role for KAT5 in AD pathological progression.

The Kat5 cKO overlap in molecular profile with FTD patient transcriptomic data from the RiMOD consortium (see methods) was explored to assess the AD specificity of the similarity. The overlap between Kat5 cKO and the FTD patients was considerably smaller than with AD (Figure S9), with less than 10% overlapping genes in the up‐ or downregulated gene set. Not surprisingly, there are some similarities in biological enrichment, likely due to common molecular pathways involved in neurodegenerative events, also observed in recent explorations of the molecular profiles across neurodegenerative diseases.^53^, ^54^

**FIGURE 5:**
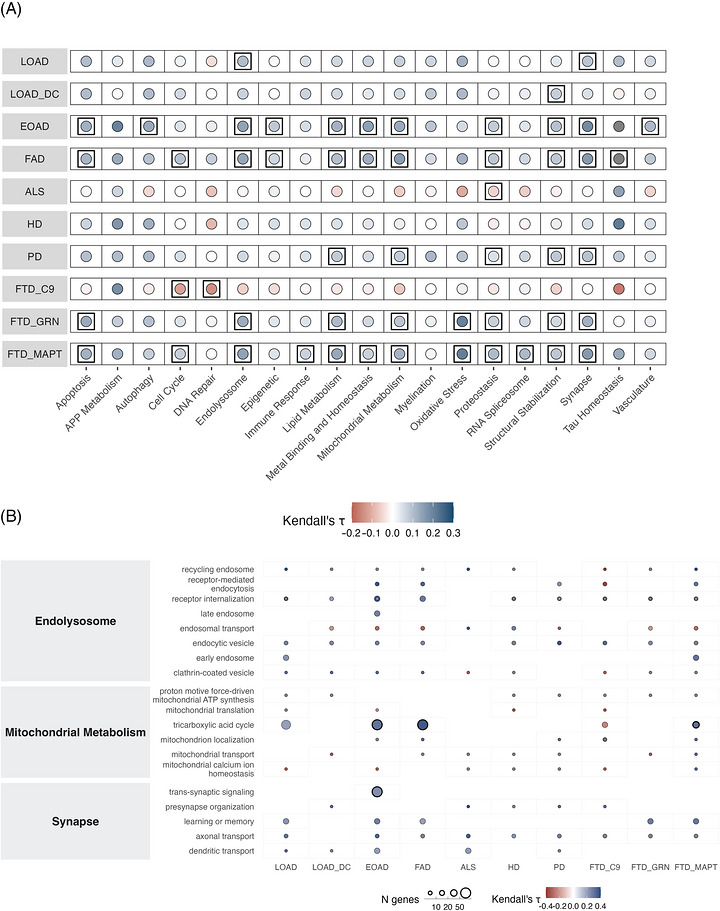
Transcriptome correlation between human neurodegenerative disease and Kat5 cKO. We compared the biodomain (A) and subdomain (B) correlations between LOAD, LOAD diversity cohort (LOAD_DC), EOAD, FAD, ALS, HD, FTD_C9, FTD_GRN, FTD_MAPT, and PD to Kat5. The biodomains are arrayed along the bottom horizontal axis in (A) and along the left vertical axis in (B). The degree of correlation is measured by Kendall's tau (Τ), with negative associations shown in red and positive associations in blue. The size of the dot plot represents the Jaccard index, a measure of the overlap between the two gene sets being compared. The box around the dot plot demonstrates a statistically significant positive or negative correlation following correction.

To explore the association between Kat5 cKO dysregulated genes and human neurodegenerative disease, we examined the transcriptional correlation between Kat5 cKO and representative datasets from ALS, HD, TREAT‐AD LOAD, the LOAD diversity cohort (LOAD_DC), EOAD, FAD, and PD. The correlation analysis of the transcriptomic data between case and controls was compared to the Kat5 cKO in the AD biological domains (Figure 5A) and the subdomains (Figure 5B). We observed a significant positive correlation across EOAD and FAD, as indicated by the boxes around the dot plot (Figure 5A, rows 3 and 4). A significant positive correlation is observed in LOAD within endolysosome and synapse biological domains (Figure 5A). There is no significant positive correlation observed with ALS or PD; however, a significant negative correlation was observed in proteostasis in ALS. PD demonstrates a strong positive correlation in multiple domains, including lipid metabolism, mitochondrial metabolism, proteostasis, structural stabilization, and synapse. Within FTD, we observed divergent patterns of overlap depending on the FTD subtype. FTD_C9 showed no significant positive correlation and weak to significant negative correlation across most biodomains. FTD_GRN and FTD_MAPT, in contrast, demonstrated high levels of positive correlation across the majority of biodomains. We only showed the subdomain analysis for three biodomains for clarity: endolysosome, mitochondrial metabolism, and synapse. Intriguingly, we observed the strongest signal in the AD datasets within mitochondrial metabolism – specifically within the TCA cycle. As the TCA cycle byproducts feed into epigenetic regulation, this suggests a potent AD‐predominant association with the Kat5 cKO in elements of metabolism.

We further explored the AD specificity of the Kat5 cKO association with neurodegenerative processes using the large set of mouse models utilized to define the commonly employed AD consensus clusters.^37^ This seminal work by Wan et al. draws together mouse models representing different genetic drivers in AD, Creutzfeldt‐Jakob disease (CJD), FTD, ALS, HD, and spinocerebellar ataxia (SCA). We performed GSEA‐based biodomain analysis across disease states looking at the degree of correlation with the enriched GO terms observed in the Kat5 cKO and found initially a high degree of similarity across disease states within the synapse biodomain (Figure S10). The broad GSEA enrichment also demonstrated a high degree of similarity between the Kat5 cKO and the FTD‐ALS models. The CJD, HD, PD, and SCA models showed more variable correlation outside of the synapse biodomain, which appeared from this analysis to be common between Kat5 cKO and most neurodegenerative diseases.

We speculated that perhaps synaptically impacted processes were conserved across neurodegenerative diseases, but the specific genes altered across disease states may diverge. To test this idea, we performed transcriptome‐wide correlation analysis across models to assess whether the shared domain‐level association would be maintained at the gene level (Figure S11). Consistent with our hypothesis, we observed a stronger correlation between Kat5 cKO and the AD models (including tau and p25/cdk5 models) in the gene‐centric correlation analysis, and we observed a weaker correlation in the other neurodegenerative disease mouse models. While there was a strong correlation between specific neurodegenerative mouse models of FTD‐ALS, CJD, and SCA, the results were far less consistent outside of the AD models. These results support a model in which some biological processes may be common to neurodegenerative disease, but the details of the molecular mechanisms differ significantly.

To examine this possibility with greater biological process resolution, we did a similar pairing of analytical approaches within synapse (most conserved from the GSEA biodomain analysis, Figure S10) and immune response (a less well‐conserved biodomain across disease models) subdomains. Similar to what we observed in the GSEA‐based correlations in the biodomains, we observe that many of the synapse subdomains appear common across disease models, especially post‐synaptic organization, “other,” pre‐synaptic organization, and synaptic vesicle cycle (Figure S12). To better understand whether this was a biological process or gene‐level effect, we performed transcriptome‐wide correlation analysis within the synapse subdomains (Figure S13). Similar to the parent biodomains, we found that gene‐centric correlation analysis strengthened the Kat5 cKO association in the AD mouse models but weakened the relationship in other neurodegenerative mouse models. Some individual non‐AD mouse models still have a high correlation with the gene‐centered analysis, including several within FTD‐ALS, but it is less consistent across disease models generally. In the AD mice, APP and tau models showed a strong correlation with the Kat5 cKO transcriptome.

The immune response biodomain appeared to have greater AD specificity at the domain level, so we compared the GSEA GO term‐based analysis to the gene‐centric transcriptome correlation analysis across immune subdomains. The GSEA‐based analysis highlighted focal subdomains in which AD models shared GO‐term process enrichment with the Kat5 cKO, in which a statistically significant positive correlation was constrained to leukocyte chemotaxis and “other” (a set of IR GO terms not mapped into any subdomain) (Figure S14). These subdomains were significant across almost all AD mouse models. While there was a significant correlation between other disease state models and the Kat5 cKO, the correlations were sporadic and not consistent in direction, as positive and negative correlations were seen in CJD, FTD‐ALS, and HD. SCA mostly closely resembled the correlations observed in AD. Transcriptome‐wide correlation analysis shifted the results in two ways. First, there was a broader significant correlation between the AD mouse models and Kat5 cKO across all immune subdomains. Second, the signal in other neurodegenerative disease state mouse models expanded across the subdomains, yet remained considerably weaker than that observed in AD mouse models. Intriguingly, SCA remained the most similar to AD in this analysis (Figure S15).

We performed two additional transcriptome correlation analysis studies across the panoply of neurodegenerative mouse models to further examine the correlation of Kat5 cKO epigenetic dysregulation of specific focal areas of neurobiology with that observed in different disease states. Endolysosomal dysregulation was widely implicated in AD pathogenesis and appeared to be largely AD and FTD‐ALS specific in the biodomain analyses (Figures S10 and S11). The endolysosome subdomain correlation analysis demonstrated significant association between APP, tau, and other AD models and Kat5 cKO across almost all subdomains, with the notable exception of receptor internalization. The correlation signal between AD and Kat5 cKO was stronger than in any other disease state, with the exception of one model in FTD‐ALS and one SCA model (Figure S16).

The final correlation analysis performed looked at the Kat5 cKO transcriptomic signal compared to the AD consensus clusters identified in Wan et al. that originally compared these mouse disease datasets.^37^ The five consensus clusters represent broadly distinct areas of biology, spanning ECM, immune function, neuronal process, cell cycle and organelle biogenesis, and stress response. These are biological process areas in which we in TREAT‐AD observed a strong signal in LOAD.^30^ Performing the transcriptome correlation analysis across the co‐expression consensus clusters showed a remarkable correlation across almost all modules within each cluster across APP, tau, and other AD models (Figure S17). It should be noted that the correlation between Kat5 and the p25/cdk5 mouse model remained the strongest observed within most of the correlation analyses performed, and it showed a statistically significant positive correlation at a strong level within every module within each cluster with Kat5 cKO. This may point to a deep interweaving of Kat5 function and cdk5‐mediated signaling. There is an anti‐correlation between the Kat5 cKO and a few of the APP transgenic mouse models in the forebrain at early developmental stages, but the correlation becomes significant and positive within the same models at later developmental stages. This may point to incremental disease pathogenesis impacting epigenetic dysregulation across AD disease progression. None of the other neurodegenerative disease states were as consistently or strongly correlated with Kat5 cKO across clusters and modules. One of the CJD models was similar to the correlation with AD, but the other was significantly anti‐correlated in the neuronal process cluster. The TAR DNA‐binding protein 43 (TDP‐43) FTD‐ALS mouse model was uniquely similarly correlated, yet the TDP‐43 FTD‐ALS model lacking the nuclear localization signal (NLS) showed no correlation, possibly suggesting some indirect conserved nuclear function between Kat5 and TDP43. The SCA models had no overlap within the neuronal process cluster with Kat5, with several showing an anti‐correlation. There was a significant positive correlation between three SCA models in the four non‐neuronal clusters. HD models showed mostly an anti‐correlation with Kat5 cKO across the consensus clusters, with a small percentage of models showing a weak but significant correlation with Kat5 cKO in the neuronal cluster (Figure S17). Taken together, these analyses suggest a complex interaction between Kat5 and neurodegenerative disease that is most consistent in AD mouse models.

**FIGURE 6:**
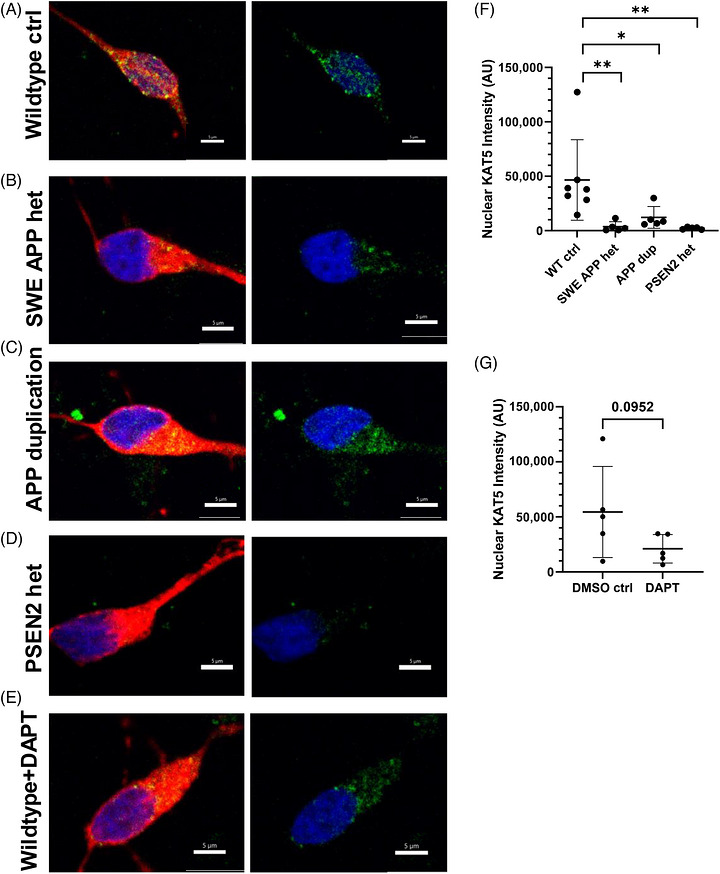
Subcellular localization of KAT5 in human induced pluripotent stem cell (iPSC)‐derived WT and FAD neurons. iPSCs from a WT CViia2 strain, APP Swedish (APPswed), and APP duplication mutants (APPdup, a model of trisomy 21) were differentiated into glutamatergic neurons and stained for KAT5 (green), DAPI (blue) to show nuclear localization, and MAP2 (red) to show cytosolic cytoskeletal structure. The WT cells demonstrate ubiquitous localization (A) with fluorescent overlap with the DAPI nuclear signal. All explored FAD mutant lines (B and C) and APP duplication lines (D) show specific nuclear exclusion of KAT5. DAPT‐treated neurons have decreased levels of KAT5 (E) but not at statistically significant levels (G). The statistical significance was analyzed as described in the Methods section across cells per culture group, with the average for each cell shown as a single dot in the plots on the right (E). The WT cells are statistically significantly different in nuclear signal intensity compared with the APPSWE heterozygous cells (*p* < 0.01), the APP duplication (*p* < 0.05), or PSEN2 N141I heterozygous mutants (*p* < 0.01).

The fundamental hypothesis of this work is that a decrement in γ‐secretase function associated with AD (FAD or LOAD) may contribute to differential subcellular localization of KAT5 through membrane sequestration due to its association with APP and FE65/APBB1. To directly test this idea, we examined iPSC‐derived neurons in WT and AD lineages in two separate sets of studies. In the first study, in WT human neurons, we observed a relatively diffuse state of KAT5 localization throughout the cell (Figure 6A). DAPI staining (blue) demonstrated the nuclear boundaries that KAT5 enters within WT neurons. Examination of KAT5 in heterozygous APP_swe_ (Figure 6B, neurons isogenic to the WT with CRISPR gene editing insertions of the SWE APP heterozygous mutations^55^) demonstrated cytosolic restriction. Similarly, KAT5 was restricted from the nucleus in the APP_dup_ (Figure 6C, neurons carrying a duplicate APP allele) human neurons. Examination of the PSEN2 FAD heterozygous mutant neurons also demonstrated a predominantly cytoplasmic sequestration (Figure 6D). Based on MAP2 staining (red), all neurons appeared healthy and viable. The WT neurons treated with DAPT (γ‐secretase inhibitor) decreased the KAT5 nuclear signal (Figure 6E), although not to a statistically significant degree (Figure 6G). The images were quantitated as described in the Methods section and show significant decreases in nuclear KAT5 staining intensity (Figure 6F). Of interest, the staining in the APP_swe_ and APP_dup_ lines appeared to be largely punctate, likely due to vesicular trafficking. A previous study demonstrated that FAD mutations in APP and PSEN1 induced endolysosomal enlargement and aberrant trafficking of APP.^56^ Our data were consistent, potentially noting that KAT5 remained associated during the endolysosomal pathological progression.

A subcellular localization study was performed in an alternative lab at a different university, using independent techniques described in the Methods section. In the second study, male and female non‐diseased and FAD (APPV717I) hiPSC lines and an APP triplication mutant were grown from neural progenitor cells into excitatory glutamatergic neurons. These neurons were stained for KAT5 with two separate antibodies in independent experiments. These cells were also stained for MAP2 and DAPI to identify the boundaries of the cell and to delineate the nucleus. Similar to the first set of experiments, KAT5 was present in the nucleus and cytoplasm in the non‐diseased cell lines, with greater abundance in the cytoplasm in all three disease lines (Figure S18A). Automated quantitation was performed on KAT5 levels in the nucleus, again showing decreased nuclear KAT5 in FAD lines. The images shown employed the Protein Tech antibody to validate the results with two separate antibodies in two independent studies. The quantitation performed depicts the imaging results with both antibodies in the second study. The quantitation showed significant decreases in KAT5 nuclear intensity in FAD neurons with both antibodies (Figure S18B). These data directly support the central hypothesis of this work.

**FIGURE 7:**
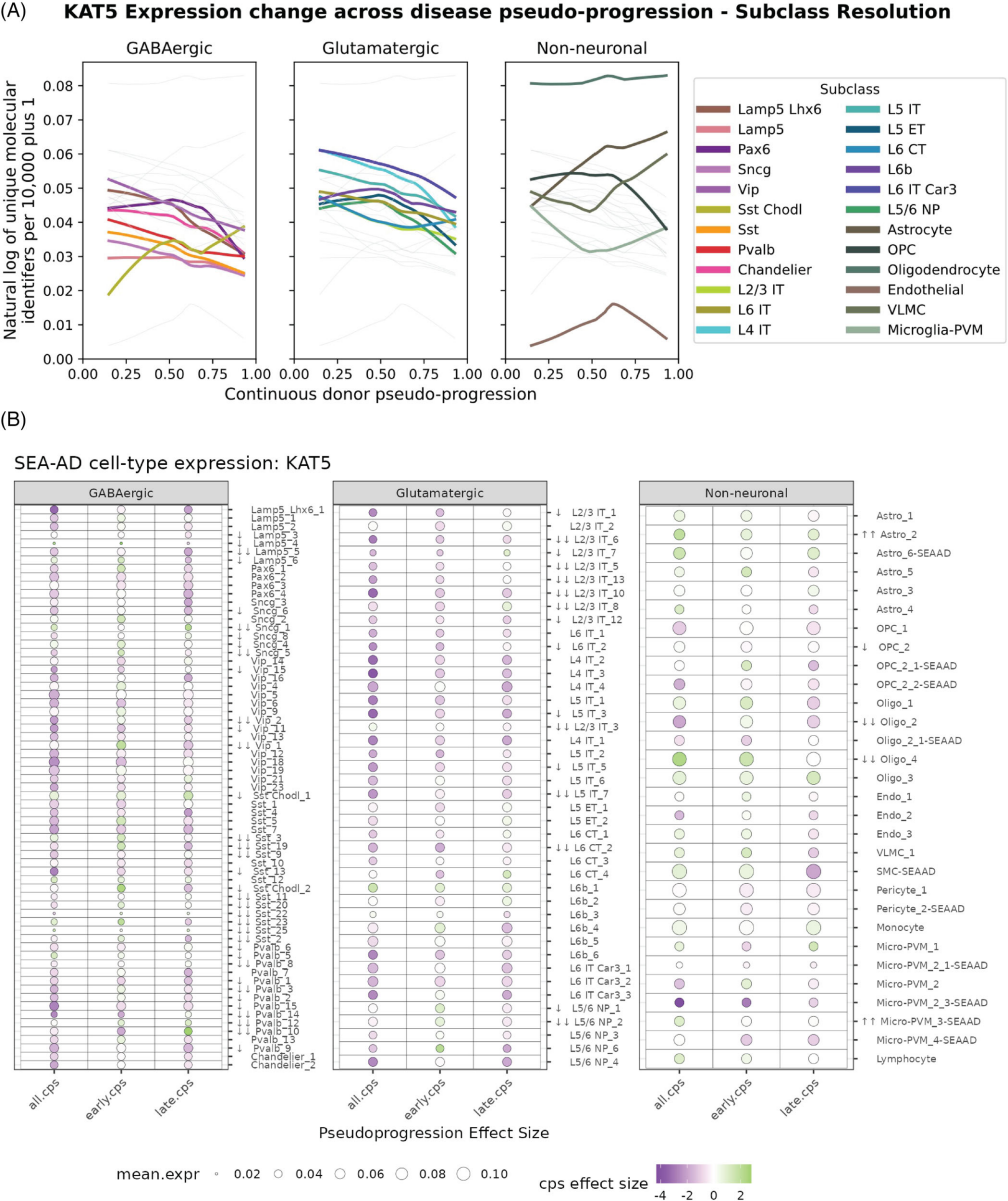
KAT5 downregulation in SEA‐AD LOAD patients. The Seattle Alzheimer's Disease (SEA‐AD) study performed single‐cell expression analysis across the whole taxonomy of neuronal and glial lineages. The grouped cellular subtypes are shown in (A) within inhibitory GABAergic neurons (left), excitatory glutamatergic neurons (middle), and non‐neuronal glial lineages (right) shown as normalized expression level across disease pseudo‐progression. All cellular lineages are shown in panel (B), broken down into the GABAergic, glutamatergic, and non‐neuronal lines with changes in expression denoted by continuous pseudo‐progression (CPS) effect size, with downregulation in purple and upregulation in green. Both representations of the data show downward progression of KAT5 levels in neurons across disease progression (CPS). KAT5 downregulation is consistently noted in the early stage within more superficial cortical layers (L2/3) and some somatostatin GABAergic neurons, which represent the most sensitive neurons identified in the SEA‐AD study.

The subcellular localization data support the idea that KAT5 may play a role in FAD, yet it leaves its possible role in LOAD uninvestigated. To assess whether KAT5 may contribute to LOAD pathoprogression, we examined the single‐cell expression patterns of KAT5 and all components of the heterotetrameric γ‐secretase complex in LOAD brains characterized in the recent SEA‐AD study.^50^ We explored KAT5 expression levels across LOAD stages in the SEA‐AD study to determine whether changes in KAT5 levels might be associated with LOAD progression. KAT5 levels decrement rapidly across LOAD starting at the earliest stages of pathoprogression (Figure 7A). The specific cell types were delineated at two different resolutions with cellular subtypes in Figure 7A and the more resolved supertypes in Figure 7B. Intriguingly, we know that KAT5 is important for neuronal survival (Figure 4), and the earliest vulnerable neurons identified in the study are the somatostatin (SST) and parvalbumin (PVALB) inhibitory neurons.^50^ These two classes of neurons demonstrated steep declines in KAT5 levels in the early stages of disease and continued to decline across the pathoprogression (Figure 7A,B). The changes in KAT5 levels are depicted across unique CNS cellular lineages (Figure 7B), showing decrements limited to neuronal cell populations. Given the upregulation of APP protein levels in AD human *post mortem* brain,^57^ the downregulation of KAT5 could result in an elevated proportion of KAT5 localized to the membrane.

Consistent with reports that FAD mutations induce an enzymatic stalling of the γ‐secretase complex with the APP substrate,^18^ we observed downregulation of elements of the heterotetrameric γ‐secretase complex across AD progression, modeled in the SEA‐AD study by a neuropathologically derived continuous pseudoprogression score (CPS) (Figure S19). We specifically observed decrements in PSEN2 (Figure S19B) and APH1B (Figure S19D) within both inhibitory and excitatory neurons. Additionally, there was a decrement in nicastrin (NCSTN) (Figure S19E) in the later stage of AD progression, particularly in the inhibitory neuronal populations. These data imply that KAT5 dysregulation may occur in LOAD by decreases in KAT5 expression and decreases in its release from APP due to downregulation of the γ‐secretase complex. The increase in APP protein, the decrement in key components of the γ‐secretase complex, and the decrease of KAT5 expression across disease stages would predict that KAT5 nuclear function would diminish across AD pathoprogression.

**FIGURE 8:**
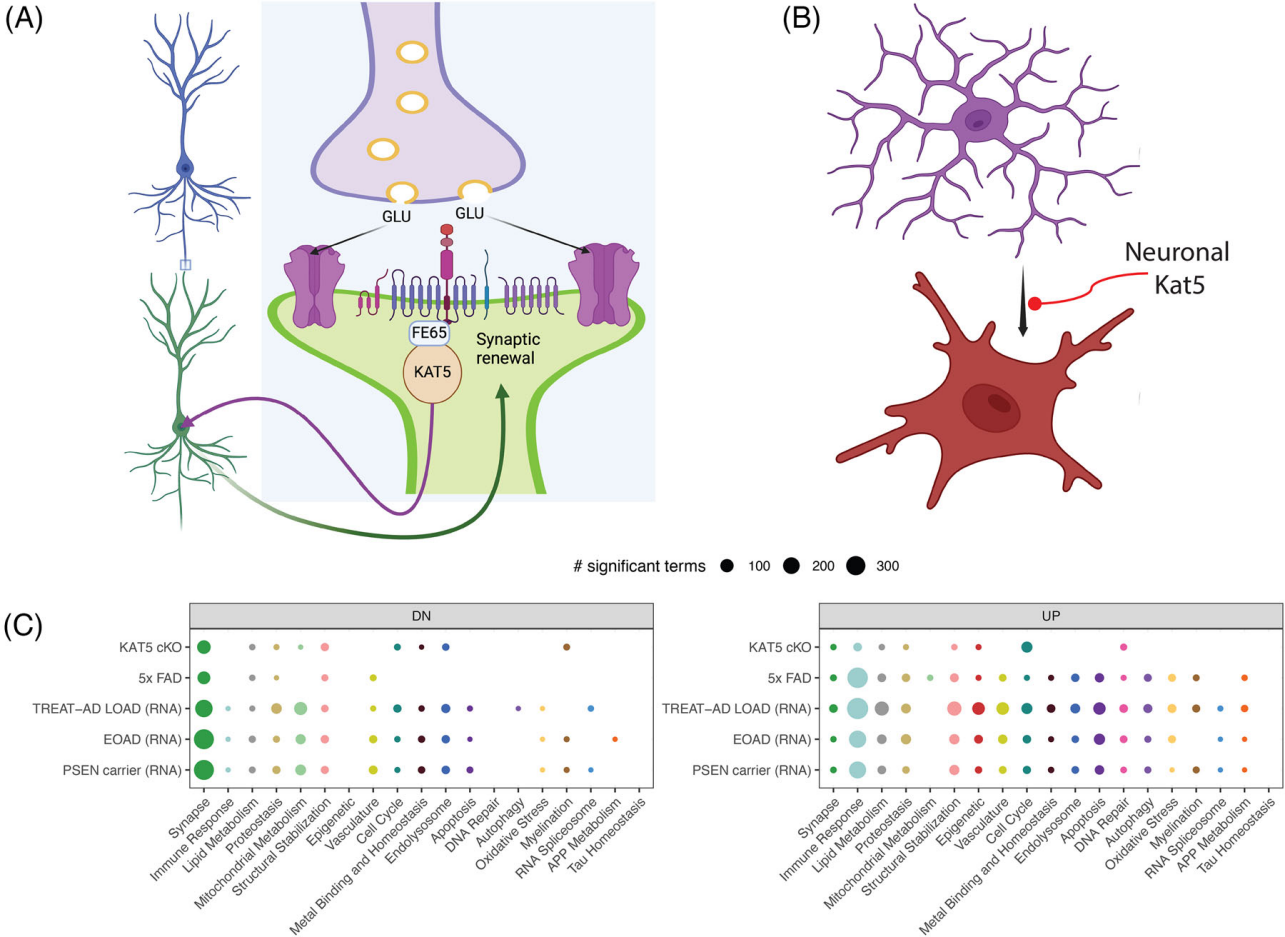
Hypothetical model of KAT5 biological function in neurons and microglia. (A) The data point to a biological process in which KAT5 promotes synaptic gene expression fostering synaptic development. KAT5 associates with APP via the APBB1/FE65 binding protein and following γ‐secretase‐mediated cleavage travels to the nucleus to stimulate synaptic gene expression and promote neuronal homeostasis. (B) The Kat5 cKO mouse model is neuron specific and results in the activation of innate and adaptive immune functions. This suggests that Kat5 neuronal function performs feedback control to maintain innate immune microglial and astrocytic cells in a quiescent state. Consequently, a lack of KAT5 nuclear signaling would be predicted to decrease synaptic gene expression and increase immune response genes associated with reactive gliosis. (C) Comparing all the models explored in this study across all 19 biological domains for both downregulated and upregulated gene sets, we see a consistent decrease in synaptic signal across all models, including KAT5, as well as increases in immune response, consistent with the hypothesis suggested in (A) and (B). (A) and (B) were generated using BioRender software.

The observation KAT5 promotes neuronal survival through the epigenetic regulation of critical neuronal genes supports the hypothesis that KAT5 decreases in function could play a plausible role in AD pathoprogression through disruption of neuronal homeostasis (this hypothesis is depicted in Figure 8).

## 4 DISCUSSION

The core question investigated in this work is whether KAT5‐mediated epigenetic regulation could be involved in normal neurological homeostasis, whose disruption may dysregulate CNS processes and catalyze the neurodegenerative cascade observed in AD. KAT5 is known to be critical in development, as the Kat5 KO mouse is embryonically lethal in the early blastocyst stage,^58^ and CNS homozygous ablation of KAT5 leads to the malformation of cortical tissue and microencephaly,^59^ suggesting a prominent role for epigenetic regulation in early CNS development. Identified hypomorphic missense variants in KAT5 are linked to cerebellar encephalopathy and decreases in cognitive function in variant carriers, further supporting the linkage between KAT5 and neurological development.^60^ Our hypothesis is that KAT5 disruption of function prevents epigenetic activation of neuronal homeostatic genes, promoting neurodegenerative sequelae in AD.

To test the KAT5 epigenetic homeostasis hypothesis, we re‐analyzed the CNS inducible KO of the Kat5 mouse gene^23^ and aligned its differential gene expression pattern to the AD biological domains and subdomains developed in the TREAT‐AD consortium. Consistent with the original characterization,^23^ we observed broad disruption of synaptic function in the Kat5 cKO mouse (Figures 1 and 2). We further observed similar biodomain enrichments between the Kat5 cKO and the 5xFAD mouse model, EOAD, FAD, and LOAD (Figures 2 and 3). FAD mutations in APP or PSEN1 result in the enzyme–substrate complex stalling at the intermediate transition state, preventing completion of APP proteolysis,^18^ reducing AICD production, and potentially trapping KAT5 at the synaptic membrane. The primary prediction of KAT5 membrane sequestration is altered nuclear chromatin regulation, which may impair essential neuronal homeostasis. In our characterization of the Kat5cKO, we observed a profound synaptic phenotype, as systemic downregulation of synaptic gene expression is the core transcriptomic signature (Figures 1 and 3), identifying both excitatory and inhibitory pathways (Figure 1, Figure S1). The upregulated DEGs in the Kat5 mouse implicate cell cycle, DNA repair, and immune response (Figures 1A,C,D and 2), signatures present in LOAD (Figure S2).

Direct comparison of the Kat5 cKO mouse model transcriptomic signature to LOAD, EOAD, FAD, and the 5xFAD mouse (Figure 3A) showed overlapping patterns of biological domain enrichment in synapse, immune response, cell cycle, and others (Figures 2 and 3). The correlation between Kat5 cKO DEGs and the comparative AD datasets was weak but highly significant (Figure 3B) due to the elevated correlation observed in specific overlapping biological domains (Figure 3C), most consistently synapse and immune response, with overlap between Kat5 cKO and the human datasets observed in cell cycle and endolysosome domains as well (Figure 3C). Intriguingly, we observed upregulation of complement cascades and immune migration pathways activated across all models (Figure S3, Figure 3D), which is consistent with the shifts in the Kat5 cKO from homeostatic to activated disease‐associated microglial markers (Figure S6). While the Kat5 cKO showed little mitochondrial metabolic impairment at the biodomain level, we observed downregulation of metabolic pathways involved in glucose transport, glycolysis, TCA cycle, and ETC (Figure S5). KAT5 dysregulated genes demonstrated enriched trait‐variant linkage with cognition and AD pathology (Figure S7), providing genetic linkage evidence in support of epigenetic dysregulation contributing to AD pathoprogression.

KAT5 nuclear signaling was explored in subcellular localization studies in human WT/non‐diseased and AD iPSC‐derived human neurons in two independently executed sets of experiments within separate laboratories at different universities. In the first study, we compared KAT5 localization in hiPSC‐derived neurons from WT and those carrying the SWE APP, an APP duplication, and PSEN2 N141I FAD mutations. The WT cells demonstrated a ubiquitous distribution of KAT5 throughout the cell, while neurons carrying FAD mutations showed punctate cytosolic sequestration (Figure 6). The second independent study also compared non‐diseased hiPSC‐derived neurons to FAD lineages and found decreases in nuclear KAT5 levels in FAD, quantitated with two independent KAT5 antibodies (Figure S18A,B). These data are consistent with previous reports of nuclear exclusion in human hippocampal neurons in *post mortem* AD brains.^29^ The relevance to LOAD is two‐fold: first, a re‐analysis of the SEA‐AD study showed decreases in KAT5 early in LOAD pathoprogression within inhibitory and excitatory neurons (Figure 7), including the somatastatin and parvalbumin inhibitory neurons impacted early in AD; second, core components of the heterotetrameric γ‐secretase complex (especially PSEN2 and APH1B, Figure S19) are downregulated in most neuronal lineages in LOAD. The increase in APP protein levels in AD, coupled with the decrease in both KAT5 and γ‐secretase, suggest the fraction of KAT5 bound at synaptic membrane is likely higher. Additionally, the decreases in γ‐secretase components suggest lower levels of proteolytic release from the membrane across AD pathoprogression.

To assess the similarity of the Kat5 cKO transcriptomic profile across neurodegenerative disease states, we examined the TREAT‐AD LOAD transcriptomic data, the LOAD diversity cohort (LOAD_DC), FTD (all three subtypes), PD, HD, and ALS. We performed correlation mapping of each disease state expression dataset to the Kat5 cKO transcriptome at both the biodomain (Figure 5A) and subdomain levels (Figure 5B). As expected, enrichments were observed across disease states, with LOAD showing the most significant correlation in synapse and endolysosome, with LOAD_DC also correlating in epigenetics. Many of the shared correlations observed in LOAD are also observed in other neurodegenerative diseases, most notably FTD (MAPT and GRN subtypes) and PD. At the subdomain level, we observed a strong correlation across the synapse and endolysosome subdomains shared by LOAD, FTD (MAPT and GRN), and PD (Figure 5B). This is consistent with recent observations noting similarities in expression patterns in AD, FTD, and PD,^53^, ^54^ likely reflecting shared neurodegenerative disease processes.

We investigated the correlation of the Kat5 cKO with the neurodegenerative mouse models explored by Wan et al. in the development of the AD co‐expression consensus clusters.^37^ In doing so, we discovered a broad commonality of the synapse biodomain genes dysregulated in Kat5 cKO in other neurodegenerative states (Figure S10). Our initial analysis entailed a GO term‐based correlation of GSEA enrichments. The strength of this approach is that it identifies common areas of biology shared by models or disease states. However, the approach does not require the same genes to be regulated within each enrichment for the correlation to be positive. Consequently, we repeated the analysis performing a transcriptome‐wide correlation analysis for all genes annotated to either the biodomains or subdomains. Intriguingly, shifting from a process correlation to a gene‐centric correlation profoundly altered the results, expanding the scope of overlap observed within the AD models, including tau models, and decreased the correlation between Kat5 and other neurodegenerative disease state models (Figure S11). We repeated this process in synapse and immune response and obtained similar results (Figure S12–S15). Also of interest, the gene‐centric correlation of Kat5 cKO with AD mouse models across the immune response subdomains showed remarkable AD specificity (Figure S15). This is particularly interesting in light of Kat5 cKO being a neuron‐specific genetic deletion. This suggests a strong role of neuronal epigenetic regulation in the immune response processes in AD. Additionally, there is strong correlation of the Kat5 cKO transcriptome with the AD mouse models across the AD co‐expression consensus clusters (Figure S17). These clusters were identified by their association with AD disease state, so this supports the conceptual involvement of Kat5 in AD pathoprogression.

There are numerous limitations in this analysis, in terms of both the biological models and the analytical methodology. One limitation is the deployment of transcriptomic sequencing in an early developmental stage mouse (10‐12 weeks postnatal at sequencing), which precedes any observed pathology and may minimize the impact on the emergent molecular signatures. Additionally, we had no transcriptomic data from a human KAT5 model, limiting our power to interrogate any species‐specific effects. We saw higher AD specificity in the mouse model correlation analysis, which may be partially due to species‐specific regulatory effects of KAT5. This investigation was also limited by the size and scope of the datasets available. Future explorations will expand the scope of the datasets to those emerging on the horizon. Additionally, as KAT5 regulates critical neuronal survival genes (Figure 4), elucidating its role in diseases‐specific regulation will require more direct biological investigation to identify its specific role in AD and other diseases states.

Collectively, the observations here support a model in which KAT5 epigenetic regulation promotes neuronal homeostasis (Figure 8A,C). KAT5 cytoplasmic sequestration is observed in human AD neurons (Figure 6, Figures S20 and S18) and has been observed within LOAD hippocampal tissue,^29^ and alteration of the KAT5/HDAC2 levels are associated with increased cognitive performance,^22^, ^29^ which is consistent with the Kat5 subcellular localization to the nucleus being critical to learning.^19^ Future investigations will be necessary to understand the viability of this mechanism in human systems.

## CONFLICT OF INTEREST STATEMENT

Allan I. Levey is a paid consultant for EmTheraPro, Cognito Therapeutics, Cognition Therapeutics, and Alamar. Gregory W. Carter is a paid consultant for Astrex Pharmaceuticals. All other authors have no conflict of interest. Author disclosures are available in the Supporting Information.

## CONSENT STATEMENT

No human subjects were employed in this work. All human data employed were de‐identified and are publicly available for use. The utilization of human datasets from LOAD patients were approved through governance Institutional Review Boards at Sage Bionetworks: Western Institutional Review Board – Copernicus Group (WCG).

## Supporting information




Supporting Information

**Supporting Information There are two replicates of the Supplementary Figures. 0001‐Figures.pdf should be deleted and 0002‐SupMat.pdf should be retained as the Supplementary Figures file**.
