# Supplementary material for: Kat5 cKO mouse replicates biological domain signatures associated with Alzheimer's disease
Source: Alzheimers Dement. 2026 Jun 30;22(7):e71562. doi: 10.1002/alz.71562 (PMC13319415; doi:10.1002/alz.71562)
Supplement: Supplementary file 1 — Supporting Information [file ALZ-22-e71562-s002.pdf]

Figure S1

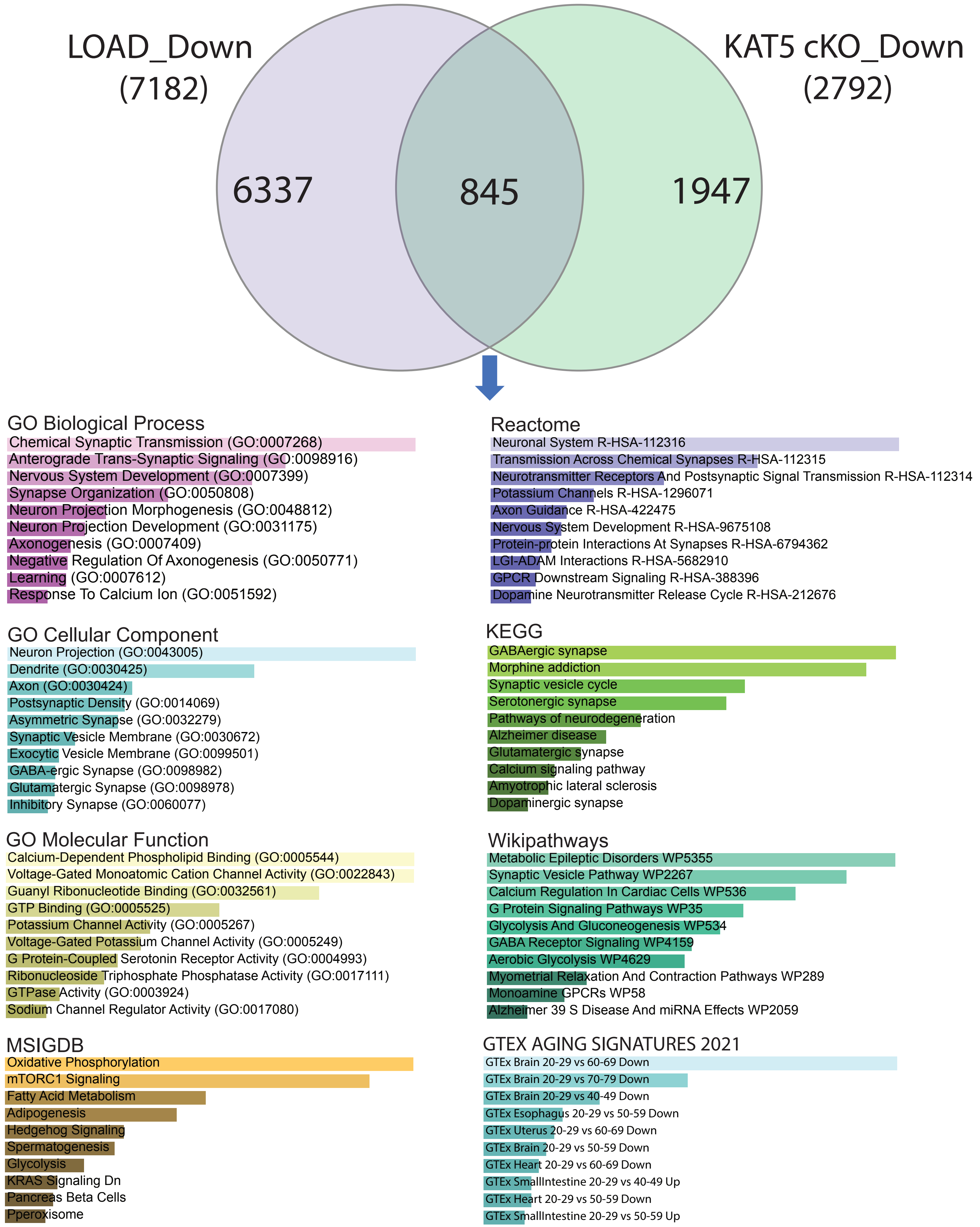

LOAD_Down
(7182)
KAT5 cKO_Down
(2792)
6337
845
1947
GO Biological Process
Chemical Synaptic Transmission (GO:0007268)
Anterograde Trans-Synaptic Signaling (GO:0098916)
Nervous System Development (GO:0007399)
Synapse Organization (GO:0050808)
Neuron Projection Morphogenesis (GO:0048812)
Neuron Projection Development (GO:0031175)
Axonogenesis (GO:0007409)
Negative Regulation Of Axonogenesis (GO:0050771)
Learning (GO:0007612)
Response To Calcium Ion (GO:0051592)
Reactome
Neuronal System R-HSA-112316
Transmission Across Chemical Synapses R-HSA-112315
Neurotransmitter Receptors And Postsynaptic Signal Transmission R-HSA-112314
Potassium Channels R-HSA-1296071
Axon Guidance R-HSA-422475
Nervous System Development R-HSA-9675108
Protein-protein Interactions At Synapses R-HSA-6794362
LGI-ADAM Interactions R-HSA-5682910
GPCR Downstream Signaling R-HSA-388396
Dopamine Neurotransmitter Release Cycle R-HSA-212676
GO Cellular Component
Neuron Projection (GO:0043005)
Dendrite (GO:0030425)
Axon (GO:0030424)
Postsynaptic Density (GO:0014069)
Asymmetric Synapse (GO:0032279)
Synaptic Vesicle Membrane (GO:0030672)
Exocytic Vesicle Membrane (GO:0099501)
GABA-ergic Synapse (GO:0098982)
Glutamatergic Synapse (GO:0098978)
Inhibitory Synapse (GO:0060077)
KEGG
GABAergic synapse
Morphine addiction
Synaptic vesicle cycle
Serotonergic synapse
Pathways of neurodegeneration
Alzheimer disease
Glutamatergic synapse
Calcium signaling pathway
Amyotrophic lateral sclerosis
Dopaminergic synapse
GO Molecular Function
Calcium-Dependent Phospholipid Binding (GO:0005544)
Voltage-Gated Monoatomic Cation Channel Activity (GO:0022843)
Guanyl Ribonucleotide Binding (GO:0032561)
GTP Binding (GO:0005525)
Potassium Channel Activity (GO:0005267)
Voltage-Gated Potassium Channel Activity (GO:0005249)
G Protein-Coupled Serotonin Receptor Activity (GO:0004993)
Ribonucleoside Triphosphate Phosphatase Activity (GO:0017111)
GTPase Activity (GO:0003924)
Sodium Channel Regulator Activity (GO:0017080)
Wikipathways
Metabolic Epileptic Disorders WP5355
Synaptic Vesicle Pathway WP2267
Calcium Regulation In Cardiac Cells WP536
G Protein Signaling Pathways WP35
Glycolysis And Gluconeogenesis WP534
GABA Receptor Signaling WP4159
Aerobic Glycolysis WP4629
Myometrial Relaxation And Contraction Pathways WP289
Monoamine GPCRs WP58
Alzheimer 39 S Disease And miRNA Effects WP2059
MSIGDB
Oxidative Phosphorylation
mTORC1 Signaling
Fatty Acid Metabolism
Adipogenesis
Hedgehog Signaling
Spermatogenesis
Glycolysis
KRAS Signaling Dn
Pancreas Beta Cells
Pperoxisome
GTEX AGING SIGNATURES 2021
GTEx Brain 20-29 vs 60-69 Down
GTEx Brain 20-29 vs 70-79 Down
GTEx Brain 20-29 vs 40-49 Down
GTEx Esophagus 20-29 vs 50-59 Down
GTEx Uterus 20-29 vs 60-69 Down
GTEx Brain 20-29 vs 50-59 Down
GTEx Heart 20-29 vs 60-69 Down
GTEx SmallIntestine 20-29 vs 40-49 Up
GTEx Heart 20-29 vs 50-59 Down
GTEx SmallIntestine 20-29 vs 50-59 Up

Supplementary Figure 1. Convergence of Down-Regulated Genes in Kat5 cKO and LOAD Across GO and Pathway Models.

Drawing from our recent work harmonizing over 1700 postmortem brain bank transcriptomic profiles, we identify a set of 7182 significantly down-regulated genes within LOAD and compare those to the 2792 unique down-regulated Kat5 cKO genes ($p<0.05$, 0.2 differential expression cut-off). There are 845 genes shared between the two groups that are employed in enrichment studies of GO term enrichment within each of the primary tiers of GO (biological process, cellular component, and molecular function). Pathway enrichment analysis was performed using the same 845 genes across Reactome, KEGG, and WikiPathways. The 845 shared genes overlap the GTEX signatures for brain aging (brain 20-29 vs 60-69; 20-29 vs 70-79) most highly, consistent with the alignment of the Kat5 cKO model with human brain aging. All colored bars are significant at $p<0.05$, with shorter bars denoting decreased p-values and darker colors representing decreasing enrichment scores. All values are available in Supplementary Table 2.

Figure S2

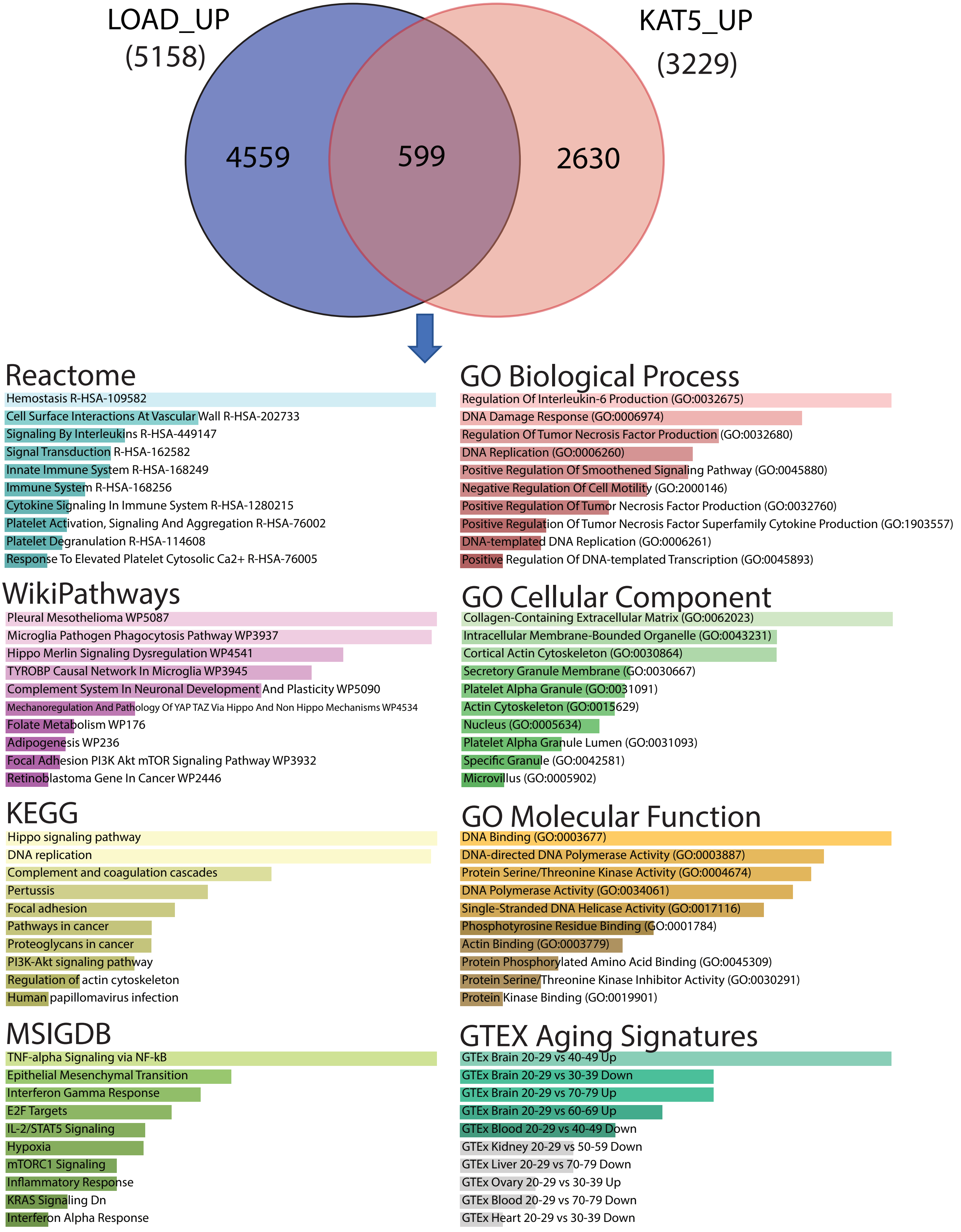
LOAD_UP
(5158)
KAT5_UP
(3229)
4559
599
2630
Reactome
Hemostasis R-HSA-109582
Cell Surface Interactions At Vascular Wall R-HSA-202733
Signaling By Interleukins R-HSA-449147
Signal Transduction R-HSA-162582
Innate Immune System R-HSA-168249
Immune System R-HSA-168256
Cytokine Signaling In Immune System R-HSA-1280215
Platelet Activation, Signaling And Aggregation R-HSA-76002
Platelet Degranulation R-HSA-114608
Response To Elevated Platelet Cytosolic Ca2+ R-HSA-76005
GO Biological Process
Regulation Of Interleukin-6 Production (GO:0032675)
DNA Damage Response (GO:0006974)
Regulation Of Tumor Necrosis Factor Production (GO:0032680)
DNA Replication (GO:0006260)
Positive Regulation Of Smoothened Signaling Pathway (GO:0045880)
Negative Regulation Of Cell Motility (GO:2000146)
Positive Regulation Of Tumor Necrosis Factor Production (GO:0032760)
Positive Regulation Of Tumor Necrosis Factor Superfamily Cytokine Production (GO:1903557)
DNA-templated DNA Replication (GO:0006261)
Positive Regulation Of DNA-templated Transcription (GO:0045893)
WikiPathways
Pleural Mesothelioma WP5087
Microglia Pathogen Phagocytosis Pathway WP3937
Hippo Merlin Signaling Dysregulation WP4541
TYROBP Causal Network In Microglia WP3945
Complement System In Neuronal Development And Plasticity WP5090
Mechanoregulation And Pathology Of YAP TAZ Via Hippo And Non Hippo Mechanisms WP4534
Folate Metabolism WP176
Adipogenesis WP236
Focal Adhesion PI3K Akt mTOR Signaling Pathway WP3932
Retinoblastoma Gene In Cancer WP2446
GO Cellular Component
Collagen-Containing Extracellular Matrix (GO:0062023)
Intracellular Membrane-Bounded Organelle (GO:0043231)
Cortical Actin Cytoskeleton (GO:0030864)
Secretory Granule Membrane (GO:0030667)
Platelet Alpha Granule (GO:0031091)
Actin Cytoskeleton (GO:0015629)
Nucleus (GO:0005634)
Platelet Alpha Granule Lumen (GO:0031093)
Specific Granule (GO:0042581)
Microvillus (GO:0005902)
KEGG
Hippo signaling pathway
DNA replication
Complement and coagulation cascades
Pertussis
Focal adhesion
Pathways in cancer
Proteoglycans in cancer
PI3K-Akt signaling pathway
Regulation of actin cytoskeleton
Human papillomavirus infection
GO Molecular Function
DNA Binding (GO:0003677)
DNA-directed DNA Polymerase Activity (GO:0003887)
Protein Serine/Threonine Kinase Activity (GO:0004674)
DNA Polymerase Activity (GO:0034061)
Single-Stranded DNA Helicase Activity (GO:0017116)
Phosphotyrosine Residue Binding (GO:0001784)
Actin Binding (GO:0003779)
Protein Phosphorylated Amino Acid Binding (GO:0045309)
Protein Serine/Threonine Kinase Inhibitor Activity (GO:0030291)
Protein Kinase Binding (GO:0019901)
MSIGDB
TNF-alpha Signaling via NF-kB
Epithelial Mesenchymal Transition
Interferon Gamma Response
E2F Targets
IL-2/STAT5 Signaling
Hypoxia
mTORC1 Signaling
Inflammatory Response
KRAS Signaling Dn
Interferon Alpha Response
GTEX Aging Signatures
GTEx Brain 20-29 vs 40-49 Up
GTEx Brain 20-29 vs 30-39 Down
GTEx Brain 20-29 vs 70-79 Up
GTEx Brain 20-29 vs 60-69 Up
GTEx Blood 20-29 vs 40-49 Down
GTEx Kidney 20-29 vs 50-59 Down
GTEx Liver 20-29 vs 70-79 Down
GTEx Ovary 20-29 vs 30-39 Up
GTEx Blood 20-29 vs 70-79 Down
GTEx Heart 20-29 vs 30-39 Down

Figure S2. Convergence of Up-regulated Genes in Kat5 cKO and LOAD Across GO and Pathway Models.

The TREAT-AD Consortium Bioinformatics Team harmonized over 1700 transcriptomic profiles from harmonized postmortem brain banks. We employed the up-regulated differentially expressed genes (DEG, total 5158) ($p<0.05$) in a comparison with Kat5 cKO up-regulated DEGs (0.2 differential expression cut-off, $p<0.05$, total 3229). The Venn diagram demonstrates the intersection of these two genesets with 599 genes shared between the two datasets. Gene set enrichment analysis was performed with the 599 overlapping genes with all three tiers of GO, select pathway models (Reactome, KEGG, WikiPathways), and the molecular signatures associated with MSIGDB and GTEX aging profiles. All colored bars are significant at $p<0.05$, with shorter bars denoting decreased p-values and darker colors representing decreasing enrichment scores. All values are available in Supplementary Table 3.

Figure S3

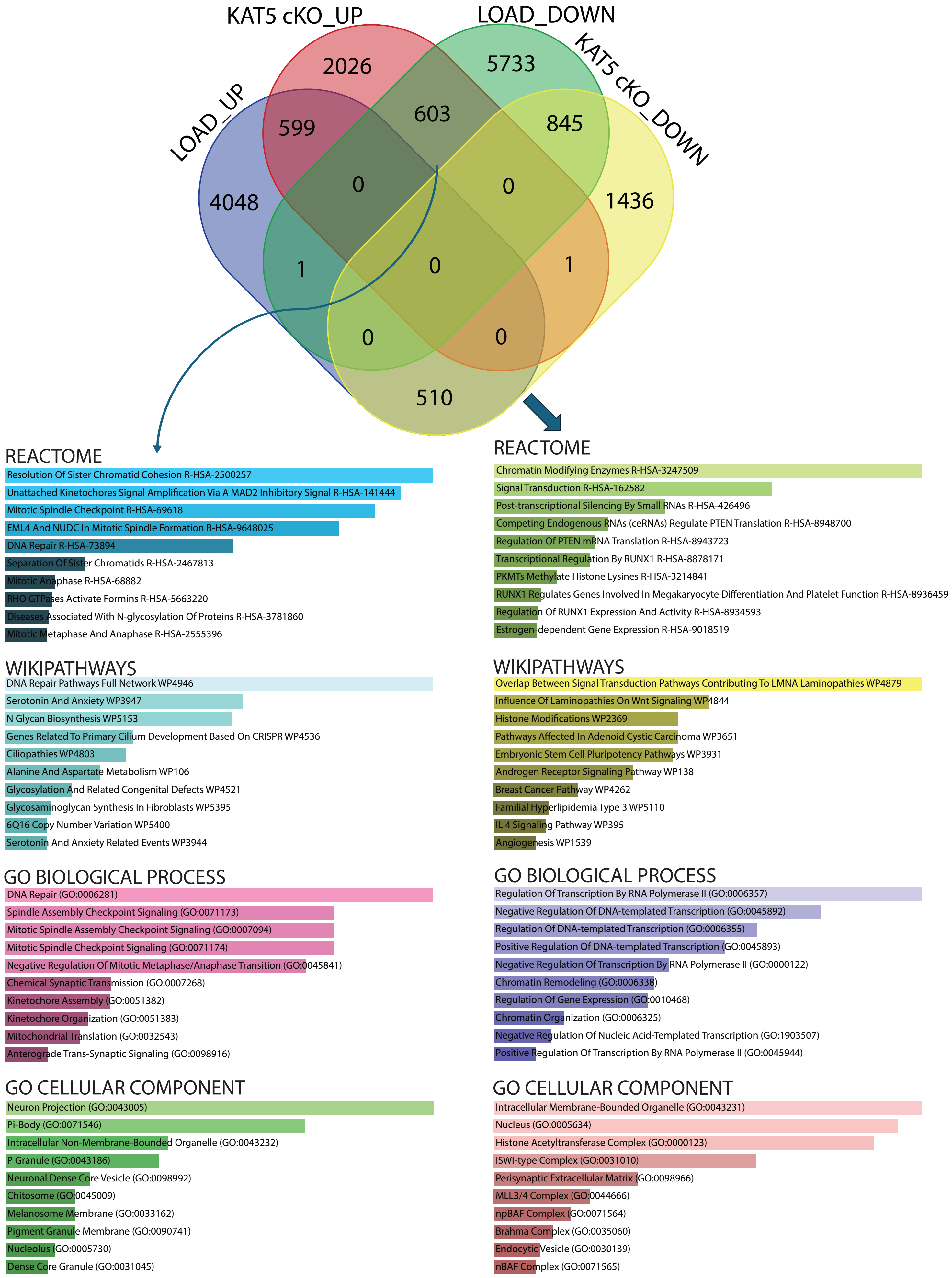

KAT5 cKO_UP
LOAD_DOWN
LOAD_UP
KAT5 cKO_DOWN
2026
5733
599
603
845
4048
0
0
1436
1
0
1
0
0
510
REACTOME
Resolution Of Sister Chromatid Cohesion R-HSA-2500257
Unattached Kinetochores Signal Amplification Via A MAD2 Inhibitory Signal R-HSA-141444
Mitotic Spindle Checkpoint R-HSA-69618
EML4 And NUDC In Mitotic Spindle Formation R-HSA-9648025
DNA Repair R-HSA-73894
Separation Of Sister Chromatids R-HSA-2467813
Mitotic Anaphase R-HSA-68882
RHO GTPases Activate Formins R-HSA-5663220
Diseases Associated With N-glycosylation Of Proteins R-HSA-3781860
Mitotic Metaphase And Anaphase R-HSA-2555396
WIKIPATHWAYS
DNA Repair Pathways Full Network WP4946
Serotonin And Anxiety WP3947
N Glycan Biosynthesis WP5153
Genes Related To Primary Cilium Development Based On CRISPR WP4536
Ciliopathies WP4803
Alanine And Aspartate Metabolism WP106
Glycosylation And Related Congenital Defects WP4521
Glycosaminoglycan Synthesis In Fibroblasts WP5395
6Q16 Copy Number Variation WP5400
Serotonin And Anxiety Related Events WP3944
GO BIOLOGICAL PROCESS
DNA Repair (GO:0006281)
Spindle Assembly Checkpoint Signaling (GO:0071173)
Mitotic Spindle Assembly Checkpoint Signaling (GO:0007094)
Mitotic Spindle Checkpoint Signaling (GO:0071174)
Negative Regulation Of Mitotic Metaphase/Anaphase Transition (GO:0045841)
Chemical Synaptic Transmission (GO:0007268)
Kinetochore Assembly (GO:0051382)
Kinetochore Organization (GO:0051383)
Mitochondrial Translation (GO:0032543)
Anterograde Trans-Synaptic Signaling (GO:0098916)
GO CELLULAR COMPONENT
Neuron Projection (GO:0043005)
Pi-Body (GO:0071546)
Intracellular Non-Membrane-Bounded Organelle (GO:0043232)
P Granule (GO:0043186)
Neuronal Dense Core Vesicle (GO:0098992)
Chitosome (GO:0045009)
Melanosome Membrane (GO:0033162)
Pigment Granule Membrane (GO:0090741)
Nucleolus (GO:0005730)
Dense Core Granule (GO:0031045)
REACTOME
Chromatin Modifying Enzymes R-HSA-3247509
Signal Transduction R-HSA-162582
Post-transcriptional Silencing By Small RNAs R-HSA-426496
Competing Endogenous RNAs (ceRNAs) Regulate PTEN Translation R-HSA-8948700
Regulation Of PTEN mRNA Translation R-HSA-8943723
Transcriptional Regulation By RUNX1 R-HSA-8878171
PKMTs Methylate Histone Lysines R-HSA-3214841
RUNX1 Regulates Genes Involved In Megakaryocyte Differentiation And Platelet Function R-HSA-8936459
Regulation Of RUNX1 Expression And Activity R-HSA-8934593
Estrogen-dependent Gene Expression R-HSA-9018519
WIKIPATHWAYS
Overlap Between Signal Transduction Pathways Contributing To LMNA Laminopathies WP4879
Influence Of Laminopathies On Wnt Signaling WP4844
Histone Modifications WP2369
Pathways Affected In Adenoid Cystic Carcinoma WP3651
Embryonic Stem Cell Pluripotency Pathways WP3931
Androgen Receptor Signaling Pathway WP138
Breast Cancer Pathway WP4262
Familial Hyperlipidemia Type 3 WP5110
IL 4 Signaling Pathway WP395
Angiogenesis WP1539
GO BIOLOGICAL PROCESS
Regulation Of Transcription By RNA Polymerase II (GO:0006357)
Negative Regulation Of DNA-templated Transcription (GO:0045892)
Regulation Of DNA-templated Transcription (GO:0006355)
Positive Regulation Of DNA-templated Transcription (GO:0045893)
Negative Regulation Of Transcription By RNA Polymerase II (GO:0000122)
Chromatin Remodeling (GO:0006338)
Regulation Of Gene Expression (GO:0010468)
Chromatin Organization (GO:0006325)
Negative Regulation Of Nucleic Acid-Templated Transcription (GO:1903507)
Positive Regulation Of Transcription By RNA Polymerase II (GO:0045944)
GO CELLULAR COMPONENT
Intracellular Membrane-Bounded Organelle (GO:0043231)
Nucleus (GO:0005634)
Histone Acetyltransferase Complex (GO:0000123)
ISWI-type Complex (GO:0031010)
Perisynaptic Extracellular Matrix (GO:0098966)
MLL3/4 Complex (GO:0044666)
npBAF Complex (GO:0071564)
Brahma Complex (GO:0035060)
Endocytic Vesicle (GO:0030139)
nBAF Complex (GO:0071565)

Figure Legend S3. Convergence Patterns of Up- and Down-regulated Genes from LOAD and Kat5 cKO.

The LOAD DEGs identified and compared in Supplementary Figures 1 and 2 are employed here in association with the Kat5 cKO DEGs compared in the same figures. The across valence comparison for LOAD and Kat5 cKO mouse model demonstrate that, in addition to the tandem up or down DEGs, there are also substantial gene sets that are cross-regulated within LOAD and Kat5 cKO. The four-way Venn diagram at the top of the figure shows are intersections, including those already demonstrated in the previous figures, and additionally show a set of 603 genes that are up-regulated in Kat5 cKO and down-regulated in LOAD. Conversely, there is a set of 510 genes that are up-regulated in LOAD and down-regulated in Kat5 cKO. Both cross-regulated gene sets are examined with gene set enrichment analysis using Reactome and WikiPathways pathway models and GO biological process and GO Cellular Component. The up-regulated Kat5 cKO genes down-regulated in LOAD prominently reflect DNA repair and cell cycle processes while the down-regulated Kat5 cKO and up-regulated LOAD genes represent largely transcriptional or chromatin modifying processes. All colored bars are significant at $p<0.05$, with shorter bars denoting decreased p-values and darker colors representing decreasing enrichment scores.

Figure S4

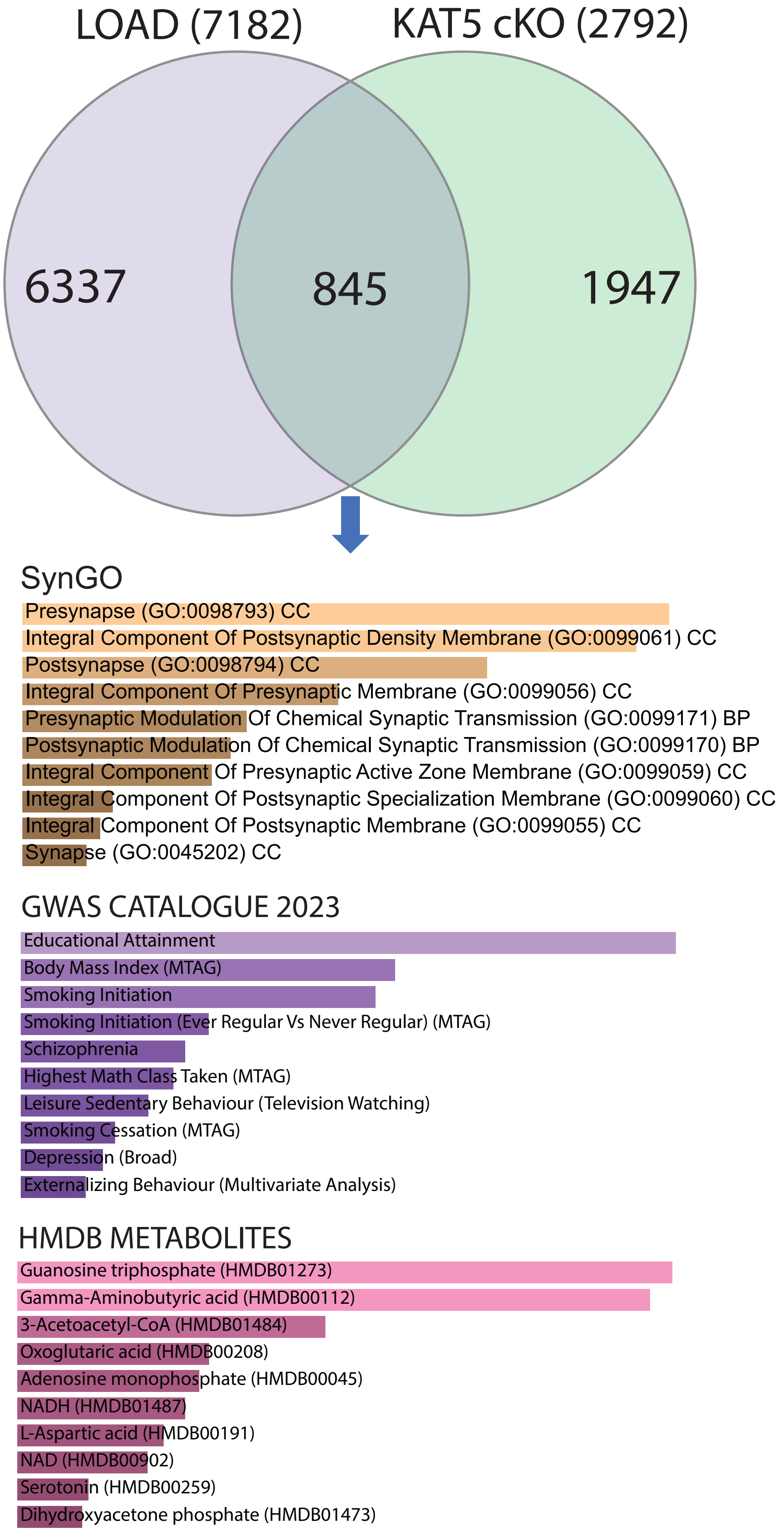
LOAD (7182)
KAT5 cKO (2792)
6337
845
1947
SynGO
Presynapse (GO:0098793) CC
Integral Component Of Postsynaptic Density Membrane (GO:0099061) CC
Postsynapse (GO:0098794) CC
Integral Component Of Presynaptic Membrane (GO:0099056) CC
Presynaptic Modulation Of Chemical Synaptic Transmission (GO:0099171) BP
Postsynaptic Modulation Of Chemical Synaptic Transmission (GO:0099170) BP
Integral Component Of Presynaptic Active Zone Membrane (GO:0099059) CC
Integral Component Of Postsynaptic Specialization Membrane (GO:0099060) CC
Integral Component Of Postsynaptic Membrane (GO:0099055) CC
Synapse (GO:0045202) CC
GWAS CATALOGUE 2023
Educational Attainment
Body Mass Index (MTAG)
Smoking Initiation
Smoking Initiation (Ever Regular Vs Never Regular) (MTAG)
Schizophrenia
Highest Math Class Taken (MTAG)
Leisure Sedentary Behaviour (Television Watching)
Smoking Cessation (MTAG)
Depression (Broad)
Externalizing Behaviour (Multivariate Analysis)
HMDB METABOLITES
Guanosine triphosphate (HMDB01273)
Gamma-Aminobutyric acid (HMDB00112)
3-Acetoacetyl-CoA (HMDB01484)
Oxoglutaric acid (HMDB00208)
Adenosine monophosphate (HMDB00045)
NADH (HMDB01487)
L-Aspartic acid (HMDB00191)
NAD (HMDB00902)
Serotonin (HMDB00259)
Dihydroxyacetone phosphate (HMDB01473)

Figure Legend S4. Additional Analyses of the intersection of the LOAD and Kat5 cKO down-regulated gene sets.

The down-regulated LOAD genes from the TREAT-AD bioinformatics pipeline analysis are employed in conjunction with the 0.2 or greater down-regulated Kat5 cKO genes, resulting in the previously discussed 845 genes common to both gene sets. These are further analysis by gene set enrichment analysis against the GTEX aging signatures, the GWAS Catalogue gene variant-trait linkages, and the human metabolite database (HMDB). The GWAS catalogue shows the greatest overlap with educational attainment, suggesting a link between the Kat5 cKO regulated genes, those that decrement in AD, and genes enhanced by educational achievement. Interestingly, the HMDB signatures that are most highly shared represent elements of core metabolism and neurotransmitters synthesis, such as GABA and Serotonin. SynGO enrichments were included to show synaptically filtered process (SynGO). All colored bars are significant at $p<0.05$, with shorter bars denoting decreased p-values and darker colors representing decreasing enrichment scores. Exact enrichment values and scores are contained within Supplementary table 2.

Figure S5

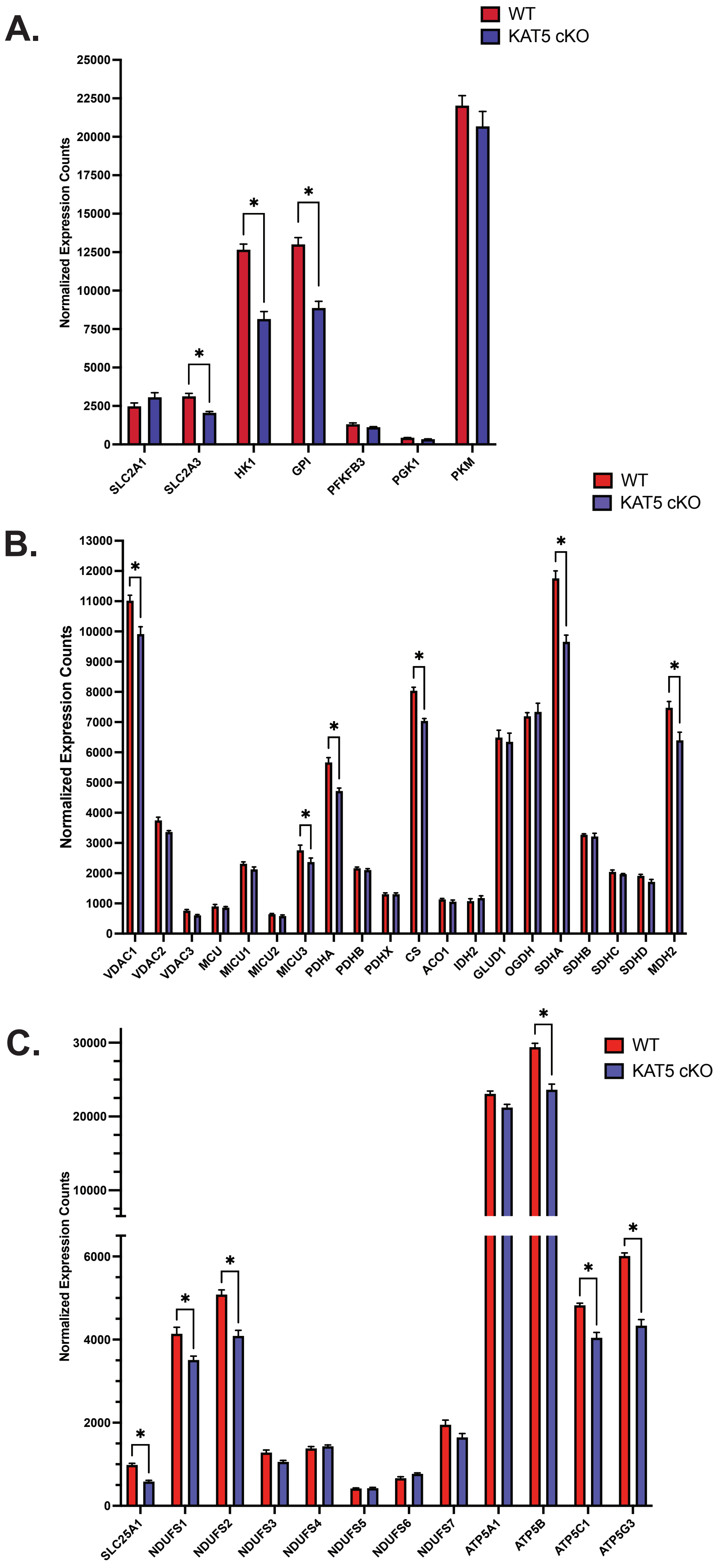
A.
WT
KAT5 cKO
Normalized Expression Counts
25000
22500
20000
17500
15000
12500
10000
7500
5000
2500
0
SLC2A1
SLC2A3
HK1
GPI
PFKFB3
PGK1
PKM
B.
WT
KAT5 cKO
Normalized Expression Counts
13000
12000
11000
10000
9000
8000
7000
6000
5000
4000
3000
2000
1000
0
VDAC1
VDAC2
VDAC3
MCU
MICU1
MICU2
MICU3
PDHA
PDHB
PDHX
CS
ACO1
IDH2
GLUD1
OGDH
SDHA
SDHB
SDHC
SDHD
MDH2
C.
WT
KAT5 cKO
Normalized Expression Counts
30000
20000
10000
6000
4000
2000
0
SLC25A1
NDUFS1
NDUFS2
NDUFS3
NDUFS4
NDUFS5
NDUFS6
NDUFS7
ATP5A1
ATP5B
ATP5C1
ATP5G3

Figure Legend S5. Analysis of the glycolysis, tricarboxylic acid (TCA) cycle, and electron transport chain (ETC) genes in the Kat5 cKO vs control.

The six animals per genotype employed in the Kat5 DEG comparison study were investigated for specific differences in defined genes associated with stages of metabolism. The core enzymes in the glycolytic pathway are compared in (A) including an examination of glucose uptake from the neurovasculature by astrocytes, the neuronal glucose uptake from the astrocytic endfeet, and the processing to pyruvate through the core glycolytic enzymes. The biological process analyzed is visually depicted to the right side of the graphs. The neuron specific glucose transporter SLC2A3 is significantly down-regulated within the Kat5 cKO model compared to wild-type. Additionally, HK1 and GPI, the initial enzymatic steps within glycolysis, are also downregulated. The enzymes involved in pyruvate transport and conversion into acetyl-CoA and subsequent processing through the TCA cycle is depicted in (B) with graphical representation of both calcium import factors and the TCA cycle represented to the right. There are isolated decrements in both transport and TCA cycle genes—specifically PDHA, CS, SDHA and MDH2—within the Kat5 cKO model. The transcript levels for the core enzyme involved in conversion of NADH and FADH2 into ATP through the ETC are examined in (C). There are small decreases distributed across complex I and complex V observed, as noted by the asterix ($padj<0.05$).

Figure S6

A.

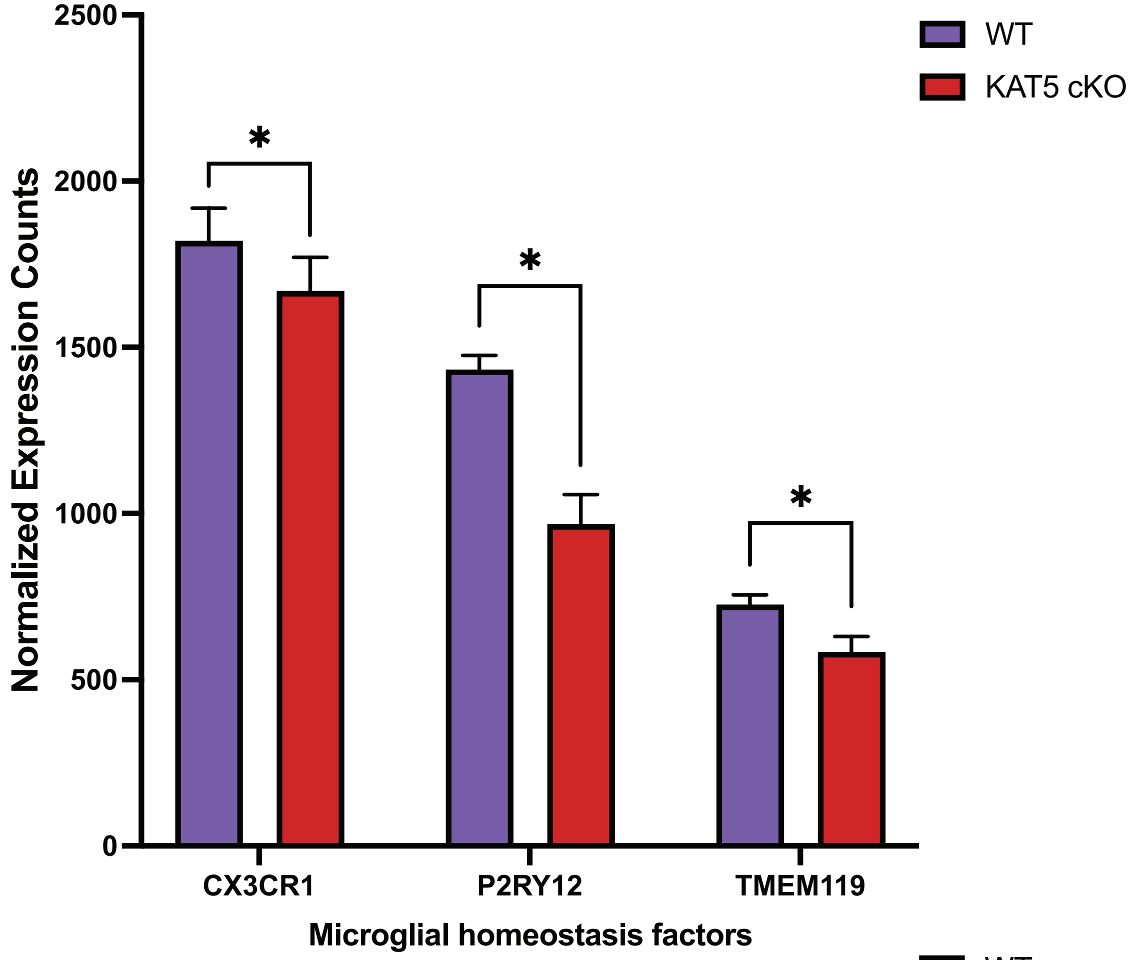

WT
KAT5 cKO
2500
2000
1500
1000
500
0
Normalized Expression Counts
*
*
*
CX3CR1
P2RY12
TMEM119
Microglial homeostasis factors

B.

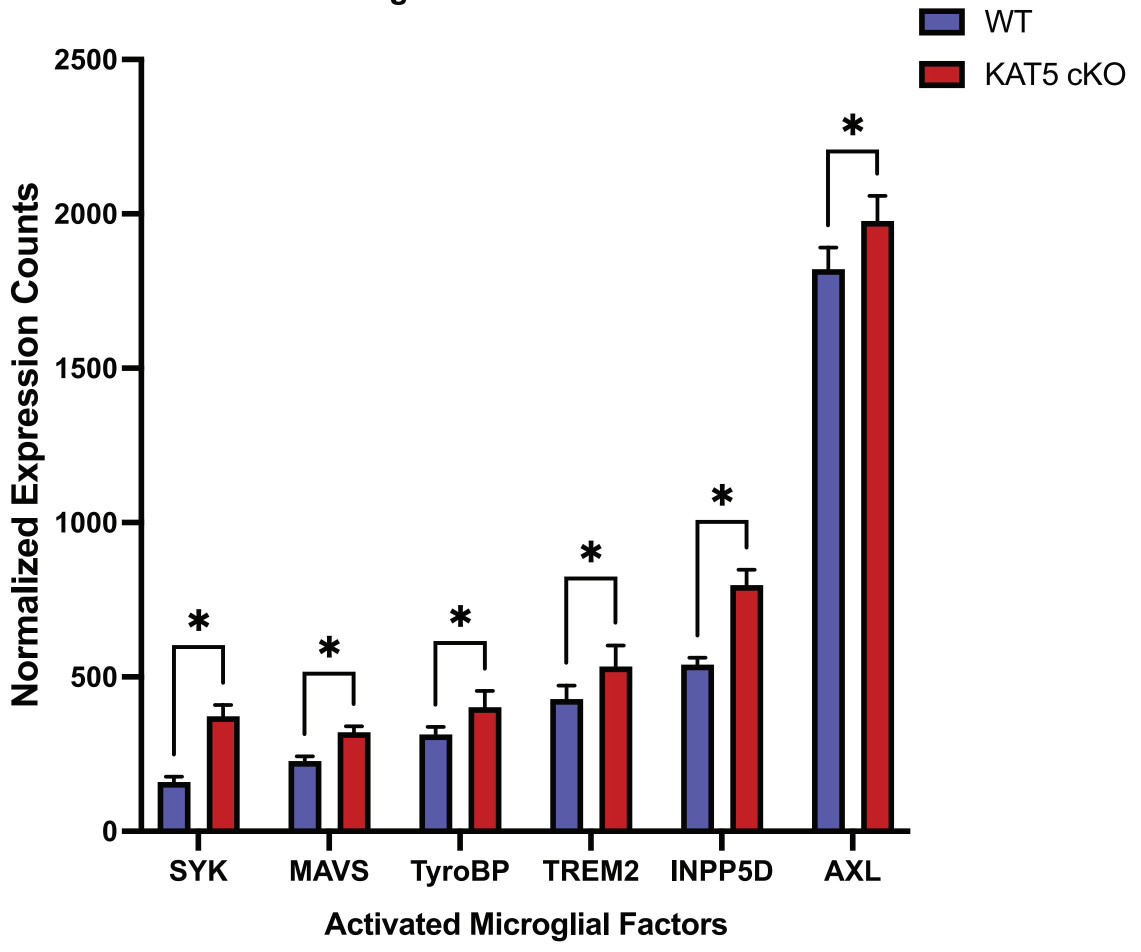

WT
KAT5 cKO
2500
2000
1500
1000
500
0
Normalized Expression Counts
*
*
*
*
*
*
SYK
MAVS
TyroBP
TREM2
INPP5D
AXL
Activated Microglial Factors

Figure Legend S6. Kat5 cKO downregulates homeostatic microglial factors and upregulates activated microglial markers.

The phenotype of the Kat5 cKO involves progressive reactive gliosis, so we examined the levels of known homeostatic microglial markers (A) and activated microglial markers (B) within the transcriptomic signature occurring prior to pathological encroachment through a comparison of the six Kat5 cKO mice compared to the wild-type controls. The Kat5 cKO mice had small but statistically significant decreases in CX3CR1, P2RY12 and TMEM119 as shown in (A) with the asterix representing significance at the padj<0.05. Conversely, the activated markers SYK, MAVS, TYROBP, TREM2, INPP5D and AXL are all upregulated by small but significant margins (B), with the asterix bar comparison denoting padj<0.05. The overall scope of the change is small, the transcriptomic signature was obtained from mice two weeks after induced gene ablation.

Figure S7

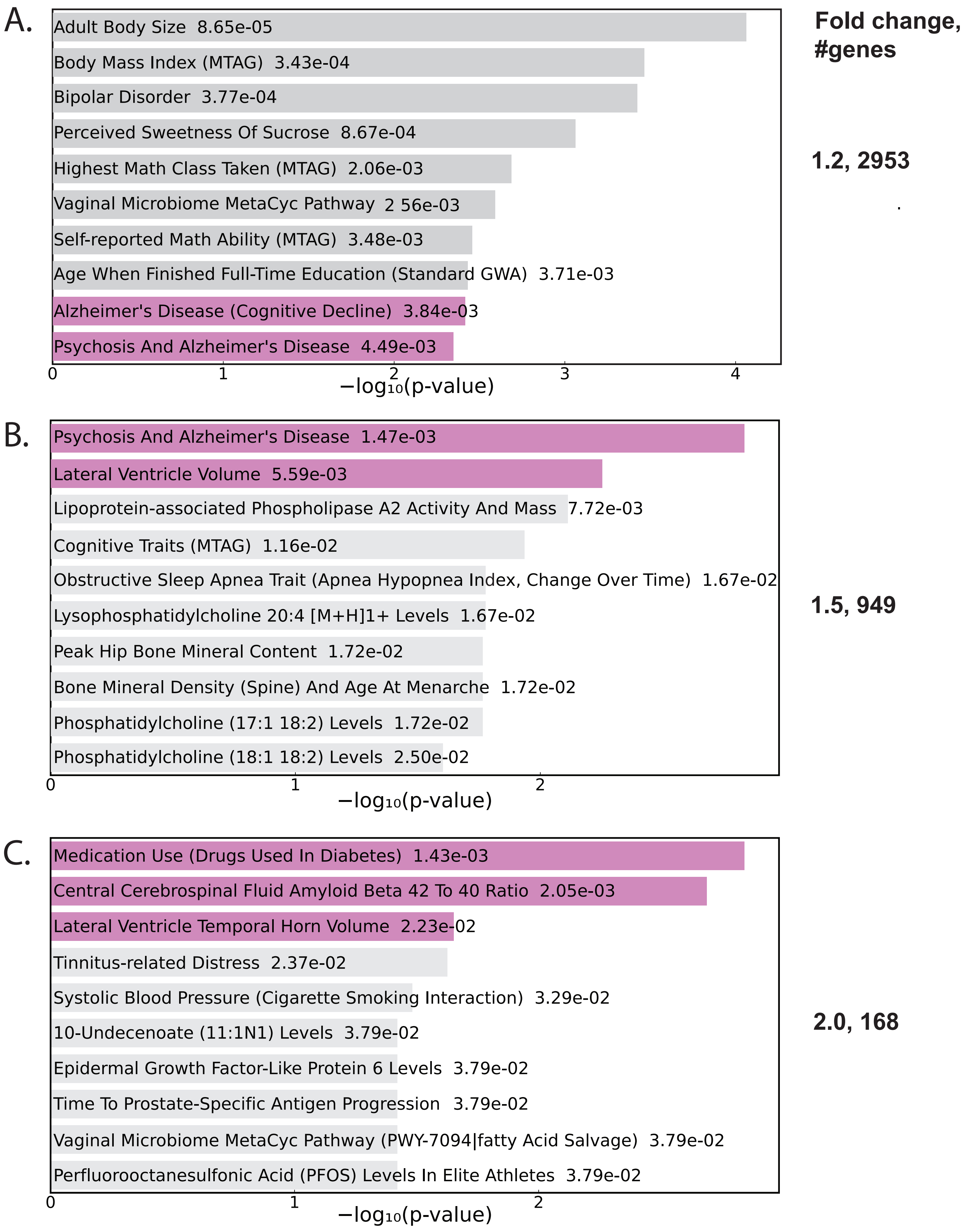
A.
Fold change, #genes
Adult Body Size 8.65e-05
Body Mass Index (MTAG) 3.43e-04
Bipolar Disorder 3.77e-04
Perceived Sweetness Of Sucrose 8.67e-04
Highest Math Class Taken (MTAG) 2.06e-03
Vaginal Microbiome MetaCyc Pathway 2 56e-03
Self-reported Math Ability (MTAG) 3.48e-03
Age When Finished Full-Time Education (Standard GWA) 3.71e-03
Alzheimer's Disease (Cognitive Decline) 3.84e-03
Psychosis And Alzheimer's Disease 4.49e-03
0
1
2
3
4
−log10(p-value)
1.2, 2953
B.
Psychosis And Alzheimer's Disease 1.47e-03
Lateral Ventricle Volume 5.59e-03
Lipoprotein-associated Phospholipase A2 Activity And Mass 7.72e-03
Cognitive Traits (MTAG) 1.16e-02
Obstructive Sleep Apnea Trait (Apnea Hypopnea Index, Change Over Time) 1.67e-02
Lysophosphatidylcholine 20:4 [M+H]1+ Levels 1.67e-02
Peak Hip Bone Mineral Content 1.72e-02
Bone Mineral Density (Spine) And Age At Menarche 1.72e-02
Phosphatidylcholine (17:1 18:2) Levels 1.72e-02
Phosphatidylcholine (18:1 18:2) Levels 2.50e-02
0
1
2
−log10(p-value)
1.5, 949
C.
Medication Use (Drugs Used In Diabetes) 1.43e-03
Central Cerebrospinal Fluid Amyloid Beta 42 To 40 Ratio 2.05e-03
Lateral Ventricle Temporal Horn Volume 2.23e-02
Tinnitus-related Distress 2.37e-02
Systolic Blood Pressure (Cigarette Smoking Interaction) 3.29e-02
10-Undecenoate (11:1N1) Levels 3.79e-02
Epidermal Growth Factor-Like Protein 6 Levels 3.79e-02
Time To Prostate-Specific Antigen Progression 3.79e-02
Vaginal Microbiome MetaCyc Pathway (PWY-7094|fatty Acid Salvage) 3.79e-02
Perfluorooctanesulfonic Acid (PFOS) Levels In Elite Athletes 3.79e-02
0
1
2
−log10(p-value)
2.0, 168

Figure S7. Genetic Trait Mapping of Kat5 Downregulated Genes.

The GWAS 2023 Catalogue was trait mapped using three different stringencies of the Kat5 downregulated genes: mild (1.2 fold), moderate (1.5 fold) and strong (2 fold). The more permissive DEG filtering result in different size gene sets ranging from 2953 (mild), 959 (moderate) and 169 (strong). The Kat5 DEGs were matched to the GWAS catalogue genes and the traits to which variants demonstrated linkage. In all three stringencies of filtration, the Kat5 cKO attenuated DEGs map onto AD, or AD-related traits. In the mild condition, AD (Cognitive Decline) and Psychosis and Alzheimer's Disease are observed. In the middle analysis, the top two enriched traits are Psychosis and Alzheimer's Disease and Lateral Ventricle Volume (a physiological biomarker of AD). In the most stringent analysis shown in the bottom panel, we observe enriched trait linkage with Medication Usage (Diabetes), Cerebral Spinal Fluid Amyloid 42 to 40 Ratio, and Lateral Ventricle and Temporal Horn Volume.

Figure S8

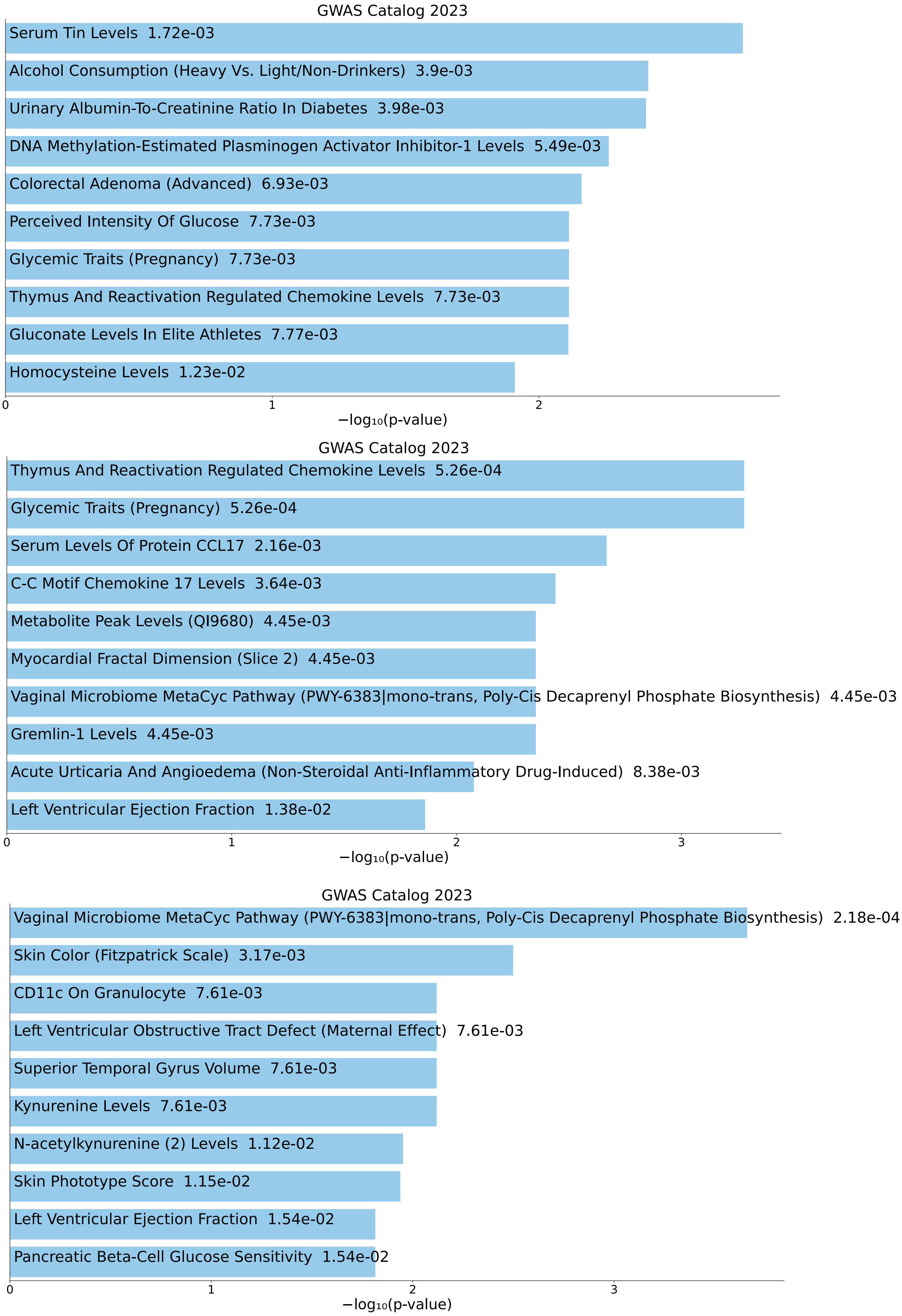
GWAS Catalog 2023
Serum Tin Levels 1.72e-03
Alcohol Consumption (Heavy Vs. Light/Non-Drinkers) 3.9e-03
Urinary Albumin-To-Creatinine Ratio In Diabetes 3.98e-03
DNA Methylation-Estimated Plasminogen Activator Inhibitor-1 Levels 5.49e-03
Colorectal Adenoma (Advanced) 6.93e-03
Perceived Intensity Of Glucose 7.73e-03
Glycemic Traits (Pregnancy) 7.73e-03
Thymus And Reactivation Regulated Chemokine Levels 7.73e-03
Gluconate Levels In Elite Athletes 7.77e-03
Homocysteine Levels 1.23e-02
0
1
2
−log10(p-value)
GWAS Catalog 2023
Thymus And Reactivation Regulated Chemokine Levels 5.26e-04
Glycemic Traits (Pregnancy) 5.26e-04
Serum Levels Of Protein CCL17 2.16e-03
C-C Motif Chemokine 17 Levels 3.64e-03
Metabolite Peak Levels (QI9680) 4.45e-03
Myocardial Fractal Dimension (Slice 2) 4.45e-03
Vaginal Microbiome MetaCyc Pathway (PWY-6383|mono-trans, Poly-Cis Decaprenyl Phosphate Biosynthesis) 4.45e-03
Gremlin-1 Levels 4.45e-03
Acute Urticaria And Angioedema (Non-Steroidal Anti-Inflammatory Drug-Induced) 8.38e-03
Left Ventricular Ejection Fraction 1.38e-02
0
1
2
3
−log10(p-value)
GWAS Catalog 2023
Vaginal Microbiome MetaCyc Pathway (PWY-6383|mono-trans, Poly-Cis Decaprenyl Phosphate Biosynthesis) 2.18e-04
Skin Color (Fitzpatrick Scale) 3.17e-03
CD11c On Granulocyte 7.61e-03
Left Ventricular Obstructive Tract Defect (Maternal Effect) 7.61e-03
Superior Temporal Gyrus Volume 7.61e-03
Kynurenine Levels 7.61e-03
N-acetylkynurenine (2) Levels 1.12e-02
Skin Phototype Score 1.15e-02
Left Ventricular Ejection Fraction 1.54e-02
Pancreatic Beta-Cell Glucose Sensitivity 1.54e-02
0
1
2
3
−log10(p-value)

Figure Legend S8. The GWAS Catalogue gene variant-trait linkages within the upregulated geneset of the Kat5 cKO mice.

The comparison of the 1.2 fold (3229 genes), 1.5 fold (1592), and 2 fold (568) upregulated genes statistically significant at $p<0.05$ post FDR correction were examined for gene variant associations with identified traits within the GWAS Catalogue studies. This comparison is the converse analysis as performed in Figure 9 examining the downregulated genes. The overall significance levels are lower in the upregulated genes than observed in the downregulated gene set, with less evidence of clear specific genetic associations. There does appear to be an association with some cardiac and vascular function, as homocysteine levels are observed within the 1.2 fold upregulated gene set trait associations, myocardial fractal dimension, acute urticaria and angioedema and left ventricular ejection fraction are enriched within the 1.5 fold upregulated gene set and left ventricular obstructive tract defect and left ventricular ejection fraction are weakly associated with the 2.0 fold enriched set. Overall, the patterns of gene variant-trait association in the upregulated genes appear less identifiable and weaker than in the downregulated gene set.

Figure S9

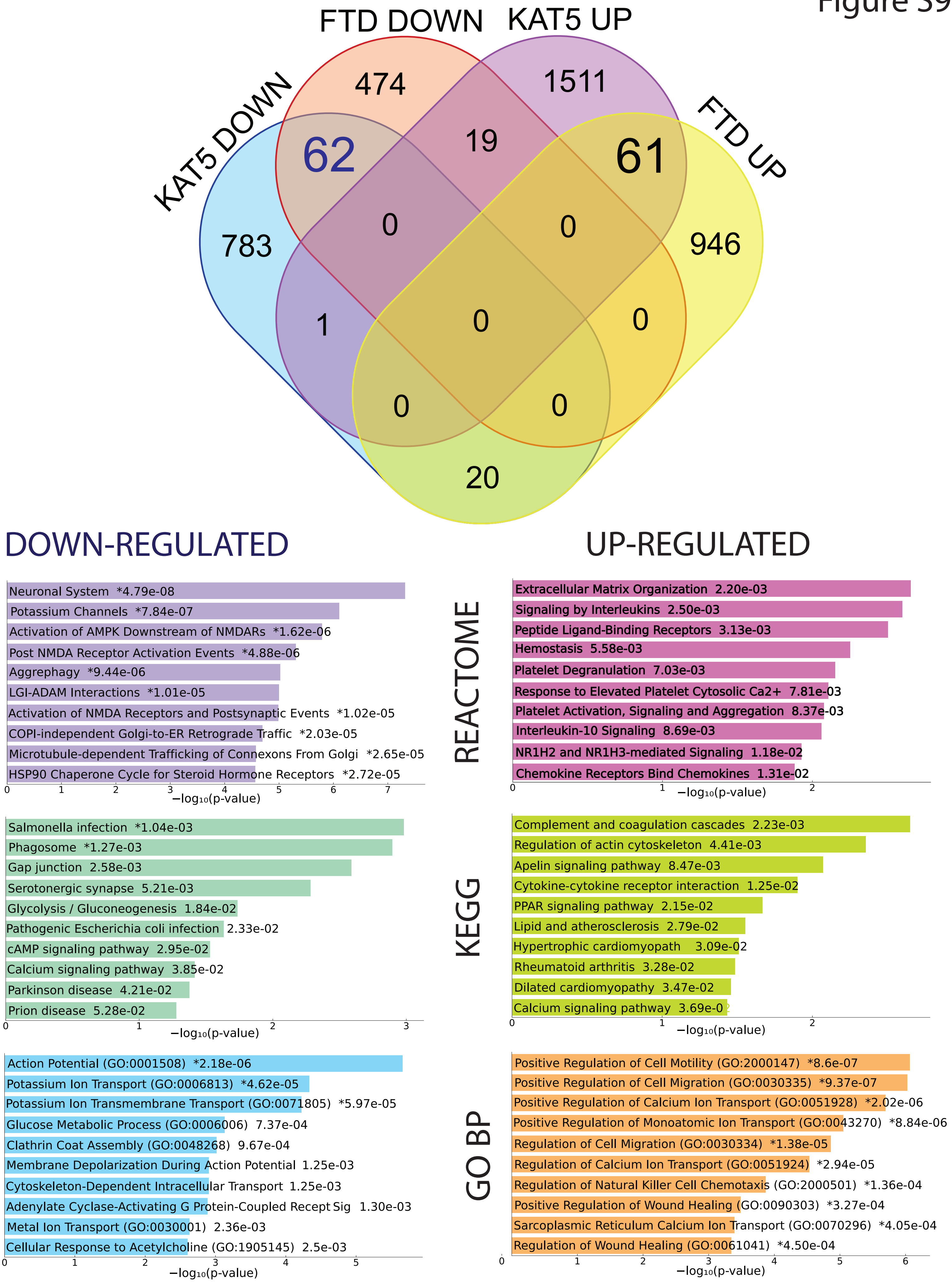
FTD DOWN
KAT5 UP
KAT5 DOWN
FTD UP
474
1511
62
19
61
783
0
0
946
1
0
0
0
0
20
DOWN-REGULATED
UP-REGULATED
REACTOME
KEGG
GO BP
Neuronal System *4.79e-08
Potassium Channels *7.84e-07
Activation of AMPK Downstream of NMDARs *1.62e-06
Post NMDA Receptor Activation Events *4.88e-06
Aggrephagy *9.44e-06
LGI-ADAM Interactions *1.01e-05
Activation of NMDA Receptors and Postsynaptic Events *1.02e-05
COPI-independent Golgi-to-ER Retrograde Traffic *2.03e-05
Microtubule-dependent Trafficking of Connexons From Golgi *2.65e-05
HSP90 Chaperone Cycle for Steroid Hormone Receptors *2.72e-05
−log10(p-value)
Extracellular Matrix Organization 2.20e-03
Signaling by Interleukins 2.50e-03
Peptide Ligand-Binding Receptors 3.13e-03
Hemostasis 5.58e-03
Platelet Degranulation 7.03e-03
Response to Elevated Platelet Cytosolic Ca2+ 7.81e-03
Platelet Activation, Signaling and Aggregation 8.37e-03
Interleukin-10 Signaling 8.69e-03
NR1H2 and NR1H3-mediated Signaling 1.18e-02
Chemokine Receptors Bind Chemokines 1.31e-02
−log10(p-value)
Salmonella infection *1.04e-03
Phagosome *1.27e-03
Gap junction 2.58e-03
Serotonergic synapse 5.21e-03
Glycolysis / Gluconeogenesis 1.84e-02
Pathogenic Escherichia coli infection 2.33e-02
cAMP signaling pathway 2.95e-02
Calcium signaling pathway 3.85e-02
Parkinson disease 4.21e-02
Prion disease 5.28e-02
−log10(p-value)
Complement and coagulation cascades 2.23e-03
Regulation of actin cytoskeleton 4.41e-03
Apelin signaling pathway 8.47e-03
Cytokine-cytokine receptor interaction 1.25e-02
PPAR signaling pathway 2.15e-02
Lipid and atherosclerosis 2.79e-02
Hypertrophic cardiomyopath 3.09e-02
Rheumatoid arthritis 3.28e-02
Dilated cardiomyopathy 3.47e-02
Calcium signaling pathway 3.69e-02
−log10(p-value)
Action Potential (GO:0001508) *2.18e-06
Potassium Ion Transport (GO:0006813) *4.62e-05
Potassium Ion Transmembrane Transport (GO:0071805) *5.97e-05
Glucose Metabolic Process (GO:0006006) 7.37e-04
Clathrin Coat Assembly (GO:0048268) 9.67e-04
Membrane Depolarization During Action Potential 1.25e-03
Cytoskeleton-Dependent Intracellular Transport 1.25e-03
Adenylate Cyclase-Activating G Protein-Coupled Recept Sig 1.30e-03
Metal Ion Transport (GO:0030001) 2.36e-03
Cellular Response to Acetylcholine (GO:1905145) 2.5e-03
−log10(p-value)
Positive Regulation of Cell Motility (GO:2000147) *8.6e-07
Positive Regulation of Cell Migration (GO:0030335) *9.37e-07
Positive Regulation of Calcium Ion Transport (GO:0051928) *2.02e-06
Positive Regulation of Monoatomic Ion Transport (GO:0043270) *8.84e-06
Regulation of Cell Migration (GO:0030334) *1.38e-05
Regulation of Calcium Ion Transport (GO:0051924) *2.94e-05
Regulation of Natural Killer Cell Chemotaxis (GO:2000501) *1.36e-04
Positive Regulation of Wound Healing (GO:0090303) *3.27e-04
Sarcoplasmic Reticulum Calcium Ion Transport (GO:0070296) *4.05e-04
Regulation of Wound Healing (GO:0061041) *4.50e-04
−log10(p-value)

Figure Legend Supplemental 9. Kat5 cKO and MAPT FTD differential expression alignment.

The differential expression profile from the cKO Kat5 mouse were compared to the differential expression profile for the human frontotemporal dementia patients examined within the Risk and Modifying Factors in Frontotemporal Dementia (RIMOD-FTD) consortium. We specifically compared the MAPT linked FTD patients to the Kat5 cKO data to assess the most pathogenically similar dataset to AD. In all cases, there is 10-15% overlap in specific differentially expressed genes in up- and down-regulated gene sets. The degree of overlap is higher in the down-regulated FTD gene set. The overall biological pattern of enrichment in the up- and down-regulated gene set intersection is characterized by REACTOME and KEGG pathways in the top two plots (left: down-regulated; right: up-regulated). The GO biological process enrichment is shown on the bottom plots. The significance is corrected for multiple testing.

Figure S10

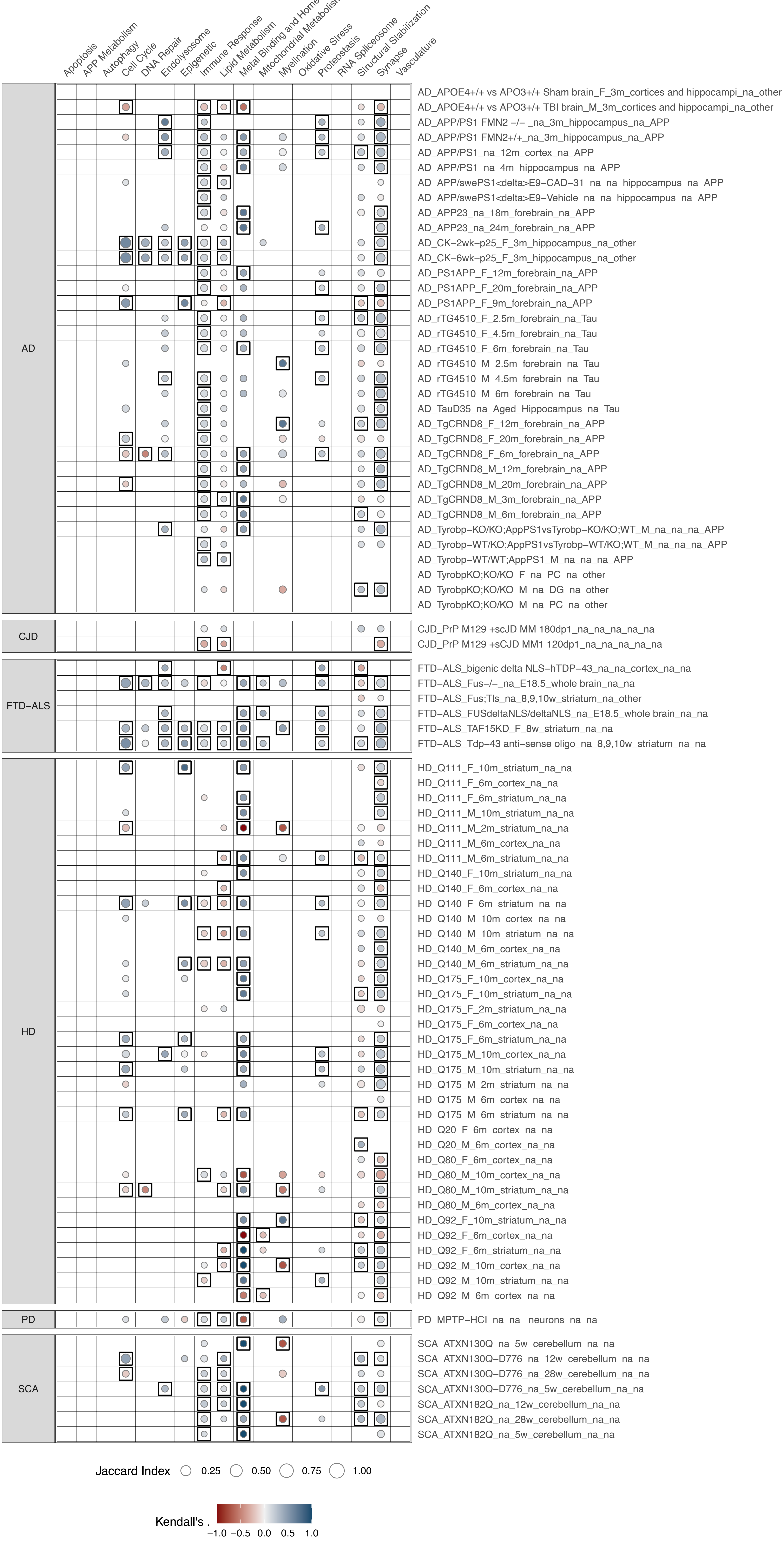

Apoptosis
APP Metabolism
Autophagy
Cell Cycle
DNA Repair
Endolysosome
Epigenetic
Immune Response
Lipid Metabolism
Metal Binding and Homeostasis
Mitochondrial Metabolism
Myelination
Oxidative Stress
Proteostasis
RNA Spliceosome
Structural Stabilization
Synapse
Vasculature
AD
AD_APOE4+/+ vs APO3+/+ Sham brain_F_3m_cortices and hippocampi_na_other
AD_APOE4+/+ vs APO3+/+ TBI brain_M_3m_cortices and hippocampi_na_other
AD_APP/PS1 FMN2 −/− _na_3m_hippocampus_na_APP
AD_APP/PS1 FMN2+/+_na_3m_hippocampus_na_APP
AD_APP/PS1_na_12m_cortex_na_APP
AD_APP/PS1_na_4m_hippocampus_na_APP
AD_APP/swePS1<delta>E9−CAD−31_na_na_hippocampus_na_APP
AD_APP/swePS1<delta>E9−Vehicle_na_na_hippocampus_na_APP
AD_APP23_na_18m_forebrain_na_APP
AD_APP23_na_24m_forebrain_na_APP
AD_CK−2wk−p25_F_3m_hippocampus_na_other
AD_CK−6wk−p25_F_3m_hippocampus_na_other
AD_PS1APP_F_12m_forebrain_na_APP
AD_PS1APP_F_20m_forebrain_na_APP
AD_PS1APP_F_9m_forebrain_na_APP
AD_rTG4510_F_2.5m_forebrain_na_Tau
AD_rTG4510_F_4.5m_forebrain_na_Tau
AD_rTG4510_F_6m_forebrain_na_Tau
AD_rTG4510_M_2.5m_forebrain_na_Tau
AD_rTG4510_M_4.5m_forebrain_na_Tau
AD_rTG4510_M_6m_forebrain_na_Tau
AD_TauD35_na_Aged_Hippocampus_na_Tau
AD_TgCRND8_F_12m_forebrain_na_APP
AD_TgCRND8_F_20m_forebrain_na_APP
AD_TgCRND8_F_6m_forebrain_na_APP
AD_TgCRND8_M_12m_forebrain_na_APP
AD_TgCRND8_M_20m_forebrain_na_APP
AD_TgCRND8_M_3m_forebrain_na_APP
AD_TgCRND8_M_6m_forebrain_na_APP
AD_Tyrobp−KO/KO;AppPS1vsTyrobp−KO/KO;WT_M_na_na_na_APP
AD_Tyrobp−WT/KO;AppPS1vsTyrobp−WT/KO;WT_M_na_na_na_APP
AD_Tyrobp−WT/WT;AppPS1_M_na_na_na_APP
AD_TyrobpKO;KO/KO_F_na_PC_na_other
AD_TyrobpKO;KO/KO_M_na_DG_na_other
AD_TyrobpKO;KO/KO_M_na_PC_na_other
CJD
CJD_PrP M129 +scJD MM 180dp1_na_na_na_na_na
CJD_PrP M129 +sCJD MM1 120dp1_na_na_na_na_na
FTD−ALS
FTD−ALS_bigenic delta NLS−hTDP−43_na_na_cortex_na_na
FTD−ALS_Fus−/−_na_E18.5_whole brain_na_na
FTD−ALS_Fus;Tls_na_8,9,10w_striatum_na_other
FTD−ALS_FUSdeltaNLS/deltaNLS_na_E18.5_whole brain_na_na
FTD−ALS_TAF15KD_F_8w_striatum_na_na
FTD−ALS_Tdp−43 anti−sense oligo_na_8,9,10w_striatum_na_na
HD
HD_Q111_F_10m_striatum_na_na
HD_Q111_F_6m_cortex_na_na
HD_Q111_F_6m_striatum_na_na
HD_Q111_M_10m_striatum_na_na
HD_Q111_M_2m_striatum_na_na
HD_Q111_M_6m_cortex_na_na
HD_Q111_M_6m_striatum_na_na
HD_Q140_F_10m_striatum_na_na
HD_Q140_F_6m_cortex_na_na
HD_Q140_F_6m_striatum_na_na
HD_Q140_M_10m_cortex_na_na
HD_Q140_M_10m_striatum_na_na
HD_Q140_M_6m_cortex_na_na
HD_Q140_M_6m_striatum_na_na
HD_Q175_F_10m_cortex_na_na
HD_Q175_F_10m_striatum_na_na
HD_Q175_F_2m_striatum_na_na
HD_Q175_F_6m_cortex_na_na
HD_Q175_F_6m_striatum_na_na
HD_Q175_M_10m_cortex_na_na
HD_Q175_M_10m_striatum_na_na
HD_Q175_M_2m_striatum_na_na
HD_Q175_M_6m_cortex_na_na
HD_Q175_M_6m_striatum_na_na
HD_Q20_F_6m_cortex_na_na
HD_Q20_M_6m_cortex_na_na
HD_Q80_F_6m_cortex_na_na
HD_Q80_M_10m_cortex_na_na
HD_Q80_M_10m_striatum_na_na
HD_Q80_M_6m_cortex_na_na
HD_Q92_F_10m_striatum_na_na
HD_Q92_F_6m_cortex_na_na
HD_Q92_F_6m_striatum_na_na
HD_Q92_M_10m_cortex_na_na
HD_Q92_M_10m_striatum_na_na
HD_Q92_M_6m_cortex_na_na
PD
PD_MPTP−HCl_na_na_ neurons_na_na
SCA
SCA_ATXN130Q_na_5w_cerebellum_na_na
SCA_ATXN130Q−D776_na_12w_cerebellum_na_na
SCA_ATXN130Q−D776_na_28w_cerebellum_na_na
SCA_ATXN130Q−D776_na_5w_cerebellum_na_na
SCA_ATXN182Q_na_12w_cerebellum_na_na
SCA_ATXN182Q_na_28w_cerebellum_na_na
SCA_ATXN182Q_na_5w_cerebellum_na_na
Jaccard Index
0.25
0.50
0.75
1.00
Kendall's .
−1.0
−0.5
0.0
0.5
1.0

Figure S10. Comparison of Neurodegenerative Disease Mouse Models to Kat5 cKO.

Numerous neurodegenerative mouse models were comparatively analyzed for their correlative enrichment of biological domains based on GO term GSEA of the differentially expressed genes. The mouse model datasets have been previously described in the analysis by Wan et al. The top set of mouse models are enumerated on the right vertical axis, grouped by disease state shown on the left vertical box. We examined multiple mouse models associated with AD, CJD, FTD-ALS, HD, PD, and SCA. Positive term based enrichment correlation is shown in blue, while a negative correlation is shown in red. At a biological domain level there is significant term correlation across mouse models and disease-states within multiple domains, including synapse, immune response, and endolysosome. These correlations show the similarity of enrichment with the Kat5 cKO mouse model, not direction of expression change. Synapse enrichment appears relatively consistent across AD, CJD, FTD-ALS, HD and SCA, with some specific exceptions including in one CJD model and a couple of the HD models. Immune response is consistent across AD models, but more variable within other disease state models.

Figure S11

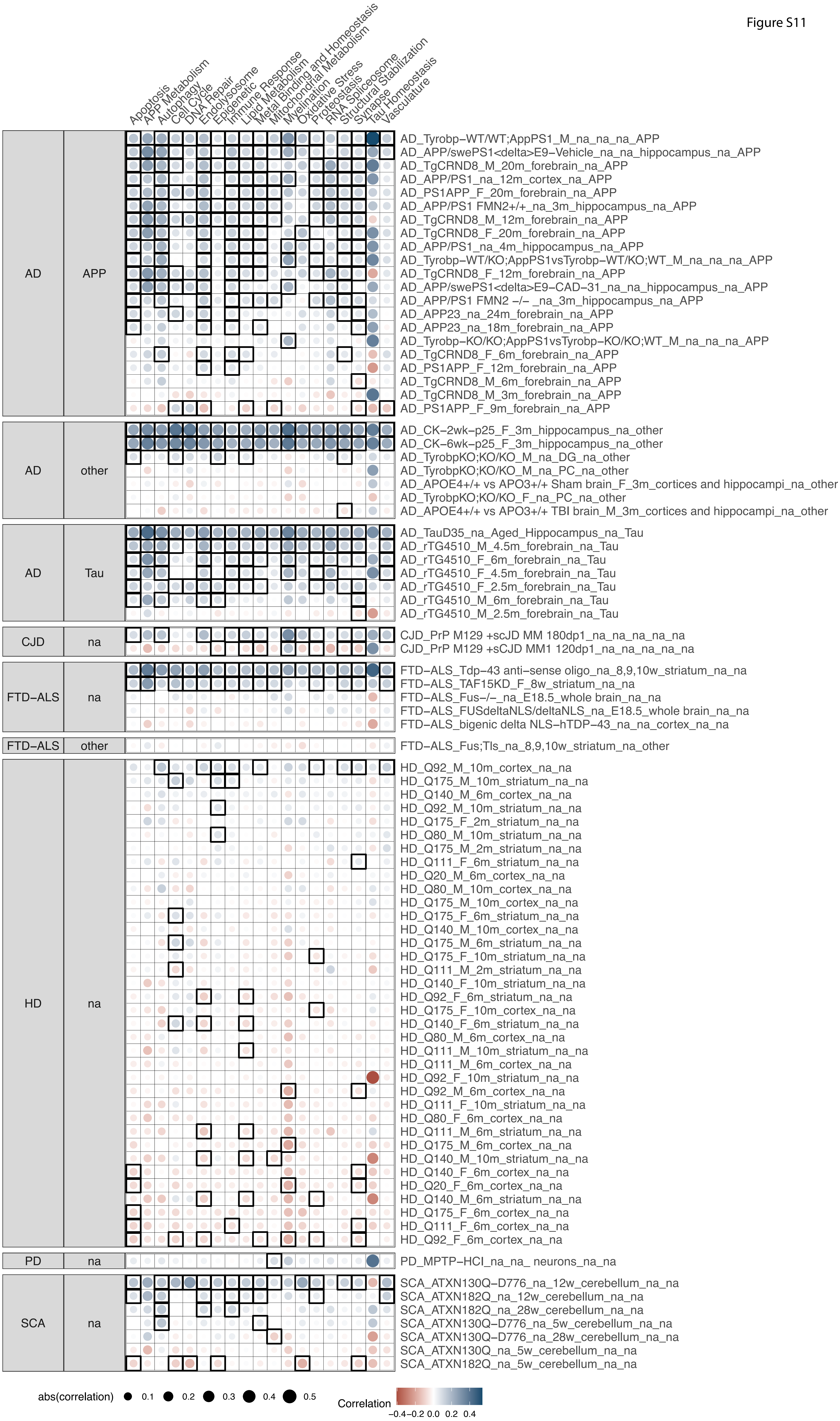

Apoptosis
APP Metabolism
Autophagy
Cell Cycle
DNA Repair
Endolysosome
Epigenetic
Immune Response
Lipid Metabolism
Metal Binding and Homeostasis
Mitochondrial Metabolism
Myelination
Oxidative Stress
Proteostasis
RNA Spliceosome
Structural Stabilization
Synapse
Tau Homeostasis
Vasculature
AD
APP
AD_Tyrobp−WT/WT;AppPS1_M_na_na_na_APP
AD_APP/swePS1<delta>E9−Vehicle_na_na_hippocampus_na_APP
AD_TgCRND8_M_20m_forebrain_na_APP
AD_APP/PS1_na_12m_cortex_na_APP
AD_PS1APP_F_20m_forebrain_na_APP
AD_APP/PS1 FMN2+/+_na_3m_hippocampus_na_APP
AD_TgCRND8_M_12m_forebrain_na_APP
AD_TgCRND8_F_20m_forebrain_na_APP
AD_APP/PS1_na_4m_hippocampus_na_APP
AD_Tyrobp−WT/KO;AppPS1vsTyrobp−WT/KO;WT_M_na_na_na_APP
AD_TgCRND8_F_12m_forebrain_na_APP
AD_APP/swePS1<delta>E9−CAD−31_na_na_hippocampus_na_APP
AD_APP/PS1 FMN2 −/− _na_3m_hippocampus_na_APP
AD_APP23_na_24m_forebrain_na_APP
AD_APP23_na_18m_forebrain_na_APP
AD_Tyrobp−KO/KO;AppPS1vsTyrobp−KO/KO;WT_M_na_na_na_APP
AD_TgCRND8_F_6m_forebrain_na_APP
AD_PS1APP_F_12m_forebrain_na_APP
AD_TgCRND8_M_6m_forebrain_na_APP
AD_TgCRND8_M_3m_forebrain_na_APP
AD_PS1APP_F_9m_forebrain_na_APP
AD
other
AD_CK−2wk−p25_F_3m_hippocampus_na_other
AD_CK−6wk−p25_F_3m_hippocampus_na_other
AD_TyrobpKO;KO/KO_M_na_DG_na_other
AD_TyrobpKO;KO/KO_M_na_PC_na_other
AD_APOE4+/+ vs APO3+/+ Sham brain_F_3m_cortices and hippocampi_na_other
AD_TyrobpKO;KO/KO_F_na_PC_na_other
AD_APOE4+/+ vs APO3+/+ TBI brain_M_3m_cortices and hippocampi_na_other
AD
Tau
AD_TauD35_na_Aged_Hippocampus_na_Tau
AD_rTG4510_M_4.5m_forebrain_na_Tau
AD_rTG4510_F_6m_forebrain_na_Tau
AD_rTG4510_F_4.5m_forebrain_na_Tau
AD_rTG4510_F_2.5m_forebrain_na_Tau
AD_rTG4510_M_6m_forebrain_na_Tau
AD_rTG4510_M_2.5m_forebrain_na_Tau
CJD
na
CJD_PrP M129 +scJD MM 180dp1_na_na_na_na_na
CJD_PrP M129 +sCJD MM1 120dp1_na_na_na_na_na
FTD−ALS
na
FTD−ALS_Tdp−43 anti−sense oligo_na_8,9,10w_striatum_na_na
FTD−ALS_TAF15KD_F_8w_striatum_na_na
FTD−ALS_Fus−/−_na_E18.5_whole brain_na_na
FTD−ALS_FUSdeltaNLS/deltaNLS_na_E18.5_whole brain_na_na
FTD−ALS_bigenic delta NLS−hTDP−43_na_na_cortex_na_na
FTD−ALS
other
FTD−ALS_Fus;Tls_na_8,9,10w_striatum_na_other
HD
na
HD_Q92_M_10m_cortex_na_na
HD_Q175_M_10m_striatum_na_na
HD_Q140_M_6m_cortex_na_na
HD_Q92_M_10m_striatum_na_na
HD_Q175_F_2m_striatum_na_na
HD_Q80_M_10m_striatum_na_na
HD_Q175_M_2m_striatum_na_na
HD_Q111_F_6m_striatum_na_na
HD_Q20_M_6m_cortex_na_na
HD_Q80_M_10m_cortex_na_na
HD_Q175_M_10m_cortex_na_na
HD_Q175_F_6m_striatum_na_na
HD_Q140_M_10m_cortex_na_na
HD_Q175_M_6m_striatum_na_na
HD_Q175_F_10m_striatum_na_na
HD_Q111_M_2m_striatum_na_na
HD_Q140_F_10m_striatum_na_na
HD_Q92_F_6m_striatum_na_na
HD_Q175_F_10m_cortex_na_na
HD_Q140_F_6m_striatum_na_na
HD_Q80_M_6m_cortex_na_na
HD_Q111_M_10m_striatum_na_na
HD_Q111_M_6m_cortex_na_na
HD_Q92_F_10m_striatum_na_na
HD_Q92_M_6m_cortex_na_na
HD_Q111_F_10m_striatum_na_na
HD_Q80_F_6m_cortex_na_na
HD_Q111_M_6m_striatum_na_na
HD_Q175_M_6m_cortex_na_na
HD_Q140_M_10m_striatum_na_na
HD_Q140_F_6m_cortex_na_na
HD_Q20_F_6m_cortex_na_na
HD_Q140_M_6m_striatum_na_na
HD_Q175_F_6m_cortex_na_na
HD_Q111_F_6m_cortex_na_na
HD_Q92_F_6m_cortex_na_na
PD
na
PD_MPTP−HCl_na_na_ neurons_na_na
SCA
na
SCA_ATXN130Q−D776_na_12w_cerebellum_na_na
SCA_ATXN182Q_na_12w_cerebellum_na_na
SCA_ATXN182Q_na_28w_cerebellum_na_na
SCA_ATXN130Q−D776_na_5w_cerebellum_na_na
SCA_ATXN130Q−D776_na_28w_cerebellum_na_na
SCA_ATXN130Q_na_5w_cerebellum_na_na
SCA_ATXN182Q_na_5w_cerebellum_na_na
abs(correlation)
0.1
0.2
0.3
0.4
0.5
Correlation
−0.4
−0.2
0.0
0.2
0.4

Figure S11. Correlation Analysis Across Neurodegenerative Mouse Model Transcriptomes

Sets of mouse models representing different neurodegenerative disease are employed in transcriptome correlation analysis with the Kat5 cKO mouse model to assess the similarity of models at the gene (rather than GO term) level. The disease state and linked gene are shown along the left vertical axis in the grey boxes with individual mouse models in rows and identified along the right vertical axis. The transcriptome correlations show greater consistency between the Kat5 cKO and the AD mouse models, denoted by the blue positive correlation values, especially within the APP AD mouse models. The strongest correlation observed is with the p25/cdk5 mouse model, the top two rows within the AD other category. There is strong correlation within both the APP AD models and the Tau AD models across the biological domains. In the CJD mouse models, one correlates strongly while the other is predominantly negatively correlated denoted by the red signal in the second row. The transcriptome correlation with other disease states is more variable, with only two of the five FTD-ALS models showing strong correlation with Kat5 cKO transcriptome. There is little correlation within HD, with almost no correlation within Synapse and anti-correlation within the Tau homeostasis biological domain. Similarly, there is weak correlation within PD and SCA across the models.

Figure S12

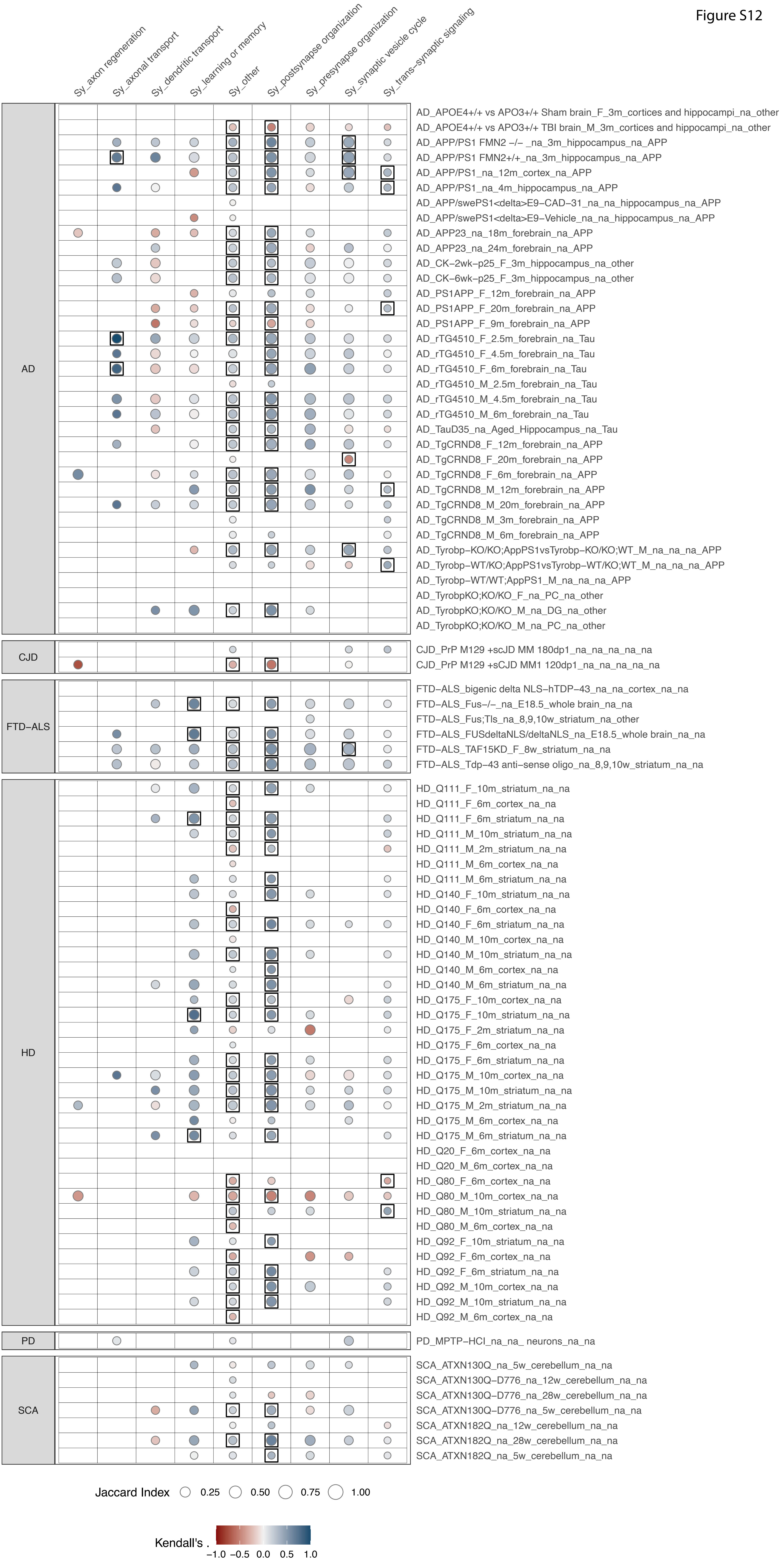

Sy_axon regeneration
Sy_axonal transport
Sy_dendritic transport
Sy_learning or memory
Sy_other
Sy_postsynapse organization
Sy_presynapse organization
Sy_synaptic vesicle cycle
Sy_trans-synaptic signaling
AD
AD_APOE4+/+ vs APO3+/+ Sham brain_F_3m_cortices and hippocampi_na_other
AD_APOE4+/+ vs APO3+/+ TBI brain_M_3m_cortices and hippocampi_na_other
AD_APP/PS1 FMN2 −/− _na_3m_hippocampus_na_APP
AD_APP/PS1 FMN2+/+_na_3m_hippocampus_na_APP
AD_APP/PS1_na_12m_cortex_na_APP
AD_APP/PS1_na_4m_hippocampus_na_APP
AD_APP/swePS1<delta>E9−CAD−31_na_na_hippocampus_na_APP
AD_APP/swePS1<delta>E9−Vehicle_na_na_hippocampus_na_APP
AD_APP23_na_18m_forebrain_na_APP
AD_APP23_na_24m_forebrain_na_APP
AD_CK−2wk−p25_F_3m_hippocampus_na_other
AD_CK−6wk−p25_F_3m_hippocampus_na_other
AD_PS1APP_F_12m_forebrain_na_APP
AD_PS1APP_F_20m_forebrain_na_APP
AD_PS1APP_F_9m_forebrain_na_APP
AD_rTG4510_F_2.5m_forebrain_na_Tau
AD_rTG4510_F_4.5m_forebrain_na_Tau
AD_rTG4510_F_6m_forebrain_na_Tau
AD_rTG4510_M_2.5m_forebrain_na_Tau
AD_rTG4510_M_4.5m_forebrain_na_Tau
AD_rTG4510_M_6m_forebrain_na_Tau
AD_TauD35_na_Aged_Hippocampus_na_Tau
AD_TgCRND8_F_12m_forebrain_na_APP
AD_TgCRND8_F_20m_forebrain_na_APP
AD_TgCRND8_F_6m_forebrain_na_APP
AD_TgCRND8_M_12m_forebrain_na_APP
AD_TgCRND8_M_20m_forebrain_na_APP
AD_TgCRND8_M_3m_forebrain_na_APP
AD_TgCRND8_M_6m_forebrain_na_APP
AD_Tyrobp−KO/KO;AppPS1vsTyrobp−KO/KO;WT_M_na_na_na_APP
AD_Tyrobp−WT/KO;AppPS1vsTyrobp−WT/KO;WT_M_na_na_na_APP
AD_Tyrobp−WT/WT;AppPS1_M_na_na_na_APP
AD_TyrobpKO;KO/KO_F_na_PC_na_other
AD_TyrobpKO;KO/KO_M_na_DG_na_other
AD_TyrobpKO;KO/KO_M_na_PC_na_other
CJD
CJD_PrP M129 +scJD MM 180dp1_na_na_na_na_na
CJD_PrP M129 +sCJD MM1 120dp1_na_na_na_na_na
FTD−ALS
FTD−ALS_bigenic delta NLS−hTDP−43_na_na_cortex_na_na
FTD−ALS_Fus−/−_na_E18.5_whole brain_na_na
FTD−ALS_Fus;Tls_na_8,9,10w_striatum_na_other
FTD−ALS_FUSdeltaNLS/deltaNLS_na_E18.5_whole brain_na_na
FTD−ALS_TAF15KD_F_8w_striatum_na_na
FTD−ALS_Tdp−43 anti−sense oligo_na_8,9,10w_striatum_na_na
HD
HD_Q111_F_10m_striatum_na_na
HD_Q111_F_6m_cortex_na_na
HD_Q111_F_6m_striatum_na_na
HD_Q111_M_10m_striatum_na_na
HD_Q111_M_2m_striatum_na_na
HD_Q111_M_6m_cortex_na_na
HD_Q111_M_6m_striatum_na_na
HD_Q140_F_10m_striatum_na_na
HD_Q140_F_6m_cortex_na_na
HD_Q140_F_6m_striatum_na_na
HD_Q140_M_10m_cortex_na_na
HD_Q140_M_10m_striatum_na_na
HD_Q140_M_6m_cortex_na_na
HD_Q140_M_6m_striatum_na_na
HD_Q175_F_10m_cortex_na_na
HD_Q175_F_10m_striatum_na_na
HD_Q175_F_2m_striatum_na_na
HD_Q175_F_6m_cortex_na_na
HD_Q175_F_6m_striatum_na_na
HD_Q175_M_10m_cortex_na_na
HD_Q175_M_10m_striatum_na_na
HD_Q175_M_2m_striatum_na_na
HD_Q175_M_6m_cortex_na_na
HD_Q175_M_6m_striatum_na_na
HD_Q20_F_6m_cortex_na_na
HD_Q20_M_6m_cortex_na_na
HD_Q80_F_6m_cortex_na_na
HD_Q80_M_10m_cortex_na_na
HD_Q80_M_10m_striatum_na_na
HD_Q80_M_6m_cortex_na_na
HD_Q92_F_10m_striatum_na_na
HD_Q92_F_6m_cortex_na_na
HD_Q92_F_6m_striatum_na_na
HD_Q92_M_10m_cortex_na_na
HD_Q92_M_10m_striatum_na_na
HD_Q92_M_6m_cortex_na_na
PD
PD_MPTP−HCl_na_na_ neurons_na_na
SCA
SCA_ATXN130Q_na_5w_cerebellum_na_na
SCA_ATXN130Q−D776_na_12w_cerebellum_na_na
SCA_ATXN130Q−D776_na_28w_cerebellum_na_na
SCA_ATXN130Q−D776_na_5w_cerebellum_na_na
SCA_ATXN182Q_na_12w_cerebellum_na_na
SCA_ATXN182Q_na_28w_cerebellum_na_na
SCA_ATXN182Q_na_5w_cerebellum_na_na
Jaccard Index 0.25 0.50 0.75 1.00
Kendall's .
−1.0 −0.5 0.0 0.5 1.0

Figure S12. Comparison of Neurodegenerative Mouse Models Across Synapse Subdomains.

The AD biological subdomains describe areas of disease association process at greater resolution than the parent domains. Specifically examining synaptic processes across the subdomains is motivated by the highest consistency between diseases observed within the Synapse biodomain. Within the AD mouse models, there is high correlation with the Kat5 cKO GO term enrichment within 'other' (terms that do not map to a specific subdomain), post-synaptic organization, presynaptic organization, and synaptic vesicle cycle. These correlation of enriched terms across models is absent within CJD but is shared with the majority of FTD-ALS models. The correlation of enrichment in 'other' and post-synaptic organization is shared within HD and SCA, but not within the one PD model. The statistical significance is noted by the solid boxes around the enriched terms within each model.

Figure S13

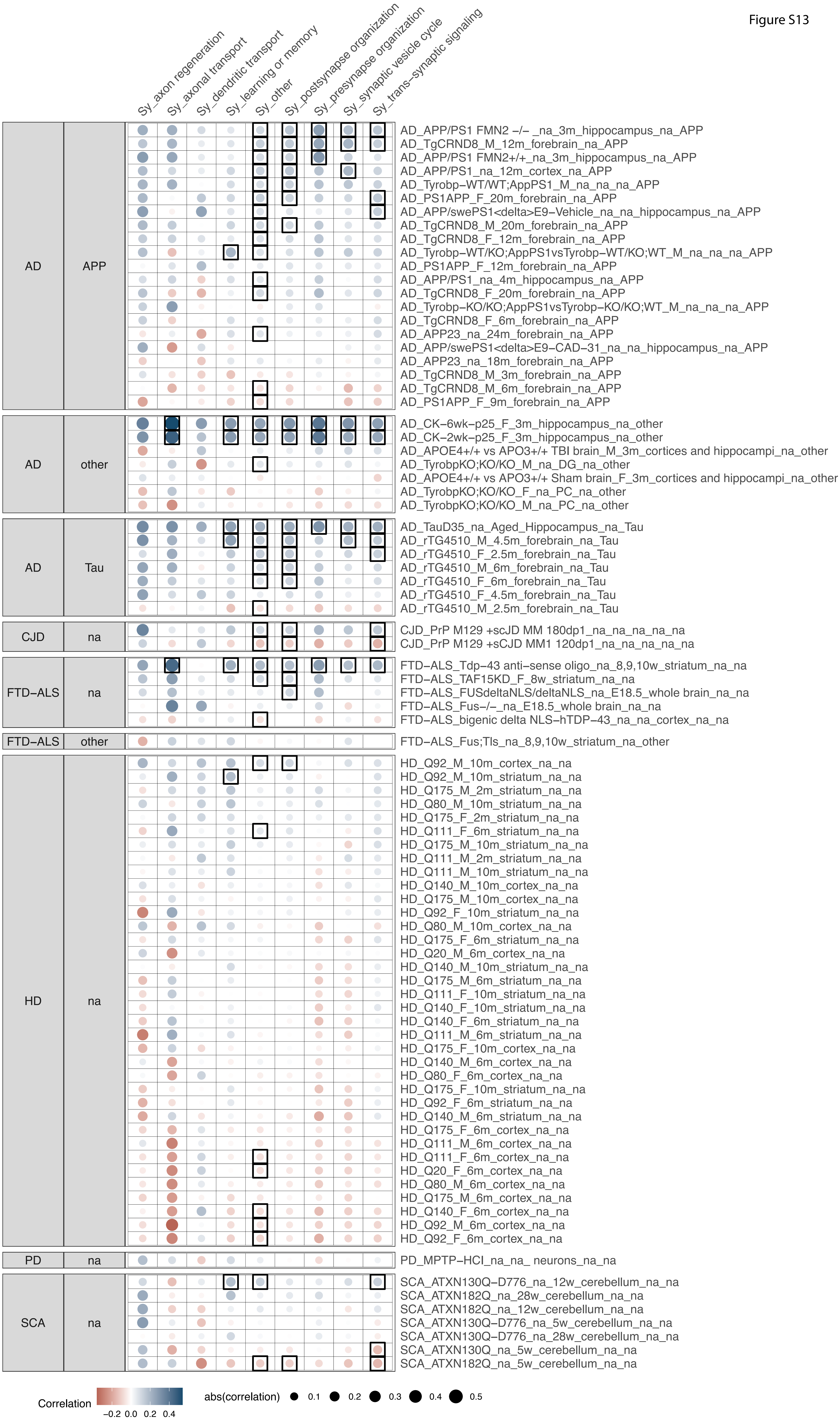
Sy_axon regeneration
Sy_axonal transport
Sy_dendritic transport
Sy_learning or memory
Sy_other
Sy_postsynapse organization
Sy_presynapse organization
Sy_synaptic vesicle cycle
Sy_trans−synaptic signaling
AD
APP
AD_APP/PS1 FMN2 −/− _na_3m_hippocampus_na_APP
AD_TgCRND8_M_12m_forebrain_na_APP
AD_APP/PS1 FMN2+/+_na_3m_hippocampus_na_APP
AD_APP/PS1_na_12m_cortex_na_APP
AD_Tyrobp−WT/WT;AppPS1_M_na_na_na_APP
AD_PS1APP_F_20m_forebrain_na_APP
AD_APP/swePS1<delta>E9−Vehicle_na_na_hippocampus_na_APP
AD_TgCRND8_M_20m_forebrain_na_APP
AD_TgCRND8_F_12m_forebrain_na_APP
AD_Tyrobp−WT/KO;AppPS1vsTyrobp−WT/KO;WT_M_na_na_na_APP
AD_PS1APP_F_12m_forebrain_na_APP
AD_APP/PS1_na_4m_hippocampus_na_APP
AD_TgCRND8_F_20m_forebrain_na_APP
AD_Tyrobp−KO/KO;AppPS1vsTyrobp−KO/KO;WT_M_na_na_na_APP
AD_TgCRND8_F_6m_forebrain_na_APP
AD_APP23_na_24m_forebrain_na_APP
AD_APP/swePS1<delta>E9−CAD−31_na_na_hippocampus_na_APP
AD_APP23_na_18m_forebrain_na_APP
AD_TgCRND8_M_3m_forebrain_na_APP
AD_TgCRND8_M_6m_forebrain_na_APP
AD_PS1APP_F_9m_forebrain_na_APP
AD
other
AD_CK−6wk−p25_F_3m_hippocampus_na_other
AD_CK−2wk−p25_F_3m_hippocampus_na_other
AD_APOE4+/+ vs APO3+/+ TBI brain_M_3m_cortices and hippocampi_na_other
AD_TyrobpKO;KO/KO_M_na_DG_na_other
AD_APOE4+/+ vs APO3+/+ Sham brain_F_3m_cortices and hippocampi_na_other
AD_TyrobpKO;KO/KO_F_na_PC_na_other
AD_TyrobpKO;KO/KO_M_na_PC_na_other
AD
Tau
AD_TauD35_na_Aged_Hippocampus_na_Tau
AD_rTG4510_M_4.5m_forebrain_na_Tau
AD_rTG4510_F_2.5m_forebrain_na_Tau
AD_rTG4510_M_6m_forebrain_na_Tau
AD_rTG4510_F_6m_forebrain_na_Tau
AD_rTG4510_F_4.5m_forebrain_na_Tau
AD_rTG4510_M_2.5m_forebrain_na_Tau
CJD
na
CJD_PrP M129 +scJD MM 180dp1_na_na_na_na_na
CJD_PrP M129 +sCJD MM1 120dp1_na_na_na_na_na
FTD−ALS
na
FTD−ALS_Tdp−43 anti−sense oligo_na_8,9,10w_striatum_na_na
FTD−ALS_TAF15KD_F_8w_striatum_na_na
FTD−ALS_FUSdeltaNLS/deltaNLS_na_E18.5_whole brain_na_na
FTD−ALS_Fus−/−_na_E18.5_whole brain_na_na
FTD−ALS_bigenic delta NLS−hTDP−43_na_na_cortex_na_na
FTD−ALS
other
FTD−ALS_Fus;Tls_na_8,9,10w_striatum_na_other
HD
na
HD_Q92_M_10m_cortex_na_na
HD_Q92_M_10m_striatum_na_na
HD_Q175_M_2m_striatum_na_na
HD_Q80_M_10m_striatum_na_na
HD_Q175_F_2m_striatum_na_na
HD_Q111_F_6m_striatum_na_na
HD_Q175_M_10m_striatum_na_na
HD_Q111_M_2m_striatum_na_na
HD_Q111_M_10m_striatum_na_na
HD_Q140_M_10m_cortex_na_na
HD_Q175_M_10m_cortex_na_na
HD_Q92_F_10m_striatum_na_na
HD_Q80_M_10m_cortex_na_na
HD_Q175_F_6m_striatum_na_na
HD_Q20_M_6m_cortex_na_na
HD_Q140_M_10m_striatum_na_na
HD_Q175_M_6m_striatum_na_na
HD_Q111_F_10m_striatum_na_na
HD_Q140_F_10m_striatum_na_na
HD_Q140_F_6m_striatum_na_na
HD_Q111_M_6m_striatum_na_na
HD_Q175_F_10m_cortex_na_na
HD_Q140_M_6m_cortex_na_na
HD_Q80_F_6m_cortex_na_na
HD_Q175_F_10m_striatum_na_na
HD_Q92_F_6m_striatum_na_na
HD_Q140_M_6m_striatum_na_na
HD_Q175_F_6m_cortex_na_na
HD_Q111_M_6m_cortex_na_na
HD_Q111_F_6m_cortex_na_na
HD_Q20_F_6m_cortex_na_na
HD_Q80_M_6m_cortex_na_na
HD_Q175_M_6m_cortex_na_na
HD_Q140_F_6m_cortex_na_na
HD_Q92_M_6m_cortex_na_na
HD_Q92_F_6m_cortex_na_na
PD
na
PD_MPTP−HCl_na_na_ neurons_na_na
SCA
na
SCA_ATXN130Q−D776_na_12w_cerebellum_na_na
SCA_ATXN182Q_na_28w_cerebellum_na_na
SCA_ATXN182Q_na_12w_cerebellum_na_na
SCA_ATXN130Q−D776_na_5w_cerebellum_na_na
SCA_ATXN130Q−D776_na_28w_cerebellum_na_na
SCA_ATXN130Q_na_5w_cerebellum_na_na
SCA_ATXN182Q_na_5w_cerebellum_na_na
Correlation
−0.2 0.0 0.2 0.4
abs(correlation)
0.1
0.2
0.3
0.4
0.5

Figure S13. Transcriptomic Correlation Analysis Across Synaptic Subdomains Between Neurodegenerative Mouse Models and Kat5 cKO.

The set of neurodegenerative mouse models are identified along the right hand vertical portion of the figure while the disease state and associated gene are boxed at the left side of the figure. There is broader correlation within the AD mouse models with Kat5 cKO denoted by the blue signal within the AD states. The correlation holds within APP, p25/cdk5 (other), and Tau based AD mouse models. The transcriptomic correlation is weaker between the other neurodegenerative mouse models with one CJD demonstrating positive correlation while the other shows negative correlation with Kat5 cKO across the synaptic subdomains. FTD-ALS TDP43 model shows strong correlation with Kat5 cKO across synaptic subdomains, while the other FTD-ALS models possess much weaker associations. There is a weak, and often negative correlation (denoted by red dots), within the HD, PD and SCA models.

Figure S14

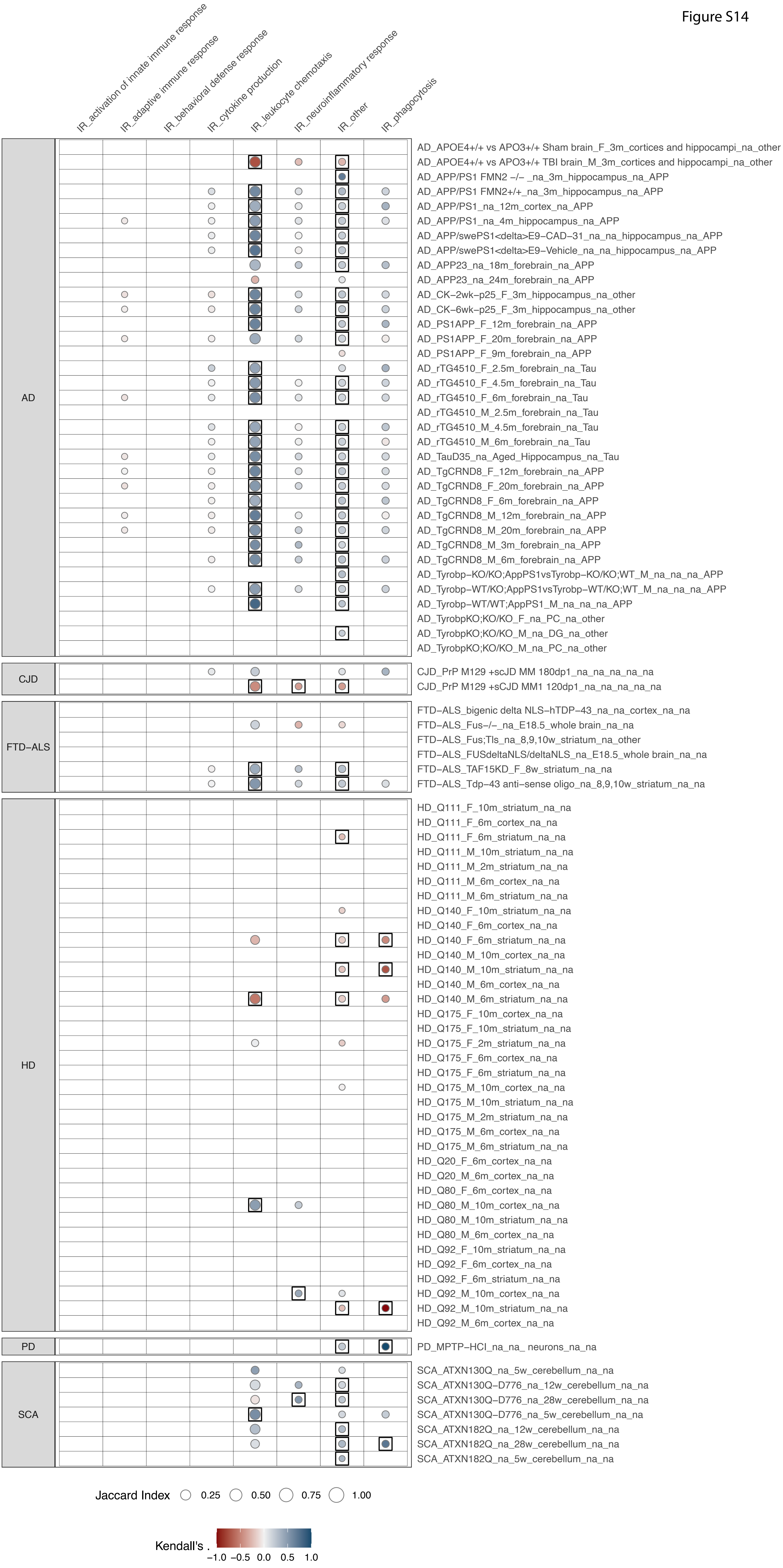

IR_activation of innate immune response
IR_adaptive immune response
IR_behavioral defense response
IR_cytokine production
IR_leukocyte chemotaxis
IR_neuroinflammatory response
IR_other
IR_phagocytosis
AD
CJD
FTD−ALS
HD
PD
SCA
AD_APOE4+/+ vs APO3+/+ Sham brain_F_3m_cortices and hippocampi_na_other
AD_APOE4+/+ vs APO3+/+ TBI brain_M_3m_cortices and hippocampi_na_other
AD_APP/PS1 FMN2 −/− _na_3m_hippocampus_na_APP
AD_APP/PS1 FMN2+/+_na_3m_hippocampus_na_APP
AD_APP/PS1_na_12m_cortex_na_APP
AD_APP/PS1_na_4m_hippocampus_na_APP
AD_APP/swePS1<delta>E9−CAD−31_na_na_hippocampus_na_APP
AD_APP/swePS1<delta>E9−Vehicle_na_na_hippocampus_na_APP
AD_APP23_na_18m_forebrain_na_APP
AD_APP23_na_24m_forebrain_na_APP
AD_CK−2wk−p25_F_3m_hippocampus_na_other
AD_CK−6wk−p25_F_3m_hippocampus_na_other
AD_PS1APP_F_12m_forebrain_na_APP
AD_PS1APP_F_20m_forebrain_na_APP
AD_PS1APP_F_9m_forebrain_na_APP
AD_rTG4510_F_2.5m_forebrain_na_Tau
AD_rTG4510_F_4.5m_forebrain_na_Tau
AD_rTG4510_F_6m_forebrain_na_Tau
AD_rTG4510_M_2.5m_forebrain_na_Tau
AD_rTG4510_M_4.5m_forebrain_na_Tau
AD_rTG4510_M_6m_forebrain_na_Tau
AD_TauD35_na_Aged_Hippocampus_na_Tau
AD_TgCRND8_F_12m_forebrain_na_APP
AD_TgCRND8_F_20m_forebrain_na_APP
AD_TgCRND8_F_6m_forebrain_na_APP
AD_TgCRND8_M_12m_forebrain_na_APP
AD_TgCRND8_M_20m_forebrain_na_APP
AD_TgCRND8_M_3m_forebrain_na_APP
AD_TgCRND8_M_6m_forebrain_na_APP
AD_Tyrobp−KO/KO;AppPS1vsTyrobp−KO/KO;WT_M_na_na_na_APP
AD_Tyrobp−WT/KO;AppPS1vsTyrobp−WT/KO;WT_M_na_na_na_APP
AD_Tyrobp−WT/WT;AppPS1_M_na_na_na_APP
AD_TyrobpKO;KO/KO_F_na_PC_na_other
AD_TyrobpKO;KO/KO_M_na_DG_na_other
AD_TyrobpKO;KO/KO_M_na_PC_na_other
CJD_PrP M129 +scJD MM 180dp1_na_na_na_na_na
CJD_PrP M129 +sCJD MM1 120dp1_na_na_na_na_na
FTD−ALS_bigenic delta NLS−hTDP−43_na_na_cortex_na_na
FTD−ALS_Fus−/−_na_E18.5_whole brain_na_na
FTD−ALS_Fus;Tls_na_8,9,10w_striatum_na_other
FTD−ALS_FUSdeltaNLS/deltaNLS_na_E18.5_whole brain_na_na
FTD−ALS_TAF15KD_F_8w_striatum_na_na
FTD−ALS_Tdp−43 anti−sense oligo_na_8,9,10w_striatum_na_na
HD_Q111_F_10m_striatum_na_na
HD_Q111_F_6m_cortex_na_na
HD_Q111_F_6m_striatum_na_na
HD_Q111_M_10m_striatum_na_na
HD_Q111_M_2m_striatum_na_na
HD_Q111_M_6m_cortex_na_na
HD_Q111_M_6m_striatum_na_na
HD_Q140_F_10m_striatum_na_na
HD_Q140_F_6m_cortex_na_na
HD_Q140_F_6m_striatum_na_na
HD_Q140_M_10m_cortex_na_na
HD_Q140_M_10m_striatum_na_na
HD_Q140_M_6m_cortex_na_na
HD_Q140_M_6m_striatum_na_na
HD_Q175_F_10m_cortex_na_na
HD_Q175_F_10m_striatum_na_na
HD_Q175_F_2m_striatum_na_na
HD_Q175_F_6m_cortex_na_na
HD_Q175_F_6m_striatum_na_na
HD_Q175_M_10m_cortex_na_na
HD_Q175_M_10m_striatum_na_na
HD_Q175_M_2m_striatum_na_na
HD_Q175_M_6m_cortex_na_na
HD_Q175_M_6m_striatum_na_na
HD_Q20_F_6m_cortex_na_na
HD_Q20_M_6m_cortex_na_na
HD_Q80_F_6m_cortex_na_na
HD_Q80_M_10m_cortex_na_na
HD_Q80_M_10m_striatum_na_na
HD_Q80_M_6m_cortex_na_na
HD_Q92_F_10m_striatum_na_na
HD_Q92_F_6m_cortex_na_na
HD_Q92_F_6m_striatum_na_na
HD_Q92_M_10m_cortex_na_na
HD_Q92_M_10m_striatum_na_na
HD_Q92_M_6m_cortex_na_na
PD_MPTP−HCl_na_na_ neurons_na_na
SCA_ATXN130Q_na_5w_cerebellum_na_na
SCA_ATXN130Q−D776_na_12w_cerebellum_na_na
SCA_ATXN130Q−D776_na_28w_cerebellum_na_na
SCA_ATXN130Q−D776_na_5w_cerebellum_na_na
SCA_ATXN182Q_na_12w_cerebellum_na_na
SCA_ATXN182Q_na_28w_cerebellum_na_na
SCA_ATXN182Q_na_5w_cerebellum_na_na
Jaccard Index
0.25
0.50
0.75
1.00
Kendall's .
−1.0 −0.5 0.0 0.5 1.0

Figure S14. Comparison of Immune Response Subdomain GSEA Enrichment Across Neurodegenerative Mouse Models.

The increase in immune response (IR) genes is consistently observed in our analysis of AD human subtypes and mouse models. We explored the correlation of GSEA enrichment of terms within the immune response biodomain across the subdomains within the set of neurodegenerative mouse models. The disease states are shown at the left and the specific models are shown at the right. The blue dots represent positive correlation while red dots represent a negative correlation with the Kat5 cKO mouse model. There is strong correlation within almost all the AD models within the IR leukocyte chemotaxis subdomain, with the notable exception of the APOE4+/+ TBI model, which shows strong anti-correlation (denoted by the boxed red dot at the top of the column). There is minimal shared signal within the other neurodegenerative disease models with the exception of bottom two FTD-ALS models. There is minimal correlation within HD, with stronger negative correlation across models than positive correlation. PD and SCA show some correlation within 'other' and phagocytosis, and SCA also shows positive correlation within leukocyte chemotaxis and neuroinflammatory response in some models.

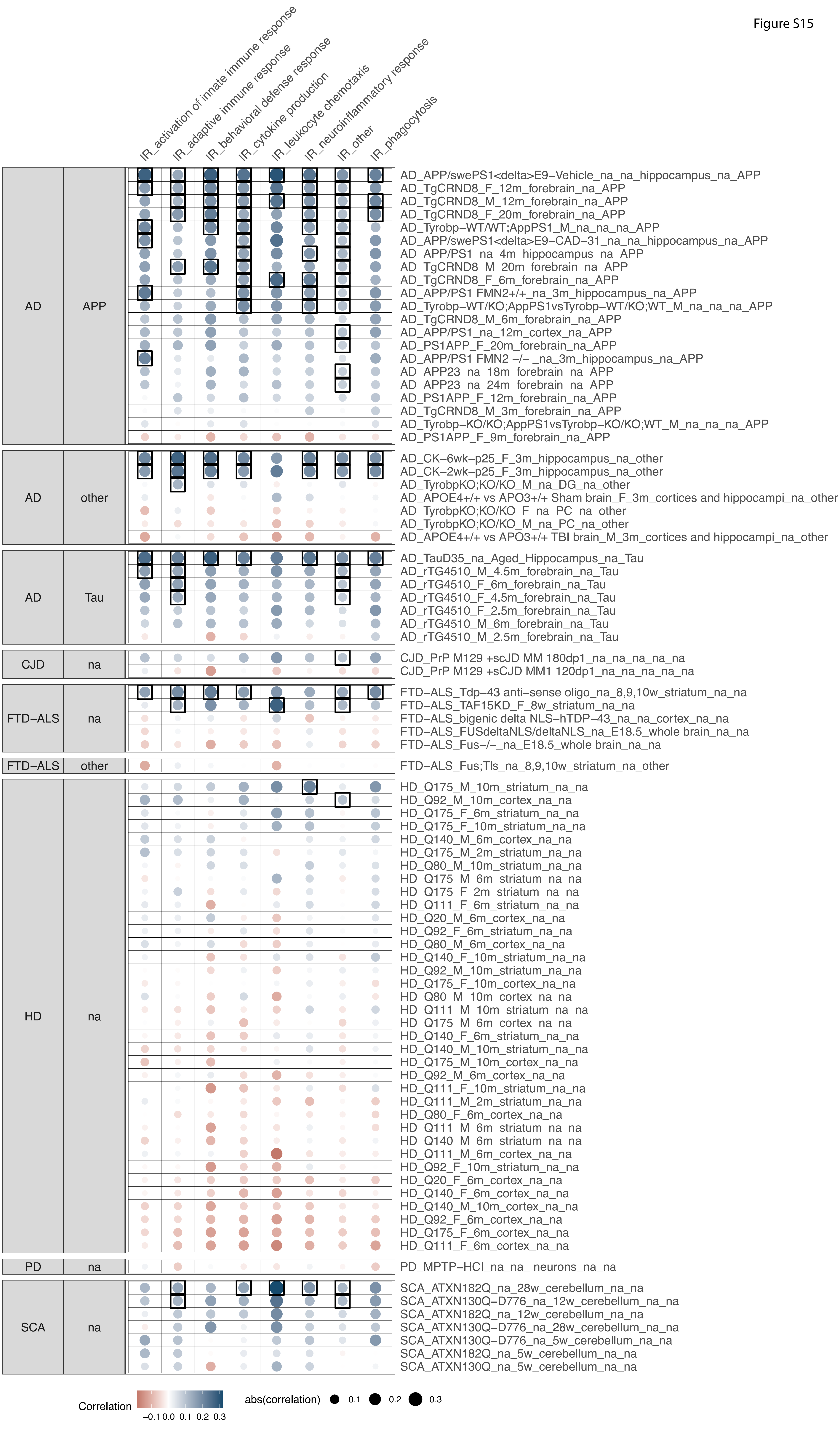
Figure S15
IR_activation of innate immune response
IR_adaptive immune response
IR_behavioral defense response
IR_cytokine production
IR_leukocyte chemotaxis
IR_neuroinflammatory response
IR_other
IR_phagocytosis
AD
APP
AD_APP/swePS1<delta>E9−Vehicle_na_na_hippocampus_na_APP
AD_TgCRND8_F_12m_forebrain_na_APP
AD_TgCRND8_M_12m_forebrain_na_APP
AD_TgCRND8_F_20m_forebrain_na_APP
AD_Tyrobp−WT/WT;AppPS1_M_na_na_na_APP
AD_APP/swePS1<delta>E9−CAD−31_na_na_hippocampus_na_APP
AD_APP/PS1_na_4m_hippocampus_na_APP
AD_TgCRND8_M_20m_forebrain_na_APP
AD_TgCRND8_F_6m_forebrain_na_APP
AD_APP/PS1 FMN2+/+_na_3m_hippocampus_na_APP
AD_Tyrobp−WT/KO;AppPS1vsTyrobp−WT/KO;WT_M_na_na_na_APP
AD_TgCRND8_M_6m_forebrain_na_APP
AD_APP/PS1_na_12m_cortex_na_APP
AD_PS1APP_F_20m_forebrain_na_APP
AD_APP/PS1 FMN2 −/− _na_3m_hippocampus_na_APP
AD_APP23_na_18m_forebrain_na_APP
AD_APP23_na_24m_forebrain_na_APP
AD_PS1APP_F_12m_forebrain_na_APP
AD_TgCRND8_M_3m_forebrain_na_APP
AD_Tyrobp−KO/KO;AppPS1vsTyrobp−KO/KO;WT_M_na_na_na_APP
AD_PS1APP_F_9m_forebrain_na_APP
AD
other
AD_CK−6wk−p25_F_3m_hippocampus_na_other
AD_CK−2wk−p25_F_3m_hippocampus_na_other
AD_TyrobpKO;KO/KO_M_na_DG_na_other
AD_APOE4+/+ vs APO3+/+ Sham brain_F_3m_cortices and hippocampi_na_other
AD_TyrobpKO;KO/KO_F_na_PC_na_other
AD_TyrobpKO;KO/KO_M_na_PC_na_other
AD_APOE4+/+ vs APO3+/+ TBI brain_M_3m_cortices and hippocampi_na_other
AD
Tau
AD_TauD35_na_Aged_Hippocampus_na_Tau
AD_rTG4510_M_4.5m_forebrain_na_Tau
AD_rTG4510_F_6m_forebrain_na_Tau
AD_rTG4510_F_4.5m_forebrain_na_Tau
AD_rTG4510_F_2.5m_forebrain_na_Tau
AD_rTG4510_M_6m_forebrain_na_Tau
AD_rTG4510_M_2.5m_forebrain_na_Tau
CJD
na
CJD_PrP M129 +scJD MM 180dp1_na_na_na_na_na
CJD_PrP M129 +sCJD MM1 120dp1_na_na_na_na_na
FTD−ALS
na
FTD−ALS_Tdp−43 anti−sense oligo_na_8,9,10w_striatum_na_na
FTD−ALS_TAF15KD_F_8w_striatum_na_na
FTD−ALS_bigenic delta NLS−hTDP−43_na_na_cortex_na_na
FTD−ALS_FUSdeltaNLS/deltaNLS_na_E18.5_whole brain_na_na
FTD−ALS_Fus−/−_na_E18.5_whole brain_na_na
FTD−ALS
other
FTD−ALS_Fus;Tls_na_8,9,10w_striatum_na_other
HD
na
HD_Q175_M_10m_striatum_na_na
HD_Q92_M_10m_cortex_na_na
HD_Q175_F_6m_striatum_na_na
HD_Q175_F_10m_striatum_na_na
HD_Q140_M_6m_cortex_na_na
HD_Q175_M_2m_striatum_na_na
HD_Q80_M_10m_striatum_na_na
HD_Q175_M_6m_striatum_na_na
HD_Q175_F_2m_striatum_na_na
HD_Q111_F_6m_striatum_na_na
HD_Q20_M_6m_cortex_na_na
HD_Q92_F_6m_striatum_na_na
HD_Q80_M_6m_cortex_na_na
HD_Q140_F_10m_striatum_na_na
HD_Q92_M_10m_striatum_na_na
HD_Q175_F_10m_cortex_na_na
HD_Q80_M_10m_cortex_na_na
HD_Q111_M_10m_striatum_na_na
HD_Q175_M_6m_cortex_na_na
HD_Q140_F_6m_striatum_na_na
HD_Q140_M_10m_striatum_na_na
HD_Q175_M_10m_cortex_na_na
HD_Q92_M_6m_cortex_na_na
HD_Q111_F_10m_striatum_na_na
HD_Q111_M_2m_striatum_na_na
HD_Q80_F_6m_cortex_na_na
HD_Q111_M_6m_striatum_na_na
HD_Q140_M_6m_striatum_na_na
HD_Q111_M_6m_cortex_na_na
HD_Q92_F_10m_striatum_na_na
HD_Q20_F_6m_cortex_na_na
HD_Q140_F_6m_cortex_na_na
HD_Q140_M_10m_cortex_na_na
HD_Q92_F_6m_cortex_na_na
HD_Q175_F_6m_cortex_na_na
HD_Q111_F_6m_cortex_na_na
PD
na
PD_MPTP−HCl_na_na_ neurons_na_na
SCA
na
SCA_ATXN182Q_na_28w_cerebellum_na_na
SCA_ATXN130Q−D776_na_12w_cerebellum_na_na
SCA_ATXN182Q_na_12w_cerebellum_na_na
SCA_ATXN130Q−D776_na_28w_cerebellum_na_na
SCA_ATXN130Q−D776_na_5w_cerebellum_na_na
SCA_ATXN182Q_na_5w_cerebellum_na_na
SCA_ATXN130Q_na_5w_cerebellum_na_na
Correlation
−0.1 0.0 0.1 0.2 0.3
abs(correlation)
0.1
0.2
0.3

Figure S15. Transcriptomic Comparison of Neurodegenerative Mouse Models within the Immune Response Subdomains.

The neurodegenerative mouse models representing AD, FTD, ALS, CJD, HD, PD and SCA are compared for transcriptomic correlation with the Kat5 cKO mouse model within the subdomains from the Immune Response (IR) biodomain. The correlation between all the APP and Tau mouse models is extremely high, denoted by the boxed deep blue dots. The mouse models are arrayed along the righthand side and the disease state and associated gene are shown on the left. Outside of APP and Tau models, there is strong correlation within the p25/cdk5 mouse model within the 'other' group in the AD models. However, there is minimal or negative correlation within the TyroBP or APOE4 model mice. The FTD-ALS mice show strong correlation within the first two models but demonstrates negative correlation within the last two models. There is little correlation within any of the HD mouse models, with many showing negative correlation with the Kat5 cKO transcriptomic profile. The SCA models do show strong positive correlation with the Kat5 cKO in most subdomains.

Figure S16

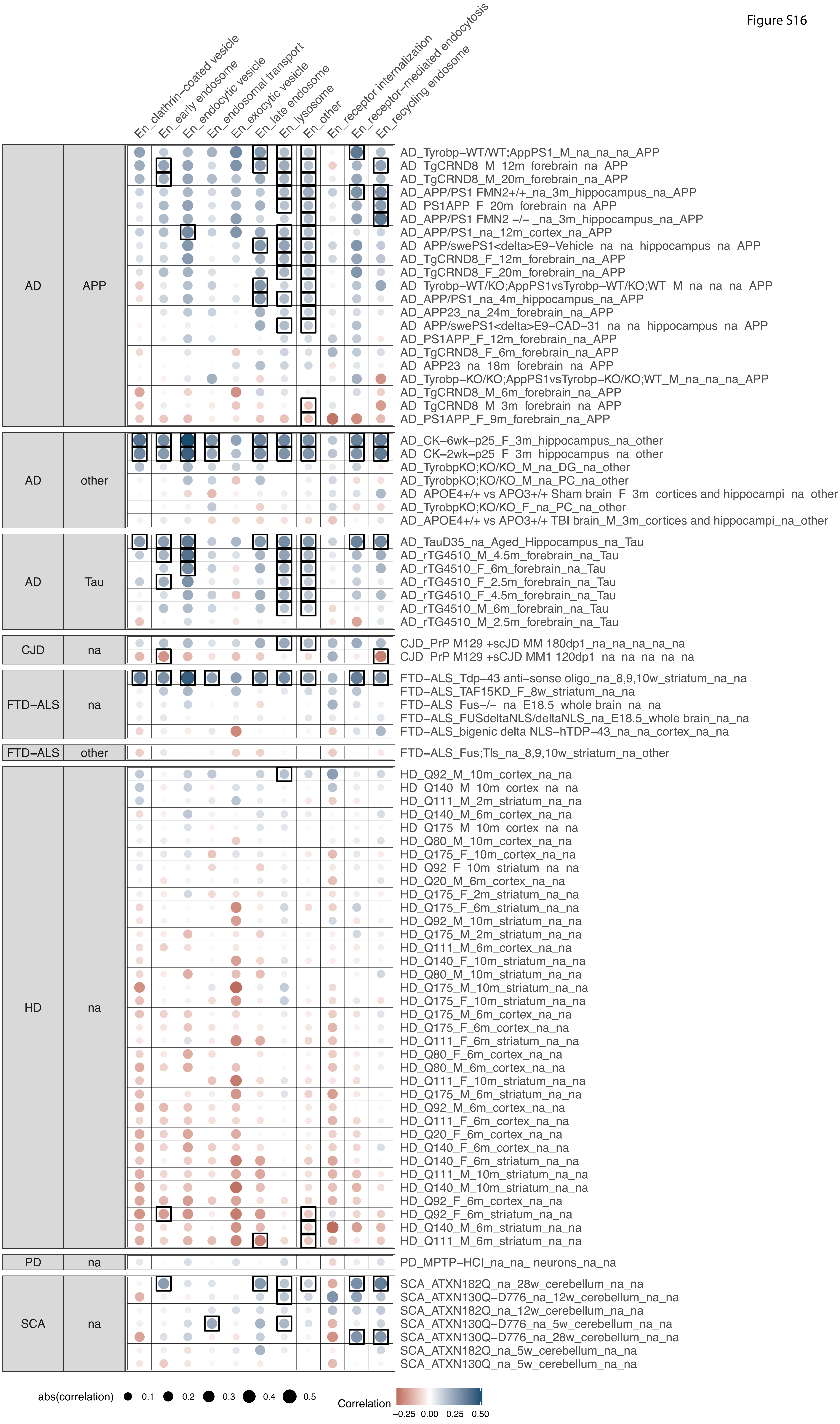
En_clathrin−coated vesicle
En_early endosome
En_endocytic vesicle
En_endosomal transport
En_exocytic vesicle
En_late endosome
En_lysosome
En_other
En_receptor internalization
En_receptor−mediated endocytosis
En_recycling endosome
AD
APP
AD_Tyrobp−WT/WT;AppPS1_M_na_na_na_APP
AD_TgCRND8_M_12m_forebrain_na_APP
AD_TgCRND8_M_20m_forebrain_na_APP
AD_APP/PS1 FMN2+/+_na_3m_hippocampus_na_APP
AD_PS1APP_F_20m_forebrain_na_APP
AD_APP/PS1 FMN2 −/− _na_3m_hippocampus_na_APP
AD_APP/PS1_na_12m_cortex_na_APP
AD_APP/swePS1<delta>E9−Vehicle_na_na_hippocampus_na_APP
AD_TgCRND8_F_12m_forebrain_na_APP
AD_TgCRND8_F_20m_forebrain_na_APP
AD_Tyrobp−WT/KO;AppPS1vsTyrobp−WT/KO;WT_M_na_na_na_APP
AD_APP/PS1_na_4m_hippocampus_na_APP
AD_APP23_na_24m_forebrain_na_APP
AD_APP/swePS1<delta>E9−CAD−31_na_na_hippocampus_na_APP
AD_PS1APP_F_12m_forebrain_na_APP
AD_TgCRND8_F_6m_forebrain_na_APP
AD_APP23_na_18m_forebrain_na_APP
AD_Tyrobp−KO/KO;AppPS1vsTyrobp−KO/KO;WT_M_na_na_na_APP
AD_TgCRND8_M_6m_forebrain_na_APP
AD_TgCRND8_M_3m_forebrain_na_APP
AD_PS1APP_F_9m_forebrain_na_APP
AD
other
AD_CK−6wk−p25_F_3m_hippocampus_na_other
AD_CK−2wk−p25_F_3m_hippocampus_na_other
AD_TyrobpKO;KO/KO_M_na_DG_na_other
AD_TyrobpKO;KO/KO_M_na_PC_na_other
AD_APOE4+/+ vs APO3+/+ Sham brain_F_3m_cortices and hippocampi_na_other
AD_TyrobpKO;KO/KO_F_na_PC_na_other
AD_APOE4+/+ vs APO3+/+ TBI brain_M_3m_cortices and hippocampi_na_other
AD
Tau
AD_TauD35_na_Aged_Hippocampus_na_Tau
AD_rTG4510_M_4.5m_forebrain_na_Tau
AD_rTG4510_F_6m_forebrain_na_Tau
AD_rTG4510_F_2.5m_forebrain_na_Tau
AD_rTG4510_F_4.5m_forebrain_na_Tau
AD_rTG4510_M_6m_forebrain_na_Tau
AD_rTG4510_M_2.5m_forebrain_na_Tau
CJD
na
CJD_PrP M129 +scJD MM 180dp1_na_na_na_na_na
CJD_PrP M129 +sCJD MM1 120dp1_na_na_na_na_na
FTD−ALS
na
FTD−ALS_Tdp−43 anti−sense oligo_na_8,9,10w_striatum_na_na
FTD−ALS_TAF15KD_F_8w_striatum_na_na
FTD−ALS_Fus−/−_na_E18.5_whole brain_na_na
FTD−ALS_FUSdeltaNLS/deltaNLS_na_E18.5_whole brain_na_na
FTD−ALS_bigenic delta NLS−hTDP−43_na_na_cortex_na_na
FTD−ALS
other
FTD−ALS_Fus;Tls_na_8,9,10w_striatum_na_other
HD
na
HD_Q92_M_10m_cortex_na_na
HD_Q140_M_10m_cortex_na_na
HD_Q111_M_2m_striatum_na_na
HD_Q140_M_6m_cortex_na_na
HD_Q175_M_10m_cortex_na_na
HD_Q80_M_10m_cortex_na_na
HD_Q175_F_10m_cortex_na_na
HD_Q92_F_10m_striatum_na_na
HD_Q20_M_6m_cortex_na_na
HD_Q175_F_2m_striatum_na_na
HD_Q175_F_6m_striatum_na_na
HD_Q92_M_10m_striatum_na_na
HD_Q175_M_2m_striatum_na_na
HD_Q111_M_6m_cortex_na_na
HD_Q140_F_10m_striatum_na_na
HD_Q80_M_10m_striatum_na_na
HD_Q175_M_10m_striatum_na_na
HD_Q175_F_10m_striatum_na_na
HD_Q175_M_6m_cortex_na_na
HD_Q175_F_6m_cortex_na_na
HD_Q111_F_6m_striatum_na_na
HD_Q80_F_6m_cortex_na_na
HD_Q80_M_6m_cortex_na_na
HD_Q111_F_10m_striatum_na_na
HD_Q175_M_6m_striatum_na_na
HD_Q92_M_6m_cortex_na_na
HD_Q111_F_6m_cortex_na_na
HD_Q20_F_6m_cortex_na_na
HD_Q140_F_6m_cortex_na_na
HD_Q140_F_6m_striatum_na_na
HD_Q111_M_10m_striatum_na_na
HD_Q140_M_10m_striatum_na_na
HD_Q92_F_6m_cortex_na_na
HD_Q92_F_6m_striatum_na_na
HD_Q140_M_6m_striatum_na_na
HD_Q111_M_6m_striatum_na_na
PD
na
PD_MPTP−HCl_na_na_ neurons_na_na
SCA
na
SCA_ATXN182Q_na_28w_cerebellum_na_na
SCA_ATXN130Q−D776_na_12w_cerebellum_na_na
SCA_ATXN182Q_na_12w_cerebellum_na_na
SCA_ATXN130Q−D776_na_5w_cerebellum_na_na
SCA_ATXN130Q−D776_na_28w_cerebellum_na_na
SCA_ATXN182Q_na_5w_cerebellum_na_na
SCA_ATXN130Q_na_5w_cerebellum_na_na
abs(correlation)
0.1
0.2
0.3
0.4
0.5
Correlation
−0.25 0.00 0.25 0.50

Figure S16. Transcriptome-wide Correlation Analysis with Neurodegenerative Mouse Models and the Kat5 cKO in the Endolysosome Subdomains.

The neurodegenerative mouse models representing AD and other neurological disease states are compared in transcriptome wide correlation analysis with the Kat5 cKO. There is strong positive correlation across numerous APP and Tau AD models, again with the strongest correlation observed with the p25/cdk5 mouse model. The other disease state models show far more model-specific effects, with one CJD model presenting positive correlation in the top row and the second showing the reversed pattern. One FTD-ALS model demonstrates strong correlation across endolysosomal subdomains, with little signal in the other models. In HD, most of the signal is negative, evinced by the predominance of red dots in the HD box. The SCA models vary between strong positive correlation in specific subdomains to no correlation, with negative correlation in some subdomains.

Figure S17

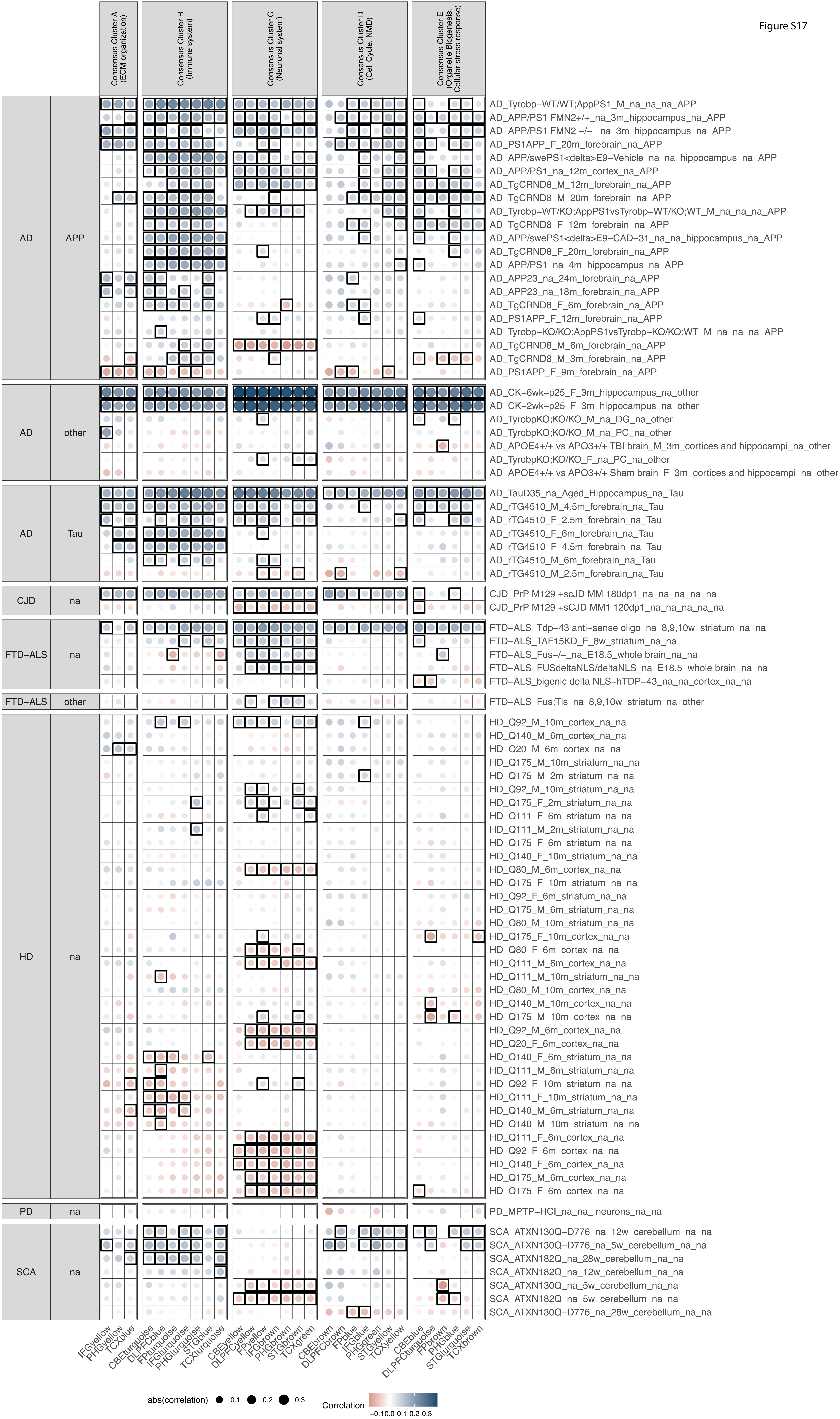
Consensus Cluster A (ECM organization)
Consensus Cluster B (Immune system)
Consensus Cluster C (Neuronal system)
Consensus Cluster D (Cell Cycle, NMD)
Consensus Cluster E (Organelle Biogenesis, Cellular stress response)
AD
APP
AD_Tyrobp−WT/WT;AppPS1_M_na_na_na_APP
AD_APP/PS1 FMN2+/+_na_3m_hippocampus_na_APP
AD_APP/PS1 FMN2 −/− _na_3m_hippocampus_na_APP
AD_PS1APP_F_20m_forebrain_na_APP
AD_APP/swePS1<delta>E9−Vehicle_na_na_hippocampus_na_APP
AD_APP/PS1_na_12m_cortex_na_APP
AD_TgCRND8_M_12m_forebrain_na_APP
AD_TgCRND8_M_20m_forebrain_na_APP
AD_Tyrobp−WT/KO;AppPS1vsTyrobp−WT/KO;WT_M_na_na_na_APP
AD_TgCRND8_F_12m_forebrain_na_APP
AD_APP/swePS1<delta>E9−CAD−31_na_na_hippocampus_na_APP
AD_TgCRND8_F_20m_forebrain_na_APP
AD_APP/PS1_na_4m_hippocampus_na_APP
AD_APP23_na_24m_forebrain_na_APP
AD_APP23_na_18m_forebrain_na_APP
AD_TgCRND8_F_6m_forebrain_na_APP
AD_PS1APP_F_12m_forebrain_na_APP
AD_Tyrobp−KO/KO;AppPS1vsTyrobp−KO/KO;WT_M_na_na_na_APP
AD_TgCRND8_M_6m_forebrain_na_APP
AD_TgCRND8_M_3m_forebrain_na_APP
AD_PS1APP_F_9m_forebrain_na_APP
AD
other
AD_CK−6wk−p25_F_3m_hippocampus_na_other
AD_CK−2wk−p25_F_3m_hippocampus_na_other
AD_TyrobpKO;KO/KO_M_na_DG_na_other
AD_TyrobpKO;KO/KO_M_na_PC_na_other
AD_APOE4+/+ vs APO3+/+ TBI brain_M_3m_cortices and hippocampi_na_other
AD_TyrobpKO;KO/KO_F_na_PC_na_other
AD_APOE4+/+ vs APO3+/+ Sham brain_F_3m_cortices and hippocampi_na_other
AD
Tau
AD_TauD35_na_Aged_Hippocampus_na_Tau
AD_rTG4510_M_4.5m_forebrain_na_Tau
AD_rTG4510_F_2.5m_forebrain_na_Tau
AD_rTG4510_F_6m_forebrain_na_Tau
AD_rTG4510_F_4.5m_forebrain_na_Tau
AD_rTG4510_M_6m_forebrain_na_Tau
AD_rTG4510_M_2.5m_forebrain_na_Tau
CJD
na
CJD_PrP M129 +scJD MM 180dp1_na_na_na_na_na
CJD_PrP M129 +sCJD MM1 120dp1_na_na_na_na_na
FTD−ALS
na
FTD−ALS_Tdp−43 anti−sense oligo_na_8,9,10w_striatum_na_na
FTD−ALS_TAF15KD_F_8w_striatum_na_na
FTD−ALS_Fus−/−_na_E18.5_whole brain_na_na
FTD−ALS_FUSdeltaNLS/deltaNLS_na_E18.5_whole brain_na_na
FTD−ALS_bigenic delta NLS−hTDP−43_na_na_cortex_na_na
FTD−ALS
other
FTD−ALS_Fus;Tls_na_8,9,10w_striatum_na_other
HD
na
HD_Q92_M_10m_cortex_na_na
HD_Q140_M_6m_cortex_na_na
HD_Q20_M_6m_cortex_na_na
HD_Q175_M_10m_striatum_na_na
HD_Q175_M_2m_striatum_na_na
HD_Q92_M_10m_striatum_na_na
HD_Q175_F_2m_striatum_na_na
HD_Q111_F_6m_striatum_na_na
HD_Q111_M_2m_striatum_na_na
HD_Q175_F_6m_striatum_na_na
HD_Q140_F_10m_striatum_na_na
HD_Q80_M_6m_cortex_na_na
HD_Q175_F_10m_striatum_na_na
HD_Q92_F_6m_striatum_na_na
HD_Q175_M_6m_striatum_na_na
HD_Q80_M_10m_striatum_na_na
HD_Q175_F_10m_cortex_na_na
HD_Q80_F_6m_cortex_na_na
HD_Q111_M_6m_cortex_na_na
HD_Q111_M_10m_striatum_na_na
HD_Q80_M_10m_cortex_na_na
HD_Q140_M_10m_cortex_na_na
HD_Q175_M_10m_cortex_na_na
HD_Q92_M_6m_cortex_na_na
HD_Q20_F_6m_cortex_na_na
HD_Q140_F_6m_striatum_na_na
HD_Q111_M_6m_striatum_na_na
HD_Q92_F_10m_striatum_na_na
HD_Q111_F_10m_striatum_na_na
HD_Q140_M_6m_striatum_na_na
HD_Q140_M_10m_striatum_na_na
HD_Q111_F_6m_cortex_na_na
HD_Q92_F_6m_cortex_na_na
HD_Q140_F_6m_cortex_na_na
HD_Q175_M_6m_cortex_na_na
HD_Q175_F_6m_cortex_na_na
PD
na
PD_MPTP−HCI_na_na_ neurons_na_na
SCA
na
SCA_ATXN130Q−D776_na_12w_cerebellum_na_na
SCA_ATXN130Q−D776_na_5w_cerebellum_na_na
SCA_ATXN182Q_na_28w_cerebellum_na_na
SCA_ATXN182Q_na_12w_cerebellum_na_na
SCA_ATXN130Q_na_5w_cerebellum_na_na
SCA_ATXN182Q_na_5w_cerebellum_na_na
SCA_ATXN130Q−D776_na_28w_cerebellum_na_na
IFGyellow
PHGyellow
TCXblue
CBEturquoise
DLPFCblue
FPturquoise
IFGturquoise
PHGturquoise
STGblue
TCXturquoise
CBEyellow
DLPFCyellow
FPyellow
IFGbrown
PHGbrown
STGbrown
TCXgreen
CBEbrown
DLPFCbrown
FPblue
IFGblue
PHGgreen
STGyellow
TCXyellow
CBEblue
DLPFCturquoise
FPbrown
PHGblue
STGturquoise
TCXbrown
abs(correlation)
0.1
0.2
0.3
Correlation
−0.1
0.0
0.1
0.2
0.3

Figure S17. Correlation Analysis of the Consensus Co-expression Clusters Across Neurodegenerative Mouse Models.

In previous work by Wan et al, co-expression mapping was performed transcriptome wide to assess common clusters of dysregulated genes across neurodegenerative diseases. The individually identified modules group into five consensus clusters (A-E) represented at the top of the figure, which broadly span several areas of neurobiology: extracellular matrix (ECM), immune system, neuronal system, cell cycle, and organelle biogenesis and stress response respectively. We employed these clusters to identify shared signal with the Kat5 cKO mouse models across disease groups. The disease and genetic driver are shown on the left side, the specific mouse models are shown on the right, with the clusters at the top and modules that comprise them arrayed along the bottom. There is strong correlation between the AD mouse models driven by APP and Tau with the Kat5 cKO. The p25/cdk5 mouse model demonstrates the strongest correlation with the Kat5 cKO across clusters and modules. CJD models are split, with the top model showing positive correlation with Kat5 and the bottom model demonstrate negative correlation within the cluster C (neuronal systems). There is positive correlation within cluster C within the FTD-ALS models, with much more variable results within the other clusters. The HD, PD and SCA models show mixed signal across clusters with some models demonstrating positive correlation and others negative, and many showing no signal at all. The scale for the correlation evaluation is represented at the bottom of the figure.

Figure S18

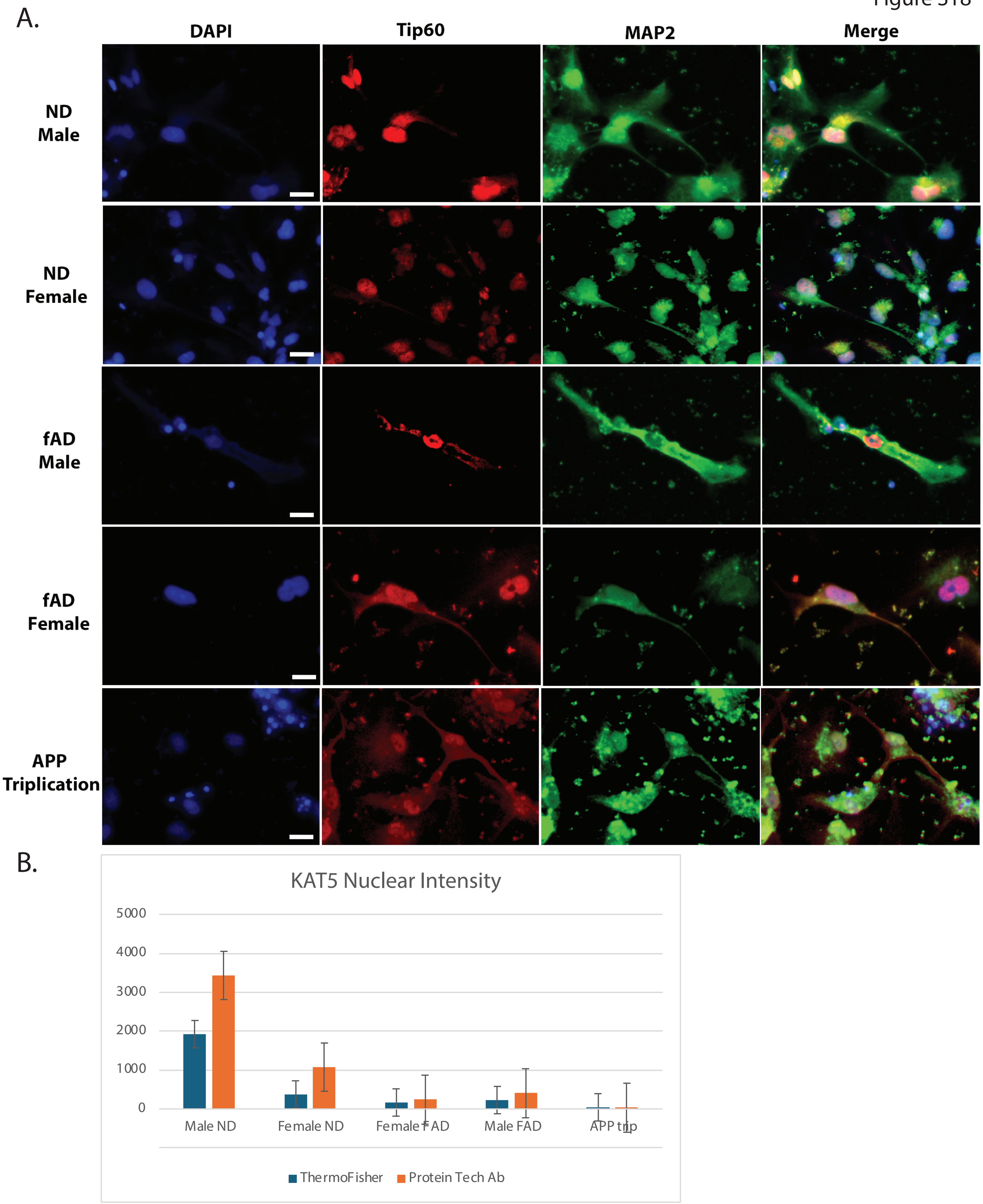
A.
DAPI
Tip60
MAP2
Merge
ND Male
ND Female
fAD Male
fAD Female
APP Triplication
B.
KAT5 Nuclear Intensity
5000
4000
3000
2000
1000
0
Male ND
Female ND
Female FAD
Male FAD
APP trip
ThermoFisher
Protein Tech Ab

Figure S18. KAT5 Subcellular Localization in FAD Neurons

Male and Female non-disease (ND), Male and Female FAD (APPV717I) and an APP triplication mutant hiPSC derived neurons were grown from neural progenitors into glutamatergic neurons for a week. (A) The neurons were then fixed and labeled with DAPI (blue) to show nuclear compartment, KAT5/Tip60 (red), or MAP2 to show cellular morphology. Two separate antibodies were employed to label KAT5/Tip60, one from ThermoFisher and one from Protein Tech (see methods for item numbers). The red KAT5 signal shows nuclear staining in the ND cell lines. The nuclear signal in the FAD neurons is weaker, and the staining appears more diffuse. The nuclear staining was identified by the region of interest designated by the DAPI channel, and automated quantitation was performed. (B) The quantitation was done in all cell lines with both antibodies and the average across all cells quantitated is shown with standard error. The ND cell lines have significantly greater nuclear signal than the FAD cell lines.

Figure S19

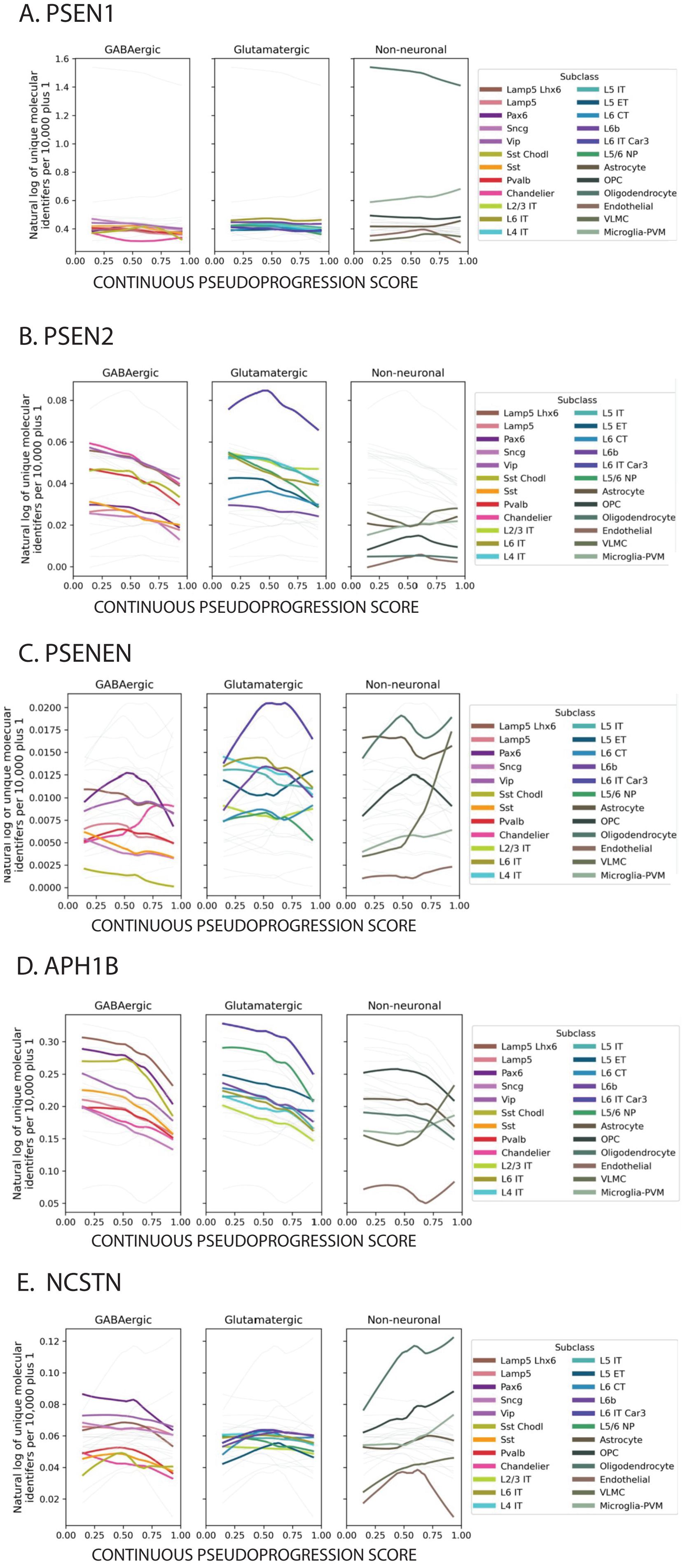
A. PSEN1
GABAergic
Glutamatergic
Non-neuronal
Natural log of unique molecular identifers per 10,000 plus 1
1.6
1.4
1.2
1.0
0.8
0.6
0.4
0.00 0.25 0.50 0.75 1.00
CONTINUOUS PSEUDOPROGRESSION SCORE
Subclass
Lamp5 Lhx6
Lamp5
Pax6
Sncg
Vip
Sst Chodl
Sst
Pvalb
Chandelier
L2/3 IT
L6 IT
L4 IT
L5 IT
L5 ET
L6 CT
L6b
L6 IT Car3
L5/6 NP
Astrocyte
OPC
Oligodendrocyte
Endothelial
VLMC
Microglia-PVM
B. PSEN2
GABAergic
Glutamatergic
Non-neuronal
Natural log of unique molecular identifers per 10,000 plus 1
0.08
0.06
0.04
0.02
0.00
0.00 0.25 0.50 0.75 1.00
CONTINUOUS PSEUDOPROGRESSION SCORE
Subclass
Lamp5 Lhx6
Lamp5
Pax6
Sncg
Vip
Sst Chodl
Sst
Pvalb
Chandelier
L2/3 IT
L6 IT
L4 IT
L5 IT
L5 ET
L6 CT
L6b
L6 IT Car3
L5/6 NP
Astrocyte
OPC
Oligodendrocyte
Endothelial
VLMC
Microglia-PVM
C. PSENEN
GABAergic
Glutamatergic
Non-neuronal
0.0200
0.0175
0.0150
0.0125
0.0100
0.0075
0.0050
0.0025
0.0000
0.00 0.25 0.50 0.75 1.00
CONTINUOUS PSEUDOPROGRESSION SCORE
Subclass
Lamp5 Lhx6
Lamp5
Pax6
Sncg
Vip
Sst Chodl
Sst
Pvalb
Chandelier
L2/3 IT
L6 IT
L4 IT
L5 IT
L5 ET
L6 CT
L6b
L6 IT Car3
L5/6 NP
Astrocyte
OPC
Oligodendrocyte
Endothelial
VLMC
Microglia-PVM
D. APH1B
GABAergic
Glutamatergic
Non-neuronal
Natural log of unique molecular identifers per 10,000 plus 1
0.30
0.25
0.20
0.15
0.10
0.05
0.00 0.25 0.50 0.75 1.00
CONTINUOUS PSEUDOPROGRESSION SCORE
Subclass
Lamp5 Lhx6
Lamp5
Pax6
Sncg
Vip
Sst Chodl
Sst
Pvalb
Chandelier
L2/3 IT
L6 IT
L4 IT
L5 IT
L5 ET
L6 CT
L6b
L6 IT Car3
L5/6 NP
Astrocyte
OPC
Oligodendrocyte
Endothelial
VLMC
Microglia-PVM
E. NCSTN
GABAergic
Glutamatergic
Non-neuronal
Natural log of unique molecular identifers per 10,000 plus 1
0.12
0.10
0.08
0.06
0.04
0.02
0.00 0.25 0.50 0.75 1.00
CONTINUOUS PSEUDOPROGRESSION SCORE
Subclass
Lamp5 Lhx6
Lamp5
Pax6
Sncg
Vip
Sst Chodl
Sst
Pvalb
Chandelier
L2/3 IT
L6 IT
L4 IT
L5 IT
L5 ET
L6 CT
L6b
L6 IT Car3
L5/6 NP
Astrocyte
OPC
Oligodendrocyte
Endothelial
VLMC
Microglia-PVM

Figure S19. Gamma-Secretase Components Dysregulated in Neurons in SEA-AD LOAD Study

The Seattle Alzheimer's Disease (SEA-AD) study performed expression analysis across AD progression as modeled by the continuous pseudo-progression score (CPS) within all identified cellular subtypes within the human brain taxonomy. The gamma-secretase components are shown depicting cellular subtypes within the GABAergic inhibitory neurons(left), glutamatergic excitatory neurons (middle) and non-neuronal CNS lineages (right). The homologues of presenilin PSEN1(A) and PSEN2(B) levels across disease are shown in the top two rows. The remaining three components of the heterotetrameric gamma-secretase complex are shown below (C) PSENEN, (D) APH1B, and (E) NCSTN. There is strong down-regulation of PSEN2 and APH1B within both inhibitory and excitatory neurons, while PSENEN and NCSTN decrease in later disease state only in GABAergic neurons. The non-neuronal cell types shown in the right-hand column appear to be stable or increase in most lineages.

Figure S20

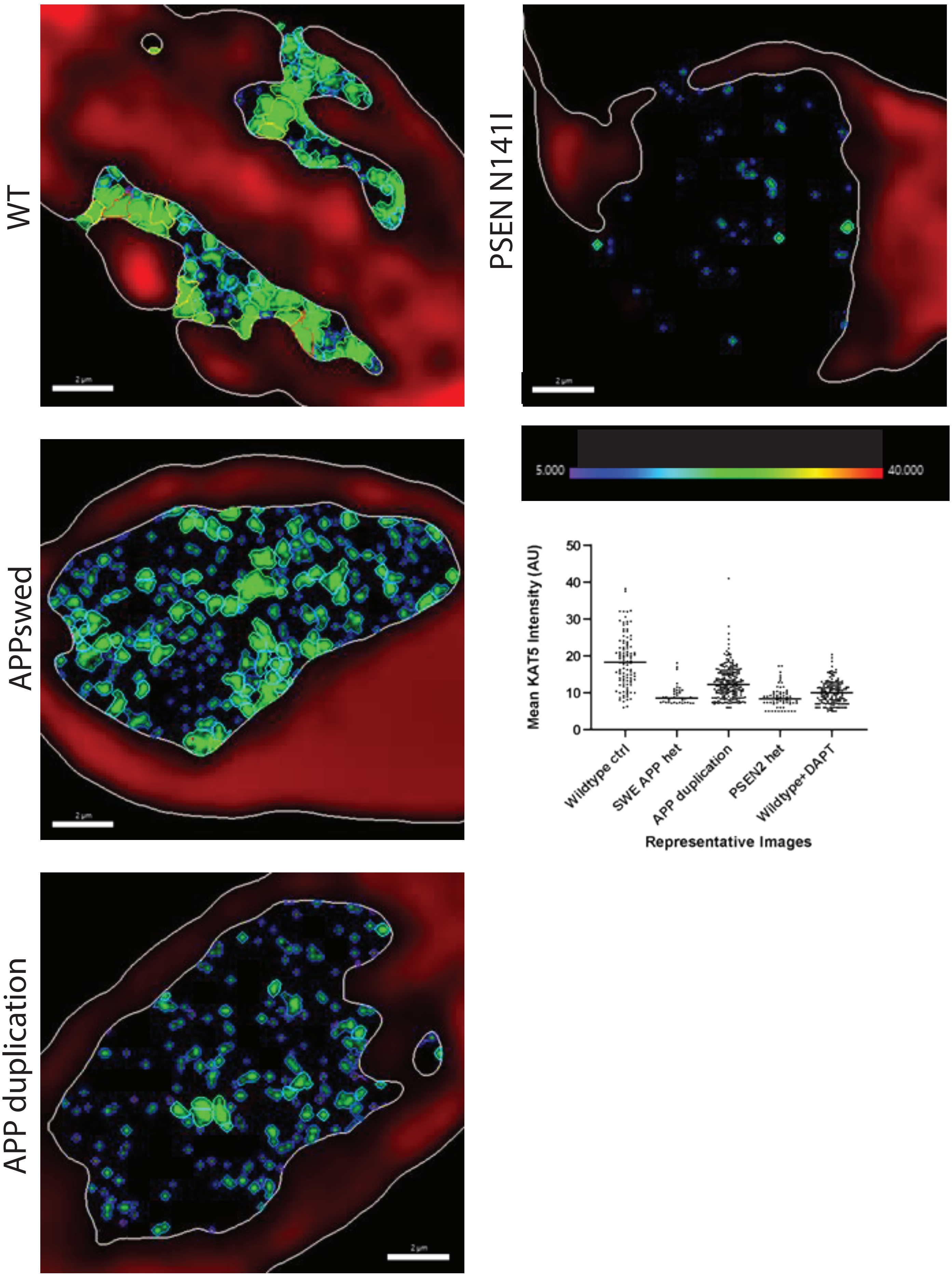
WT
PSEN N141I
APPswed
APP duplication
5.000
40.000
Mean KAT5 Intensity (AU)
50
40
30
20
10
0
Wildtype ctrl
SWE APP het
APP duplication
PSEN2 het
Wildtype+DAPT
Representative Images

Figure S20 Legend. Quantitation of Nuclear Kat5 Signal w Imaris Imaging System

Neuronal nuclei were identified by the presence of DAPI (blue) and the absence of MAP2 (red) cytoplasmic staining. A surface object workflow was used in Imaris to define the DAPI+ nuclear region of interest (ROI). The nuclear ROI surface was used to mask the KAT5 (green) channel and isolate nuclear KAT5 staining. A surface object workflow was run on the masked KAT5 channel to quantify nuclear KAT5 fluorescent intensity in the 12 imaged neurons. The segmentation lines surrounding the KAT5 puncta are color-coded to reflect the mean KAT5 intensity (AU) within these segmentations and these data are graphed in the lower right panel. KAT5 signal identification, segmentation and quantification was carried out identically across all images using these sets of parameters provided.
